# Gray and white matter morphology in substance use disorders: a neuroimaging systematic review and meta-analysis

**DOI:** 10.1038/s41398-020-01128-2

**Published:** 2021-01-11

**Authors:** Victor Pando-Naude, Sebastian Toxto, Sofia Fernandez-Lozano, Christine E. Parsons, Sarael Alcauter, Eduardo A. Garza-Villarreal

**Affiliations:** 1 Department of Clinical Medicine, Center for Music in the Brain, University of Aarhus, Aarhus, Denmark; 2 Instituto de Neurobiología, Universidad Nacional Autónoma de México (UNAM) campus Juriquilla, Queretaro, Mexico; 3 Instituto Nacional de Psiquiatría “Ramón de la Fuente Muñiz”, Mexico City, Mexico; 4 Facultad de Medicina, Universidad Nacional Autónoma de México (UNAM), Mexico City, Mexico; 5 Facultad de Psicología, Universidad Nacional Autónoma de México (UNAM), Mexico City, Mexico; 6 Department of Clinical Medicine, Interacting Minds Center, University of Aarhus, Aarhus, Denmark; 7 Department of Clinical Medicine, Center of Functionally Integrative Neuroscience, University of Aarhus, Aarhus, Denmark

**Keywords:** Addiction, Prognostic markers

## Abstract

Substance use disorders (SUDs) are characterized by a compulsion to seek and consume one or more substances of abuse, with a perceived loss of control and a negative emotional state. Prolonged substance use seems to be associated with morphological changes of multiple neural circuits, in particular the frontal–striatal and limbic pathways. Such neuroadaptations are evident across several substance disorders, but may vary depending on the type of substance, consumption severity and/or other unknown factors. We therefore identified studies investigating the effects of SUDs using volumetric whole-brain voxel-based morphometry (VBM) in gray (GM) and white matter (WM). We performed a systematic review and meta-analysis of VBM studies using the anatomic likelihood estimation (ALE) method implemented in GingerALE (PROSPERO pre-registration CRD42017071222). Sixty studies met inclusion criteria and were included in the final quantitative meta-analysis, with a total of 614 foci, 94 experiments and 4938 participants. We found convergence and divergence in brain regions and volume effects (higher vs. lower volume) in GM and WM depending on the severity of the consumption pattern and type of substance used. Convergent pathology was evident across substances in GM of the insula, anterior cingulate cortex, putamen, and thalamus, and in WM of the thalamic radiation and internal capsule bundle. Divergent pathology between occasional use (cortical pathology) and addiction (cortical-subcortical pathology) provides evidence of a possible top-down neuroadaptation. Our findings indicate particular brain morphometry alterations in SUDs, which may inform our understanding of disease progression and ultimately therapeutic approaches.

## Introduction

Substance use disorders (SUDs) refer to a wide range of alterations produced by the consumption of abuse substances or drugs. According to the Diagnostic and Statistical Manual of Mental Disorders (DSM-V)^1^, these substances include: alcohol, caffeine, cannabis, hallucinogens, inhalants, opioids, sedatives, hypnotics and anxiolytics, stimulants, tobacco, and other. About 275 million people worldwide (5.6% of the global population aged 15–64 years) used substances at least once during 2016^2^ and SUDs are recognized as a major public health issue. SUDs affect the reward system, involved in the reinforcement of behaviors and memory, and can lead to chronic use and dependency^3^. Initial substance reward is triggered by dopamine neurons in the ventral tegmental area (VTA), which project to the prefrontal cortex, amygdala and nucleus accumbens (NAc)^4,5^, as well as other ascending monoamine fibers such as norepinephrine and other non-dopaminergic systems within frontal regions^6^.

Additionally, dopaminergic neurons in substantia nigra pars compacta (SNc) project to the dorsal striatum (nigrostriatal pathway), a pathway implicated in the emergence of habits^7^. A reinforcement effect seems to depend on dopaminergic signaling in the NAc, and chronic use has been associated to neuroadaptations of the striato-thalamo-cortical (prefrontal cortex, orbitofrontal cortex and the anterior cingulate cortex) and limbic pathways (amygdala and hippocampus)^4,5^, especially in individuals who may be vulnerable due to genetic and/or environmental factors^8^. Other endogenous systems, such as the opioid and cannabinoid systems, may contribute to the reinforcement effect by modulating hedonic responses or inhibiting negative affective states^9^.

Substance-induced neuroadaptations are similar to synaptic changes associated with learning, including changes in dendritic morphology and ionotropic glutamate receptors (e.g., AMPA/NMDA), which result in long-term potentiation (LTP) and long-term depression (LTD)^10,11^. Notably, the link between repeated dopaminergic signaling and neuroadaptations is yet unclear, and causality should be interpreted with caution. These neuroadaptations result in pathological changes in brain morphology, that seem to be salient enough to be observed macroscopically with magnetic resonance imaging (MRI), as shown by neuroimaging studies in humans and animal models^12,13^.

Neuroimaging studies using MRI have shown alterations in gray and white matter in SUDs^14,15^. However, the involved regions vary widely and seem to depend on the type of substance, the consumption severity, the age of first use, the total time of usage, and other associated comorbidities. Morphometric studies investigating the effects of SUDs using volumetric measures such as voxel-based morphometry (VBM), have reported both lower and higher volume in cortical and subcortical gray matter (GM)^16,17^ and white matter (WM)^18,19^. For example, alcohol use disorder (AUD) studies have shown lower GM volume of the amygdala, insula, cingulate gyrus, orbitofrontal gyrus and thalamus^14^, while tobacco use disorder (TUD) studies have shown lower GM volume in thalamus, cingulate gyrus, prefrontal cortex, and cerebellum^20^. Cocaine use disorder (CUD) studies have shown lower GM volume in thalamus, insula, orbitofrontal cortex, anterior cingulate cortex, superior temporal cortex, and cerebellum^21^. Conversely, other studies of these same substances have shown higher GM volume in putamen and other nuclei of the basal ganglia^22,23^. Similarly, WM studies have shown different substances affecting different areas in distinct manners. For example, studies of AUD, TUD, and CUD have shown lower volume of WM in the corticospinal tract, thalamic radiations, and the corpus callosum^20,24–26^. Overall, the structural pathology seems to be both convergent and divergent in terms of localization between studies.

Given these findings, it is unclear how SUDs affect brain morphology and how to differentiate between distinct changes caused by substance toxicity and substance dependency^27^. Potential reasons for the variability in findings may include: (1) study definitions (substance use disorder vs addiction vs dependency), (2) polysubstance use, (3) the substance user characteristics, such as age or time of substance use, and (4) methodological differences between morphometric studies (i.e., software used). Thus, a meta-analysis of brain imaging studies provides an opportunity to better understand the mechanisms by which SUDs affect brain morphology, of great interest for treatment follow-up as well as potential marker of therapy success. In this systematic review and meta-analysis of VBM studies, we aimed at finding the overall effect of SUDs in GM and WM volume, and to differentiate the possible mechanisms behind such effects by means of subgroup analyses of the type of substance, consumption severity, age and associated comorbidities.

## Materials and methods

### Literature search, screening, and extraction

This systematic review and meta-analysis followed procedures from the Cochrane Handbook for Systematic Reviews^28^, and from the Center for Reviews and Dissemination (https://www.york.ac.uk/crd/). The review protocol was pre-registered in PROSPERO (CRD42017071222). This review was carried in accordance with the PRISMA^29^. We conducted a systematic literature search in PubMed, Scopus and PsycInfo, using both keywords and MeSH terms for articles published up to August 10th, 2020. No restrictions were placed on study design, but in order to be eligible for inclusion, the studies must have reported whole-brain VBM analyses. Screening and data extraction were performed using the Covidence tool^30^. The main outcome to extract was any change in gray and/or white matter analyzed using VBM, in stereotactic coordinates, comparing a substance user group and a healthy control group (details in Supplementary information).

### Quality assessment of MRI studies

Criteria for MRI quality reporting was selected from a set of guidelines for the standardized reporting of MRI studies^31–33^. Such guidelines dictate a more consistent and coherent policy for the reporting of MRI methods to ensure that methods can be understood and replicated.

### Analysis and meta-analytic technique

Statistically significant foci from between-group contrasts were extracted and recorded for each study. Where necessary, coordinates were converted from Talairach coordinates to MNI space using the Lancaster transform (icbm2tal) incorporated in GingerALE^34,35^. All meta-analyses were performed using anatomic likelihood estimation (ALE), implemented in GingerALE, in BrainMap^36^. This method extracts the coordinates from the included studies and tests for anatomical consistency and concordance between the studies. The coordinates are weighted according to the size of the sample (number of participants), and these weightings contribute to form estimates of anatomic likelihood estimation for each intracerebral voxel on a standardized map. This approach treats anatomic foci (input) not as single points, but as spatial probability distributions centered at the given coordinates. Therefore, the algorithm tests to what extent the spatial locations of the foci correlate across independently conducted MRI studies investigating the same construct, and assesses them against a null-distribution of random spatial association between experiments^37^. Statistical significance of the ALE scores was determined by a permutation test using cluster-level inference at *p* < 0.05 (FWE). As we did not impose any minimum cluster size of supra-threshold voxels, small volume clusters should be interpreted with caution.

The primary outcome was morphological brain differences measured by VBM between substance users (SU) and healthy controls (HC), pooling all substances together, to examine comprehensively the structural changes associated with SUD. To test the directionality of the primary outcome, we pooled coordinates reporting higher volume with substance use (HC < SU) and lower volume with substance use (SU < HC). Pre-registered subgroup analyses included age of substance users (adolescents vs. adults), consumption severity (addiction vs. long-term use vs. occasional use), type of substance (alcohol vs. tobacco vs. cannabis vs. cocaine vs. stimulants vs. opioids vs. ketamine vs. polysubstance; the latter refers to studies that combined substances into one main effect from the contrast SU vs. HC) and associated comorbidities (pure vs. dual). Finally, subgroups were tested for similarity (conjunction) and difference (subtraction) in a contrast analysis. All meta-analyses were conducted separately for GM and WM. We use “addiction” as a synonym for SUD that includes dependency, as the latter definition is fairly recent^1^. Additionally, addiction, long-term use and occasional use could also be regarded as severe-SUD, moderate-SUD, and mild-SUD, respectively.

We conducted meta-analytic connectivity modeling (MACM)^38^ to analyse co-activation patterns of regions-of-interest (ROI) resulting from the primary outcomes, aiming to functionally segregate each region’s putative contribution to behavioral domains^39^. Co-activation analyses were performed using Sleuth^40^ and GingerALE from the BrainMap database.

The meta-analytic results (ALE maps) were visualized using Mango on the MNI152 1 mm standard brain, and resulting coordinates were cross-referenced to the Harvard-Oxford Cortical and Subcortical Atlas and the Juelich Histological Atlas via NeuroVault^41^ and FSLeyes^42^, respectively.

Finally, we performed the Fail-Safe N analysis (FSN)^43^ as a measure of robustness against potential publication bias. It refers to the amount of contra-evidence that can be added to a meta-analysis before the results change, and can be obtained for each cluster that survives thresholding in an ALE meta-analysis. A higher FSN indicates more stable results and hence a higher robustness.

## Results

A total of 1095 records were identified through database searching, and after removing duplicates, 584 records were initially screened by title and abstract. A total of 584 articles were assessed for eligibility in the full-text screening stage. From these, 60 studies fulfilled criteria for eligibility and were included in both the qualitative and quantitative analyses (Supplementary Fig. 1).

### Characteristics of studies

The characteristics of studies included in the meta-analysis are shown in Table 1. Sixty studies met inclusion criteria and were included in the final quantitative meta-analysis, with a total of 614 foci and 94 experiments. The total number of participants was 4938, with 49.2% substance users (SU) and 50.8% heathy controls (HC). For the SU subsample, 64% in the addiction group (A), 7% on the long-term use group (LT), and 29% on the occasional use group (O). Alcohol was the main substance of interest in 20% of studies, tobacco 22%, cocaine 12%, cannabis 12%, opioids 12%, stimulants 6%, ketamine 2%, and polysubstance use 14%. SUD was evaluated by a psychiatrist in 27% of studies, psychologist 7%, clinician 2%, while 64% failed to report the evaluator. The DSM-IV was used in 70% of studies, DSM-V 7%, while 23% failed to report the tool used to diagnose substance use disorder. All of the studies reported change in GM volume (100%), while 15 studies (25%) reported change in WM volume.

**Table 1:** Characteristics of the studies included in meta-analysis.

															SU age		HC age		Years of use		Age of 1st use		Years of education (SU)		Years of education (HC)	
	Author	Year	Substance	SU	DSM	Assessed by:	GM/WM	A	LT	O	HC	Males	Females	Total	Mean	±SD	Mean	±SD	Mean	±SD	Mean	±SD	Mean	±SD	Mean	±SD
1	Almeida	2008	Tobacco	A	–	–	GM	39	–	–	39	50	28	78	75	3.4	75.7	3.2	59	8	15.6	5.1	–	–	–	–
2	Aoki	2013	Stimulant	O	IV	Psychiatrist	GM + WM	–	–	20	20	20	20	40	33.8	7.8	33.7	7.5	–	–	20.1	4.29	–	–	–	–
3	Bach	2019a	Opioid	A	–	Psychiatrist	GM	17	–	–	17	21	13	34	37.2	9.7	36.3	10.7	9.9	6.7	–	–	–	–	–	–
4	Bach	2019b	Opioid	A	–	Psychiatrist	GM	19	–	–	20	36	3	39	37.4	8.2	38.5	8.3	14.4	7.1	–	–	–	–	–	–
5	Banca	2016	Alcohol	O	–	–	GM	–	–	30	30	34	26	60	22.2	3.4	21.85	3.26	–	–	–	–	–	–	–	–
6	Barrós-	2011	Cocaine	A	IV	–	GM	20	–	–	16	36	0	36	33.3	6.9	33.38	9.17	13.2	5.97	19.6	5.97	9.2	1.7	8.3	1.5
7	Battistella	2014	Cannabis	LT	–	–	GM	–	25	–	22	47	0	47	23	2	25	2.5	7	2.75	15.75	2	15	–	15	–
8	Brody	2004	Tobacco	A	IV	–	GM	19	–	–	17	21	15	36	39.5	10.3	37.9	12.9	–	–	–	–	–	–	–	–
9	Bu	2016	Tobacco	A	V	–	GM	26	–	–	26	52	0	52	21.4	1.73	20.58	1.47	4.27	2.44	14.96	3.26	13.9	0.8	13.7	0.7
10	Chanraud	2009	Alcohol	A	IV	Psychiatrist	GM	24	–	–	24	48	0	48	47.8	7.7	45	5.6	9.2	8.9	39.75	10.33	7.6	3	8.7	3.4
11	Chanraud	2007	Alcohol	A	IV	Psychiatrist	GM + WM	26	–	–	24	50	0	50	47.7	7.1	45	6.72	8	6.3	39	11.1	7.58	2.96	8.7	3.36
12	Crunelle	2014	Cocaine	A	IV	–	GM	30	–	–	33	63	0	63	32	9	33	9	7	5	–	–	–	–	–	–
13	Daumann	2011	Stimulant	O	IV	–	GM	–	20	42	16	53	25	78	26.6	7.2	26.31	4.11	–	–	19.95	6.44	14.4	2.2	17.5	2.7
14	Demirakca	2011	Alcohol	A	IV	–	GM	50	–	–	66	61	55	116	46.6	8.2	45	10.1	12.4	7.4	–	–	–	–	–	–
15	Filbey	2014	Cannabis	LT	IV	–	GM	25	–	23	62	72	38	110	28.3	8.3	30	7.4	9.8	8	18.1	3.4	14.2	2.4	13.9	1.7
16	Franklin	2002	Cocaine	A	IV	–	GM + WM	13	–	–	16	29	0	29	42	6.3	32	6.9	13	6.5	–	–	12	1.1	17	2.6
17	Franklin	2014	Tobacco	A	IV	–	GM	80	–	–	80	42	38	160	33.9	11	32.1	7.4	14.1	10.1	14	–	14.1	2	13.6	2.2
18	Fritz	2014	Tobacco	A	–	–	GM + WM	–	–	315	659	391	583	974	44.1	11.8	51.49	14.45	–	–	17.3	4.9	–	–	–	–
19	Galandra	2018	Alcohol	A	V	–	GM	23	–	–	18	24	17	41	45.7	7.8	44.8	8.9	10.8	7.2	–	–	10	2.6	10.1	2.8
20	Galandra	2020	Alcohol	A	–	–	GM	22	–	–	18	–	–	40	45.6	8	44.8	9	10.1	6.6	–	–	9.9	2.7	10.1	2.8
21	Gallinat	2006	Tobacco	A	IV	Psychiatrist	GM + WM	–	–	22	23	24	21	45	30.8	7.5	30.3	7.9	13.9	7.3	16.2	1.8	–	–	–	–
22	Gardini	2012	Poly	A	IV	–	GM + WM	–	38	–	24	–	–	62	31.2	5.4	33.21	7.06	12.3	5.3	–	–	–	–	–	–
23	Gilman	2014	Cannabis	O	IV	–	GM + WM	–	–	20	20	18	22	40	21.3	1.9	20.7	1.9	6.21	3.43	16.6	2.13	12.6	4.8	14.3	3.4
24	Grodin	2013	Poly	A	IV	–	GM	130	–	–	69	130	69	199	40	9	36.6	1.1	11.1	7.3	24	8.2	13.2	2.3	16.8	0.3
25	Hanlon	2014	Tobacco	A	–	–	GM	62	–	–	60	70	52	122	40	–	36	–	22.9	6.7	16.2	1.5	21.9	0.6	22.3	0.4
26	Hanlon	2016	Tobacco	A	–	–	GM	58	–	–	60	71	47	118	40	–	36	–	22.9	6.7	16.2	1.25	20.1	0.3	20.7	0.3
27	Jang	2007	Alcohol	A	IV	Psychiatrist	GM + WM	20	–	–	20	40	0	40	43.5	6	44.5	7.4	–	–	33.2	7.8	14.3	4.2	15.3	2.6
28	Jan	2012	Stimulant	A	IV	Psychiatrist	GM	17	–	–	20	25	12	37	30.9	8.2	35.1	6.6	10.2	5.8	23.9	6.6	–	–	–	–
29	Li	2019	Alcohol	A	–	–	GM	20	–	–	15	5	30	35	49	12	49	12	–	–	–	–	14	4	13	3
30	Liao	2011	Ketamine	A	IV	Psychiatrist	GM	–	41	–	44	67	18	85	26.9	4.9	26.3	5.84	3.43	1.79	23.1	5.21	15	2.6	11.9	2.8
31	Liao	2012	Tobacco	A	IV	Psychiatrist	GM	44	–	–	44	70	18	88	28.1	5.5	26.3	5.84	10.4	5.72	18	4.29	13.2	2.92	15	2.6
32	Lin	2012	Opioids	A	IV	–	GM	27	–	–	23	48	2	50	36.8	6.6	64.04	7.28	13.9	6.4	22.9	6.8	10.33	2	15.36	1.14
33	Liu	2009	Opioids	A	IV	–	GM	15	–	–	15	20	10	30	30.5	6.2	30.53	6.7	6.2	4.6	–	–	10.13	1.96	11.73	2.49
34	Lyoo	2006	Opioids	A	IV	–	GM	63	–	–	46	57	52	109	38.4	9.4	38.4	8.6	16.8	9.5	–	–	11.1	2.4	15.2	3.1
35	Mackey	2014	Poly	O	–	Clinician	GM	–	–	165	46	122	89	211	20.9	1.5	21.02	2.17	–	–	–	–	14.59	1.32	14.52	1.41
36	Matochik	2005	Cannabis	LT	IV	–	GM + WM	–	11	–	8	19	0	19	25.4	5	29.7	4.7	7.5	5.5	15.7	2.5	25.4	5	29.7	4.7
37	Matuskey	2014	Cocaine	A	IV	–	GM	14	–	–	14	20	8	28	41	6	41	8	21	7	–	–	–	–	–	–
38	Meade	2020	Cocaine	A	IV	–	GM	39	–	–	40	48	41	79	45.4	6.2	43.5	7.4	–	–	–	–	12.56	2.46	13.75	2.12
39	Mechtcheriakov	2007	Alcohol	A	–	–	GM + WM	22	–	–	22	28	16	44	53.6	–	53.7	–	–	–	–	–	9.7	2.6	10.1	2.3
40	Morales	2012	Poly	A	IV	–	GM	39	–	–	25	33	31	64	34.8	1.5	35.4	1.8	11.5	1.5	–	–	12.8	0.3	14.6	0.4
41	Moreno-A	2018	Cannabis	LT	IV	–	GM	14	–	–	28	12	30	42	30.1	5.2	31.3	7	14.4	7	17.1	3	–	–	–	–
42	Moreno-L	2012	Cocaine	A	IV	–	GM + WM	38	–	–	38	76	0	76	29.6	6.5	31.08	5.14	4.05	3.07	–	–	12.03	3.62	17.58	4.56
43	Mwansisya	2016	Opioids	A	IV	–	GM	15	–	–	15	20	10	30	30.5	6.2	30.6	6.77	6.21	4.63	–	–	10.4	1.55	11.87	2.5
44	Noyan	2016	Poly	A	IV	Psychiatrist	GM	46	–	–	30	76	0	76	27.4	7.1	29.07	5.5	–	–	–	–	11.48	3.01	29.07	5.5
45	Nurmedov	2015	Cannabis	LT	V	Psychiatrist	GM	–	20	–	20	40	0	40	24	4.7	25.85	3.62	–	–	21.5	4.8	9.75	2.67	11.1	2.94
46	Nurmedov	2016	Alcohol	A	V	Psychiatrist	GM	24	–	–	29	43	10	53	40.8	9.8	37.45	10.8	19	9.19	21.3	7.98	–	–	–	–
47	Peng	2015	Tobacco	A	–	–	GM + WM	53	–	–	53	106	0	106	30.7	4.2	30.83	5.18	–	–	19.04	3.94	19.21	1.39	19.32	1.29
48	Peng	2018	Tobacco	A	–	–	GM	53	–	–	53	106	0	106	30.7	4.2	30.8	5.2	–	–	19	4	19.21	1.39	19.32	1.29
49	Potvin	2007	Poly	A	IV	Psychiatrist	GM	12	–	–	15	19	8	27	25.5	5.2	25.8	2.9	–	–	–	–	–	–	–	–
50	Qiu	2013	Opioids	A	IV	Psychologist	GM	24	–	–	24	40	8	48	35.7	5.7	35.38	6.02	10.83	4.613	–	–	10.79	2.67	11.21	3.25
51	Qiu	2014	Opioids	O	IV	Psychologist	GM	30	–	–	30	56	4	60	25.1	3.1	23.97	2.47	5.08	1.8	19.93	12-30	13.03	2.76	12.07	3.42
52	Segobin	2014	Alcohol	A	IV	Psychologist	GM + WM	19	–	–	20	–	–	39	44.4	6.1	46.7	4.25	29.05	8.76	–	–	11.15	1.9	10.6	2.58
53	Sim	2007	Cocaine	A	IV	Psychologist	GM + WM	40	–	–	41	53	28	81	41.4	6.9	38.7	8.8	15.3	6.3	–	–	12.5	1.83	14.3	1.74
54	Stoeckel	2016	Tobacco	A	IV	–	GM	–	–	16	16	23	9	32	37.9	11.6	34.19	7.2	17.63	10.49	17.97	3.14	14.44	1.67	15.25	1
55	vanEijk	2013	Alcohol	A	IV	–	GM + WM	49	–	–	55	82	22	104	47	10.1	45.3	11.9	–	–	–	–	–	–	–	–
56	vanHolst	2012	Alcohol	A	IV	–	GM	36	–	–	54	90	0	90	43.2	11	35.3	10.1	11.69	9.7	–	–	–	–	–	–
57	Wang	2016	Alcohol	A	IV	Psychiatrist	GM	20	–	–	20	40	0	40	43.4	6.3	40.5	8.1	–	–	18.7	2.7	11.6	2.7	9.15	4.18
58	Wetherill	2015	Cannabis	A	IV	–	GM	61	–	–	21	52	30	82	28	7	31	9	8	6	20	6	13	1	13	1
59	Yip	2017	Poly	A	IV	–	GM	37	–	–	37	53	21	74	42.4	6.1	38	11.03	–	–	–	–	12.38	1.11	14.38	1.92
60	Zhang	2011	Tobacco	A	IV	Psychiatrist	GM	–	–	48	48	48	48	96	31.4	8.1	31.1	8.8	12.8	7.4	15.6	3.4	13.1	2.2	13.5	1.8

*SU* substance user, *HC* healthy control group, *DSM* Diagnostic and Statistical Manual of Mental Disorders, *A* addiction group, *LT* long-term use group, *O* occasional use group, *GM* gray matter, *WM* white matter.

Neuroimaging data was acquired in either 1.5 T (43%), or 3 T (57%) MRI scanners. Half of the studies were conducted in a Siemens MRI system, others were general electric (30%), Phillips (18%), and Bruker (2%). Most of the T1w-structural images were acquired using magnetization-prepared rapid acquisition with gradient echo sequence (MPRAGE), in 25 studies (42%), and 1 mm^3^-voxel size in 36 studies (60%). VBM analyses were conducted in either SPM^44^ (82%), FSL^42^ (18%), or AFNI^45^ (2%) (Supplementary Table 1).

### MRI quality

MRI quality of the included studies in the meta-analysis was assessed by a set of guidelines for the standardized reporting of MRI studies^31–33^ (Supplementary Table 2). All studies reported their MRI design, software package and image acquisition, processing and analyses. Overall, good MRI practices were performed in the included studies.

### Primary outcome

**Fig. 1:**
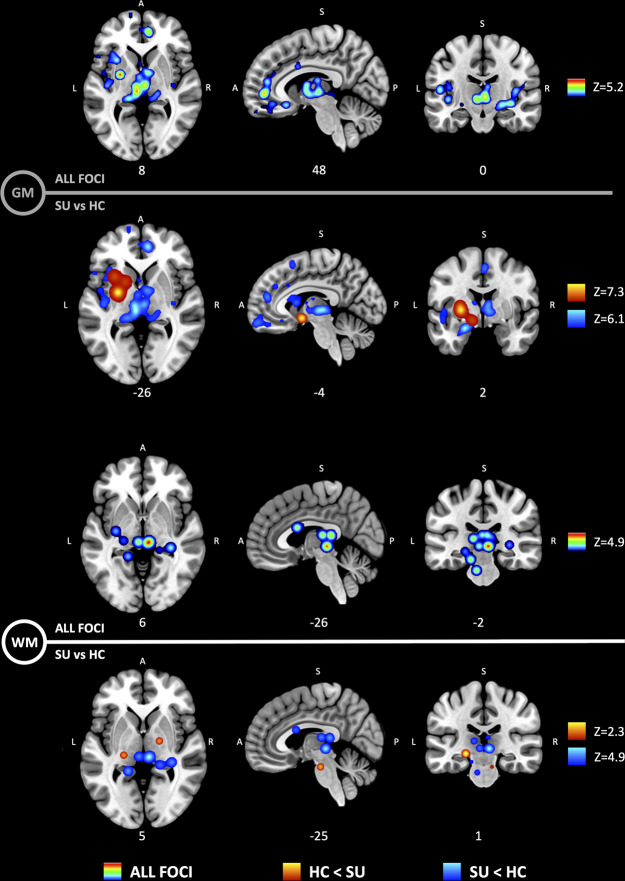
Anatomic likelihood estimation meta-analytic results for studies comparing brain morphological changes between SU and HC, at cluster level inference *p* < 0.05 (FWE). The primary outcome included GM (top) and WM (bottom) volumetric alterations in SUDs. HC < SU = higher volume with use; SU < HC = lower volume with use. Significant ALE maps show lower volume in thalamus, insula and anterior cingulate cortex in GM, and thalamic radiations in WM; and higher volume in putamen GM, and corticospinal WM tract. Such results support the idea that the entire limbic loop of the basal ganglia shows neuroadaptations produced by SUD. Z, peak Z-value.

The primary outcome was brain morphological differences measured by VBM between SU and HC, pooling all substances together, and defined as higher or lower volume. First, we included all substances and all reported coordinates and found three clusters in GM: right anterior cingulate cortex, left putamen and left thalamus; and one cluster in WM: right anterior thalamic radiation. Second, the comparison SU < HC (lower volume with use) resulted in three GM clusters: right anterior cingulate cortex, left thalamus and left insula; and one WM cluster: right anterior thalamic radiation. Finally, the comparison HC < SU (higher volume with use) resulted in one GM cluster: left putamen; and three WM clusters: right corticospinal tract, left superior longitudinal fasciculus and left optic radiation (Fig. 1 and Table 2).

**Table 2:** Anatomic likelihood estimation meta−analytic results for studies comparing brain morphological changes between SU and HC at cluster level inference *p* < 0.05 (FWE).

Cluster number	Volume (mm^3^)	MNI coordinates			ALE	*P*	*Z*	Label (Side region BA)
		*x*	*y*	*z*				
*a. GM: All foci*								
1	23,768	8	48	0	3E−02	1E−07	5.2	R Anterior cingulate cortex BA32
		0	48	−16	3E−02	8E−07	4.8	L Anterior cingulate cortex BA32
		−10	58	−16	3E−02	8E−06	4.3	L Superior frontal gyrus BA10
		−2	14	58	3E−02	8E−06	4.3	L Superior frontal gyrus BA6
		−2	36	30	2E−02	2E−05	4.1	L Medial frontal gyrus BA6
		0	24	46	2E−02	3E−05	4.1	L Medial frontal gyrus BA6
		−4	46	16	2E−02	3E−05	4.0	L Medial frontal gyrus BA9
		−2	20	32	2E−02	8E−05	3.8	L Anterior cingulate cortex BA32
		8	22	−14	2E−02	1E−04	3.7	R Anterior cingulate cortex BA32
		4	44	14	2E−02	2E−04	3.6	R Anterior cingulate cortex BA32
		16	20	−16	2E−02	3E−04	3.4	R Orbitofrontal cortex BA47
		2	0	52	2E−02	5E−04	3.3	L Medial frontal gyrus BA6
		6	38	−14	2E−02	2E−03	3.0	R Anterior cingulate cortex BA32
		6	8	30	1E−02	2E−03	2.8	R Anterior cingulate cortex BA24
		−14	64	−2	1E−02	7E−03	2.4	L Medial frontal gyrus BA10
		−4	56	−6	1E−02	8E−03	2.4	L Medial frontal gyrus BA10
		−14	68	0	1E−02	1E−02	2.3	L Superior frontal gyrus BA10
		−2	50	28	1E−02	1E−02	2.2	L Medial frontal gyrus BA9
		−2	62	−6	1E−02	2E−02	2.1	L Medial frontal gyrus BA10
		6	58	−22	1E−02	2E−02	2.1	R Medial frontal gyrus BA11
		14	38	−26	1E−02	2E−02	2.1	R Inferior frontal gyrus BA11
		28	20	−16	1E−02	2E−02	2.1	R Claustrum
		−12	14	66	1E−02	2E−02	2.1	L Superior frontal gyrus BA6
		8	8	38	1E−02	2E−02	2.0	R Cingulate gyrus BA24
2	23,256	−6	−24	0	4E−02	4E−08	5.4	L Thalamus
		2	−16	4	3E−02	6E−07	4.8	L Thalamus MDN
		34	−14	−6	2E−02	1E−05	4.2	R Putamen
		−16	−32	0	2E−02	4E−05	3.9	L Thalamus
		8	−2	0	2E−02	5E−05	3.9	R Thalamus
		6	−2	8	2E−02	7E−05	3.8	R Thalamus
		40	−18	10	2E−02	1E−04	3.7	R Insula BA13
		24	−16	−6	2E−02	1E−04	3.7	R Lateral globus pallidus
		−4	−8	0	2E−02	2E−04	3.5	L Thalamus
		18	−26	4	2E−02	3E−04	3.5	R Thalamus
		−6	10	10	2E−02	4E−04	3.3	L Caudate body
		6	−14	14	2E−02	5E−04	3.3	R Thalamus MDN
		48	−28	18	2E−02	5E−04	3.3	R Insula BA13
		16	−42	−10	2E−02	2E−03	2.9	R Culmen
		−12	−4	12	2E−02	2E−03	2.9	L Thalamus VAN
		−6	14	16	1E−02	6E−03	2.5	L Caudate body
		−4	10	2	1E−02	1E−02	2.2	L Caudate body
		−8	−12	14	1E−02	2E−02	2.0	L Thalamus
3	22,432	−26	−4	4	4E−02	1E−08	5.6	L Putamen
		−52	−16	12	2E−02	5E−05	3.9	L Transverse temporal gyrus BA41
		−44	−10	−6	2E−02	6E−05	3.8	L Insula BA13
		−30	14	2	2E−02	2E−04	3.5	L Claustrum
		−42	−16	0	2E−02	3E−04	3.4	L Insula BA13
		−26	−14	−6	2E−02	9E−04	3.1	L Lateral globus pallidus
		−16	18	−14	2E−02	1E−03	3.1	L Putamen
		−40	12	−2	1E−02	3E−03	2.8	L Insula BA13
		−10	28	−14	1E−02	3E−03	2.7	L Caudate head
		−40	−18	14	1E−02	3E−03	2.7	L Insula BA13
		−48	2	−8	1E−02	5E−03	2.6	L Superior temporal gyrus BA22
		−24	16	−8	1E−02	6E−03	2.5	L Putamen
		−36	8	−10	1E−02	6E−03	2.5	L Claustrum
		−28	8	−8	1E−02	8E−03	2.4	L Putamen
		−34	−8	−26	1E−02	8E−03	2.4	L Amygdala
		−46	−2	2	1E−02	1E−02	2.3	L Insula BA13
		−42	12	6	1E−02	1E−02	2.3	L Insula BA13
		−54	18	2	1E−02	1E−02	2.3	L Precentral gyrus BA44
		−48	16	−2	1E−02	1E−02	2.3	L Insula BA13
		−64	−4	−16	1E−02	1E−02	2.2	L Middle temporal gyrus BA21
		−58	−4	−16	1E−02	1E−02	2.2	L Middle temporal gyrus BA21
		−30	18	−18	1E−02	1E−02	2.2	L Inferior frontal gyrus BA47
		−38	−28	10	1E−02	2E−02	2.1	L Transverse temporal gyrus BA41
		−26	−10	−18	1E−02	2E−02	2.1	L Amygdala
		−32	16	−26	1E−02	2E−02	2.1	L Inferior frontal gyrus BA47
*b. GM: lower volume with use (SU* < *HC)*								
1	25,360	8	48	0	3E−02	8E−08	5.3	R Anterior cingulate BA32
		0	48	−16	3E−02	6E−07	4.9	R Anterior cingulate BA32
		−10	58	−16	3E−02	6E−06	4.4	L Superior frontal gyrus BA10
		−2	14	58	3E−02	6E−06	4.4	L Superior frontal gyrus BA6
		−2	36	30	2E−02	1E−05	4.2	L Medial frontal gyrus BA6
		0	24	46	2E−02	2E−05	4.1	R Medial frontal gyrus BA6
		−4	46	16	2E−02	2E−05	4.1	L Medial frontal gyrus BA9
		−2	20	32	2E−02	6E−05	3.8	L Cingulate gyrus BA32
		8	22	−14	2E−02	1E−04	3.7	R Anterior cingulate BA32
		4	44	14	2E−02	1E−04	3.7	R Anterior cingulate BA32
		2	0	52	2E−02	4E−04	3.3	R Medial frontal gyrus BA6
		6	38	−14	2E−02	1E−03	3.0	R Anterior cingulate BA32
		6	8	30	1E−02	2E−03	2.9	R Cingulate gyrus BA24
		−10	30	−14	1E−02	3E−03	2.8	L Anterior cingulate BA24
		−14	64	−2	1E−02	6E−03	2.5	L Medial frontal gyrus BA10
		−4	56	−6	1E−02	7E−03	2.5	L medial frontal gyrus BA10
		−14	68	0	1E−02	9E−03	2.4	L Superior frontal gyrus BA10
		2	36	−30	1E−02	1E−02	2.3	R Rectal gyrus BA11
		−2	50	28	1E−02	1E−02	2.3	L Medial frontal gyrus BA9
		−2	62	−6	1E−02	1E−02	2.2	L Medial frontal gyrus BA10
		6	58	−22	1E−02	1E−02	2.2	R Medial frontal gyrus BA11
		14	38	−26	1E−02	1E−02	2.2	R Inferior frontal gyrus BA11
		−12	14	66	1E−02	2E−02	2.1	L Superior Frontal Gyrus BA6
		28	20	−14	1E−02	2E−02	2.1	R Claustrum
		8	8	38	1E−02	2E−02	2.1	R Cingulate gyrus BA24
2	22,824	−6	−24	0	4E−02	3E−08	5.4	L Thalamus
		2	−16	4	3E−02	2E−06	4.6	R Thalamus
		−16	−32	0	2E−02	3E−05	4.0	L Thalamus
		8	−2	0	2E−02	4E−05	3.9	R Thalamus
		6	−2	8	2E−02	5E−05	3.9	R Thalamus
		32	−16	−6	2E−02	6E−05	3.9	R Lentiform nucleus
		40	−18	10	2E−02	8E−05	3.8	R Insula BA13
		24	−16	−6	2E−02	8E−05	3.8	R Lentiform nucleus
		18	−26	4	2E−02	2E−04	3.5	R Thalamus
		−4	−6	0	2E−02	3E−04	3.4	L Thalamus
		−6	10	10	2E−02	3E−04	3.4	L Caudate
		6	−14	14	2E−02	4E−04	3.4	R Thalamus
		48	−28	18	2E−02	4E−04	3.3	R Insula BA13
		−12	−4	12	2E−02	2E−03	3.0	L Thalamus
		−6	14	16	1E−02	5E−03	2.6	L Caudate
		0	−10	24	1E−02	1E−02	2.3	R Cingulate gyrus BA23
		−4	10	2	1E−02	1E−02	2.3	L Caudate
		20	−20	14	1E−02	1E−02	2.2	R Thalamus
		−8	−12	14	1E−02	2E−02	2.1	L Thalamus
3	17064	−44	−10	−6	2E−02	5E−05	3.9	L Insula BA13
		−42	−16	0	2E−02	2E−04	3.5	L Insula BA13
		−36	22	−2	1E−02	2E−03	2.9	L Insula BA13
		−40	12	−2	1E−02	2E−03	2.8	L Insula BA13
		−40	−18	14	1E−02	3E−03	2.8	L Insula BA13
		−48	2	−8	1E−02	4E−03	2.7	L Superior temporal gyrus BA22
		−22	14	−14	1E−02	6E−03	2.5	L Lentiform nucleus
		−24	16	−8	1E−02	7E−03	2.5	L Lentiform nucleus
		−38	10	−10	1E−02	7E−03	2.5	L Insula BA13
		−34	−8	−26	1E−02	7E−03	2.5	L Parahippocampal gyrus
		−46	−2	2	1E−02	9E−03	2.4	L Insula BA13
		−42	12	6	1E−02	9E−03	2.4	L Insula BA13
		−54	18	2	1E−02	1E−02	2.3	L Precentral gyrus BA44
		−48	16	−2	1E−02	1E−02	2.3	L Insula BA13
		−64	−4	−16	1E−02	1E−02	2.3	L Middle temporal gyrus BA21
		−58	−4	−16	1E−02	1E−02	2.3	L Middle temporal gyrus BA21
		−30	18	−18	1E−02	1E−02	2.3	L Inferior frontal gyrus BA47
		−60	2	−4	1E−02	1E−02	2.2	L Superior temporal gyrus
		−38	−28	10	1E−02	1E−02	2.2	L Transverse temporal gyrus BA41
		−32	16	−26	1E−02	2E−02	2.2	L Inferior frontal gyrus BA47
*c. GM: Higher volume with use (HC* < *SU)*								
1	22,440	−26	−4	2	3E−02	1E−13	7.3	L Putamen
		−14	4	−8	1E−02	5E−06	4.4	L Lateral globus pallidus
		−28	8	−8	1E−02	4E−05	4.0	L Putamen
		−30	14	2	1E−02	2E−04	3.6	L Claustrum
		−24	−12	−8	1E−02	2E−04	3.6	L Lateral globus pallidus
		−16	20	−16	9E−03	5E−04	3.3	L Subcallosal Gyrus BA47
*d. WM: All foci*								
1	37,328	6	−26	−2	2E−02	5E−07	4.9	R Anterior thalamic radiation
		8	−32	10	1E−02	5E−05	3.9	R Corpus callosum
		−6	−38	−14	1E−02	2E−04	3.6	L Corticospinal tract
		−12	−28	8	1E−02	2E−04	3.5	L Fornix
		−4	−26	−2	1E−02	2E−04	3.5	L Anterior thalamic radiation
		−4	2	24	1E−02	2E−04	3.5	L Corpus callosum
		2	10	22	1E−02	2E−04	3.5	R Corpus callosum
		−2	−30	12	1E−02	2E−04	3.5	L Corpus callosum
		6	−22	12	1E−02	3E−04	3.5	R Fornix
		32	−32	0	1E−02	3E−04	3.4	R Optic radiation
		−16	−42	2	1E−02	3E−04	3.4	L Cingulum
		−4	−14	18	1E−02	3E−04	3.4	L Fornix
		−8	−28	−30	1E−02	4E−04	3.4	L Corticospinal tract
		18	−34	4	9E−03	5E−04	3.3	R Fornix
		−16	−28	−18	9E−03	5E−04	3.3	L Cingulum
		−30	−14	−8	9E−03	9E−04	3.1	L Optic radiation
		−32	−10	−14	9E−03	1E−03	3.1	L Optic radiation
		−22	−24	−8	8E−03	1E−03	3.0	L Optic radiation
*e. WM: Lower volume with use (SU* < *HC)*								
1	33,624	6	−26	−2	2E−02	4E−07	4.9	R Anterior thalamic radiation
		8	−32	10	1E−02	5E−05	3.9	R Corpus callosum
		−6	−38	−14	1E−02	1E−04	3.6	L Corticospinal tract
		−12	−28	8	1E−02	2E−04	3.6	L Fornix
		−4	−26	−2	1E−02	2E−04	3.5	L Anterior thalamic radiation
		−4	2	24	1E−02	2E−04	3.5	L Corpus callosum
		2	10	22	1E−02	2E−04	3.5	R Corpus callosum
		−2	−30	12	1E−02	2E−04	3.5	L Corpus callosum
		6	−22	12	1E−02	2E−04	3.5	R Fornix
		32	−32	0	1E−02	3E−04	3.4	R Optic radiation
		−16	−42	2	1E−02	3E−04	3.4	L Cingulum
		−4	−14	18	1E−02	3E−04	3.4	L Fornix
		−8	−28	−30	1E−02	4E−04	3.4	L Corticospinal tract
		18	−34	4	9E−03	4E−04	3.3	R Fornix
		−14	−30	−18	9E−03	5E−04	3.3	L Cingulum
*f. WM: Higher volume with use (HC* < *SU)*								
1	27,736	14	−14	−16	2E−04	1E−02	2.3	R Corticospinal tract
2	14,712	−46	−6	−30	8E−03	2E−05	4.2	L Superior longitudinal fasciculus
3	14,712	−22	−24	−8	8E−03	2E−05	4.2	L Optic radiation

*GM* gray matter, *WM* white matter, *SU* substance user, *HC* healthy control, *ALE* anatomic likelihood estimation, *P* *p*-value, *Z* peak *z*-value, *R* right, *L* left.

#### Subgroup analyses

**Fig. 2:**
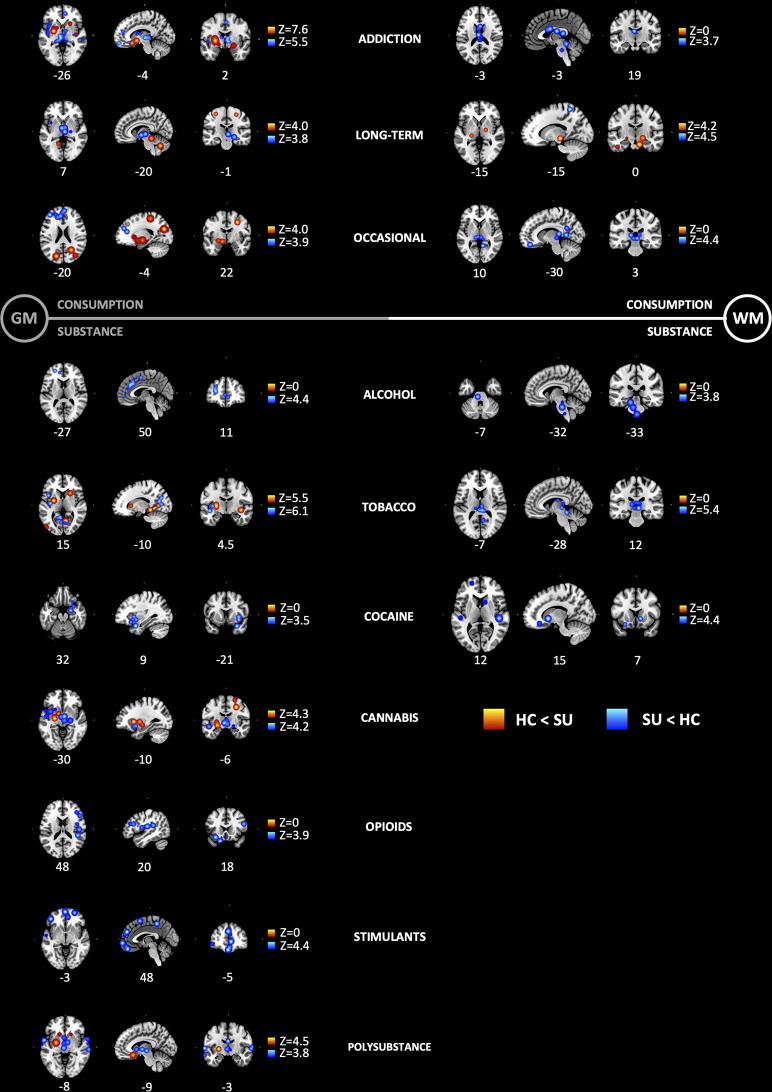
Subgroup anatomic likelihood estimation meta-analytic results for studies comparing brain morphological changes between SU and HC, at cluster level inference *p* < 0.05 (FWE). Subgroup analyses included consumption severity (top) and type of substance (bottom). HC < SU = higher volume with use; SU < HC = lower volume with use. Significant ALE maps showing lower GM and WM volumes across all types of consumption; higher GM volumes were also shown across all types of consumption; higher WM only in long-term use; lower GM volume in all substances, and higher GM volume only in tobacco, cannabis and polysubstance; lower WM volume in alcohol, tobacco and cocaine, and found no higher WM volume in any substance. Consumption: addiction (*k* = 49) vs. long-term use (*k* = 5) vs. occasional use (*k* = 6). Substance: alcohol (*k* = 14) vs. tobacco (*k* = 13) vs. cannabis (*k* = 7) vs. cocaine (*k* = 7) vs. stimulants (*k* = 3) vs. opioids (*k* = 8) vs. ketamine (*k* = 1), and papers that pooled together substances which we termed polysubstance (*k* = 7). *Z*, peak *Z*-value.

Pre-hoc subgroup analyses included (1) age of substance user: adolescents vs. adults; (2) consumption severity: addiction vs. long-term use vs. occasional use; (3) type of substance: alcohol vs. tobacco vs cannabis vs. cocaine vs. stimulants vs. opioids vs. ketamine, and papers that pooled together substances which we termed polysubstance; and (4) associated comorbidities: single vs. multiple. Age and comorbidity subgroups resulted in insufficient experiments (foci) to conduct an ALE analysis (<15). However, we found significant ALE maps in the subgroups consumption severity and type of substance (Fig. 2).

#### Subgroup analysis by type consumption

The first subgroup meta-analysis reported ALE maps of substance users (SU) against healthy controls (HC), by type of consumption severity (addiction vs. long-term use vs. occasional use). We found significant ALE maps showing lower GM and WM volumes across all types of consumption. Additionally, higher GM volumes were also shown across all types of consumption, and higher WM only in long-term use (Fig. 2 and Supplementary Table 3).

**Fig. 3:**
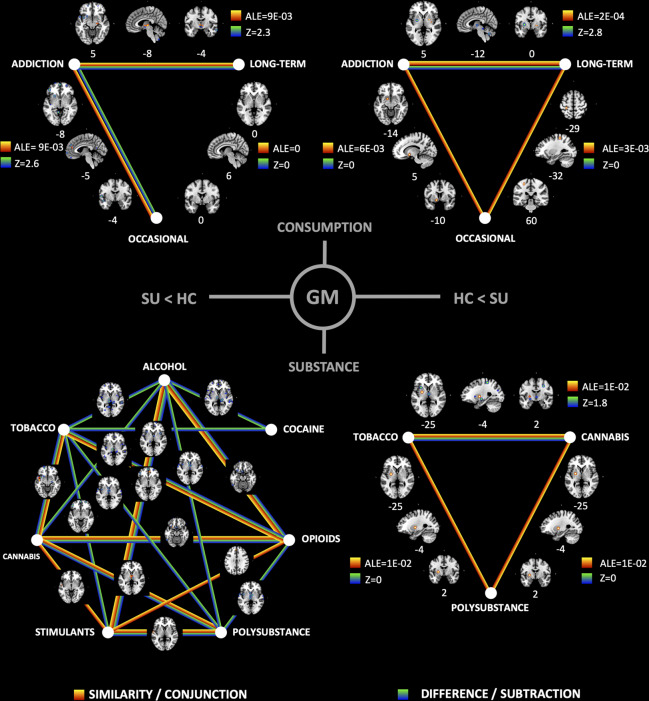
Contrast analyses of subgroup anatomic likelihood estimation meta-analytic results for studies comparing brain morphological changes between SU and HC, at cluster level inference *p* < 0.05 (FWE). Contrast analyses were performed for consumption severity (top) and type of substance (bottom) subgroups. Subgroups were tested for similarity (conjunction) and difference (subtraction) in a contrast analysis, to illustrate common and/or distinct areas between the elements of each subgroup analysis. ALE anatomic likelihood estimation value; *Z* peak *Z*-value.

We conducted contrast analyses between the ALE maps of each subgroup, to determine similarity (conjunction) and/or difference (subtraction) of affected brain regions between the types of consumption (Fig. 3 and Supplementary Table 4). Addiction and long-term use were both associated with lower GM volume of the thalamus but differ in terms of lower GM of red nucleus, substantia nigra, and putamen. These results support the idea that the thalamus is affected across all levels of SUD severity, and future research should focus on the correlation between SUD progression and the volume/form of the thalamus, as its morphology may predict severity of the disease, and/or monitor the efficacy of treatments and therapies. Addiction and occasional use both show higher volume of the globus pallidus, while differ in lower volume of fronto-temporal areas including the medial frontal gyrus, anterior cingulate cortex and superior temporal gyrus, supporting cortical alterations in occasional use. Finally, long-term use and occasional use share higher volume of somatomotor cortices, due to possible drug intoxication. In terms of WM, addiction and long-term use share lower volume of the anterior thalamic radiations and the corpus callosum, suggesting also a probable correlation between the progression of SUD and the severity in WM structural alteration.

#### Subgroup analysis by type of substance

In the second subgroup meta-analysis, we reported ALE maps of substance users (SU) against healthy controls (HC) by type of substance. Given that we only included one publication on ketamine, this substance was not included in the subgroup analysis. We found significant ALE maps showing lower GM volume in all substances, and higher GM volume only in tobacco, cannabis, and polysubstance. Also, we found lower WM volume in alcohol, tobacco and cocaine, and found no higher WM volume in any substance (Fig. 2 and Supplementary Table 5).

We conducted contrast analyses between the ALE maps of each subgroup, to determine similarity (conjunction) and/or difference (subtraction) of affected brain regions between the types of substance (Fig. 3 and Supplementary Table 6). Alcohol, overall, differed with most of the other substances including tobacco, cocaine, cannabis, and opioids. Conversely, cannabis shared affected areas with tobacco, opioids, stimulants, and polysubstance. Consistent affected shared areas included thalamus, insula, inferior frontal gyrus, and superior temporal gyrus in GM; and anterior thalamic radiation in WM. Although most addictive substances share a common neurobiological process in the reward circuitry, it is evident that neuroadaptations in SUD depend on the type of substance used. Results of this subgroup analysis by substance is valuable for future research into the best approach for therapeutics (pharmacological and behavioral), as treatment effects can be correlated with brain morphometry.

## Discussion

In this systematic review and meta-analysis, we used coordinate-based anatomic likelihood estimation (ALE) to pool the effects of substance use disorders (SUDs) on brain regional volume. We found that the most converging regions with volume pathology in SUDs were putamen, thalamus, insula and anterior cingulate cortex in gray matter (GM), and the thalamic radiation, corticospinal tract, and corpus callosum in white matter (WM). We found that consumption severity and type of substance subgroups resulted in significant ALE maps with both shared and distinctive regions involved, supporting converging and divergent effects depending on severity and type of substance use.

### Characteristics of the included studies

Overall, the included publications clearly stated their research question, population, inclusion and exclusion criteria, measurements, and outcomes. We found that most of the publications failed to report the type of evaluator (e.g., psychiatrist), and some did not mention if the DSM or other diagnostic criteria were used to diagnose SUD. In terms of MRI characteristics and quality of the studies, we found that all included studies used state-of-the-art techniques and statistical tools, and therefore support the standardization of neuroimaging studies as a key element in future research and reproducibility efforts^31–33^. However, a larger effort is needed to provide diagnosis criteria, which would result in improved classifications for future reviews and meta-analyses.

### Primary outcome: altered brain morphometry in SUDs

SUDs seems to disrupt the normal function of the limbic loop of the basal ganglia^3^. Neuroplastic adaptations in cortical and subcortical regions seem to progress with the severity of the SUD^46^. However, the relation between repeated dopaminergic signaling in the basal ganglia and volumetric alterations is still unclear, thus, causality should be interpreted with caution. Indeed, we report consistent volumetric alterations in putamen, thalamus, insula, and anterior cingulate cortex in GM, and internal capsule and thalamic radiations in WM, supporting the idea that the entire limbic loop of the basal ganglia shows neuroadaptations associated to SUDs.

Higher putamen volume may be explained by the repeated glutamatergic spikes onto dopamine neurons (VTA/SNc) and into MSN in dorsal and ventral striatum, supported by behavioral changes in reward responsivity and habituation that characterize SUDs. Notably, almost all regions of the neocortex project direct input to the striatum. Most of these projections come from association areas in frontal and parietal lobes, with contributions from temporal, insular, and cingulate cortices. These projections (corticostriatal pathway) travel via the internal capsule to reach the caudate and putamen^47^. We also found higher WM volume of the internal capsule in SUDs, suggesting neuroadaptive processes in this pathway as well. It has been suggested that SUDs or addiction are a disease of self-control^48^. Although the study of SUDs has been focused mainly on the role of dopamine and the reward system, new findings of clinical studies have revealed neuroplastic mechanisms in frontocortical regions that may underlie reward-seeking behavior^13^. In susceptible individuals, certain stimuli may activate strong urges that are not congruent with a given context. The lack of a proper inhibitory control may keep these urges in control up to a point, when stronger impulses and deficient inhibition result in impulsive or compulsive behavior^49^. Current models of SUDs suggest that impulsivity and compulsivity characterize the pathological behavior and help explain our structural results^3^.

It has been proposed that the insula and the anterior cingulate cortex form the salience network (SN), that coordinates between the default mode network (DMN) and the central executive network (CEN)^50^. In our study we found lower volume of the insula, a region whose morphology has been associated with substance use compulsion and severity^51^. The insula plays a major role in interoception by integrating information from the internal physiological state, and projecting information to the ACC, ventral striatum and prefrontal cortex to initiate adaptive responses^52^. In SUDs, the insula’s ability to switch between networks seems to be affected, as well as its functional connectivity with the ACC, amygdala and putamen^53–55^. Similarly, SUD neuroimaging studies have shown disrupted activity of the ACC^3^, involved in inhibitory control^56^, and altered connectivity with the insula^53^. The rostral part of the ACC is implicated in error-related responses, including affective processing, and the caudal part of the ACC is associated with detection of conflict to recruit cognitive control^57^. Thus, reduction in inputs from prefrontal and cingulate cortices into striatum may disrupt the control over action selection^58^ (see Supplementary Table 7 for MACM, and Supplementary Table 8 for functional characterization).

Finally, we found that SUDs were associated with lower thalamic GM/WM across several substances including alcohol, cocaine, nicotine, methamphetamine, opioids, and cannabis^59,60^. Reduced structural and functional integrity of the thalamus and its connectivity appear to be associated with the severity of SUD^61^. Overall, there are brain regions consistently affected in all SUDs, with diverging MRI manifestations (higher vs. lower volume) suggesting different underlying structural pathology between brain regions.

### Common and distinct patterns of brain volume alterations across consumption severity

The effect of substance use in the brain seems to vary across the severity of consumption. Cortical structures seem affected in occasional use, while established addictive consumption (addiction) seems to also affect subcortical regions of the brain such as thalamus and basal ganglia. Such disrupted GM areas may presumably be co-affected with its respective WM thalamic radiation and corpus callosum connection, as seen in our results. Occasional use seems to affect WM tracts of the cingulum, connecting the limbic system with areas such as the cingulate gyrus, entorhinal cortex, and temporal lobe. Neuroimaging studies have found that disruption of the posterior cingulum is associated to cognitive impairment^62^. The forceps minor connects the lateral and medial surfaces of the frontal lobes and crosses the midline via the genu of the corpus callosum^47^, and also showed structural alterations in occasional use. Along with the anterior thalamic radiation, the forceps minor connects ACC and striatum to the anterior frontal regions, modulating executive functions^63^.

Various physiological mechanisms such as oxidative stress, mitochondrial dysfunction, or neurotrophic factor dysfunction might account for the observed cortical GM volume reductions in occasional use^64^. Presumably, repeated dopaminergic stimulation from substance abuse produce neuroadaptations (e.g., dendritic morphology and ionotropic glutamate receptors), that result in long-term potentiation (LTP) and long-term depression (LTD)^11^ of the basal ganglia neurocircuitry^3^. These results suggest that cortical morphological pathology in SUDs appears before subcortical pathology or that subcortical pathology is only seen when addiction is established. This needs to be explored further with longitudinal studies.

### Common and distinct patterns of brain volume alterations across types of substances

Reward processes are shared between substances, namely repeated stimulation into the VTA which releases dopamine into the ventral striatum^3^. However, the stimulation of the mesolimbic system depends on the different molecular targets for each kind of substance. For example, alcohol, unlike most other drugs, affects a wide range of targets and indirectly increases dopamine in the NAc^65^. Stimulants like amphetamine and cocaine block dopamine transporters, thus increasing dopamine in NAc^66^. Cannabis activates receptors that release neurotransmitters (GABA/Glutamate), modulating the activity of the mesolimbic system. Opioids, agonists of mu opioid receptors (MOR) in VTA, increase striatal dopamine release^67^. Nicotine and its interactions with nicotinic acetylcholine receptors, increases neuronal activity in VTA^68^. In our results, most of the substances show a convergent effect and region, namely lower volume of the thalamus.

Divergently, alcohol seems to affect frontal areas including superior and medial frontal gyrus, as well as ACC. Tobacco use shows a myriad of alterations including lower volume in insula and posterior areas of the DMN, such as PCC and precuneus. Cocaine users show lower volume of the claustrum, a structure that connects prefrontal areas with the thalamus, and has close proximity to the insula and putamen^69^. Cannabis use reduces the volume of temporal areas and thalamus, and increases the volume of putamen, while opioid use affects cortical fronto-temporal areas. Stimulant use mainly reduces GM volume of the frontal lobe. Polysubstance studies, as expected, show a wide variety of affected areas including lower volume of the anterior cingulate gyrus, thalamus, and superior temporal gyrus, and show higher volume of the subcallosal gyrus. In terms of WM, the affected convergent regions were the corticospinal tract, anterior thalamic radiation, the corpus callosum, and the cingulum. Overall, different substances show convergent and divergent morphological pathology, suggesting different physiopathology and possibly therapeutic approaches in SUDs that need to be considered.

### Limitations and future perspectives

A comprehensive review of the current kind is valuable in both synthesizing the effect of SUD on brain morphometry and highlighting issues in the field for future perspectives. For example, by setting a clear contrast based on MRI paradigms (e.g., SU < HC), we try to narrow the heterogeneity inherent to SUD as we relied on two assumptions: (1) contrasts we pool are based on best practices and (2) the Ginger ALE method. To conduct the ALE meta-analysis, we pooled peak coordinates derived from the included studies, rather the original raw structural MRI images. The accuracy of our findings relies on the result of a statistical estimation of coordinate-based anatomic foci (input), treated as spatial probability distributions centered at the given coordinates. Consequently, the individual profiles of substance users are not included in this review, and future syntheses might examine individual participant-level data, as these become increasingly available. For example, a number of studies may investigate a primary substance, but individuals within such studies may consume other substances as well, and are not listed as polysubstance users.

Notably, we included only VBM studies, and recognize that other methods measuring brain structure may provide more accurate morphometric results. However, a whole-brain approach is an important requisite in coordinate-based meta-analyses^70^, in which anatomic convergence across experiments is tested under the assumption that each voxel has a priori the same chance of being significant. Thus, inclusion of heterogeneous analyses such as region-of-interest (ROIs) or small volume corrected (SVC) analyses would violate such assumption and lead to overrepresentation of those regions. Likewise, VBM studies on WM are not as precise as, for example, diffusion-weighted imaging (DWI).

The heterogeneity among the methods used in the included studies, such as preprocessing software, smoothing, statistical thresholds, participants’ characteristics, medication history and comorbidity, represent additional confounders. Meta-regression analysis is not compatible with GingerALE. We therefore did not perform regression-based assessments of factors that might be implicated in heterogeneity (e.g., age of participants, age of first use, and total years of SUD).

As traditional meta-analyses, coordinate-based meta-analyses such as ALE can be subject to different forms of publication bias which may impact results and invalidate findings (e.g., the “file drawer problem”). We performed the Fail-Safe N analysis (FSN)^43^ as a measure of robustness against potential publication bias. It is estimated for normal human brain mapping that a 95% confidence interval for the number of studies that report no local maxima varies from 5 to 30 per 100 published studies. In our study, we tested 11 clusters resulting from our primary outcomes. We found that all clusters showed an FNR greater or equal than the minimum imposed of 18. FNR was >350 for clusters resulting from all GM foci analysis, and >300 for clusters resulting from all WM foci analysis. Thus, indicating a robust convergence of foci in these regions but also indicating that proportionally fewer studies are needed to obtain this effect. Two clusters from the comparison GM SU < HC showed an FSN between the lower and upper boundary (Supplementary Table 9).

In our review, the included studies did not acquire the long-term measurements necessary to show that SUD is temporally linked to a decrease or increase of brain tissue, as a longitudinal design might provide; but they rather examine brain morphometry in established substance users compared to non-users. Socio-economic and educational background data on participants are lacking in most of the studies, limiting the potential for statistical correction using naturalistic environmental confounders.

We conducted a sensitivity analysis on primary outcomes, excluding 1.5 T studies (Supplementary Table 10). Potentially, studies using different field strength scanners may result in varying levels of signal-to-noise and contrast-to-noise ratios. As the ALE method relies only on coordinates reported on the included studies, the fields strength is not considered and may affect the results. No differences with the main results were identified.

In the consumption and substance subgroup analyses, the number of experiments for each category of the subgroup analyses was unmatched (e.g., addiction 64%, occasional use 29%, and long-term use 7%). Although the ALE method weights the result on the number of participants per experiment, the resulting ALE maps of subgroup and contrast analyses should be interpreted with caution.

The progression from initial drug use to established SUD may depend on age and developmental stage^71^. Critical periods of development are characterized by functional neuroplastic mechanisms that may be easily altered by pathological neuroadaptations due to SUD^5^. For example, delays in maturation associated with drug exposure, genetics, or social environment, may increase risky behaviors in adolescents^72^. Brain imaging studies have found altered structure of prefrontal cortices associated with higher risk for SUD in adolescents^73^, suggesting that control executive functions such as decision making and impulse control (inhibition) are immature^74^. Unfortunately, the neurobiological underpinnings of neuroadaptations for both functional development and SUD, are not fully understood, in part, by a high variability in VBM results^75^. In this review, the included studies failed to report enough experiments (foci < 15), to conduct an age subgroup analysis (e.g., adolescents vs. adults).

SUDs are frequently co-diagnosed with psychiatric and neurological disorders (Common comorbidities with substance use disorders). Research suggests that adolescents with SUD have high rates of co-occurring mental illness, up to 60%^76^. The most common psychiatric comorbidities with SUD include anxiety disorders, post-traumatic stress disorder, depression, bipolar disorder, attention-deficit hyperactivity disorder, psychosis, borderline disorder, and schizophrenia. Notably, establishing causality or directionality between mental illness and SUD is difficult, however, common risk factors are shared^77^. Additionally, recent research has focused on the neurological effects of SUD, rather than as comorbid, co-occurring alterations^78^ (e.g., SUD and Parkinson’s disease). In this review, the included studies failed to report enough experiments (foci < 15), to conduct a comorbidity subgroup analysis (e.g., pure addiction vs. comorbid addiction). Nevertheless, it is important to recognize that mental illness and SUD share alterations in the same neurotransmitter systems (e.g., dopaminergic^4^) and in brain areas involved in reward, decision making, impulse control, and emotion^79^.

## Conclusions

In conclusion, the present systematic review and meta-analysis of voxel-based morphometry neuroimaging studies provides evidence of common and distinct morphological gray matter and white matter pathology in substance use disorders. We found consistent morphometric alterations in regions of the insula, anterior cingulate cortex, basal ganglia (putamen), and thalamus, with their respective white matter thalamic radiation and internal capsule bundle. Our subgroup analysis showed distinct volume alterations depending on the type of consumption (occasional vs. long-term vs. addiction) and type of substance. This evidence may help future studies to better understand substance use disorders and possible new therapeutic approaches.

## Supplementary information

Supplementary Information

## References

[1] Association, A. P. *Diagnostic and Statistical Manual of Mental Disorders* 5th edn (American Psychiatric Association, Washington, 2013).

[2] United Nations Office on Drugs and Crime. World Drug Report 2019. (UN, 2019). 10.18356/a4dd519a-en.

[3] Koob, G. F. & Volkow, N. D. Neurocircuitry of addiction. Neuropsychopharmacology 35, 217–238 (2009). 10.1038/npp.2009.110 PMC2805560 19710631

[4] Volkow, N. D., Fowler, J. S., Wang, G. J., Baler, R. & Telang, F. Imaging dopamine’s role in drug abuse and addiction. *Neuropsychopharmacology*10.1016/j.neuropharm.2008.05.022 (2009). 10.1016/j.neuropharm.2008.05.022 PMC2696819 18617195

[5] Volkow ND, Michaelides M, Baler R (2019). The neuroscience of drug reward and addiction. Physiol. Rev..

[6] Crow TJ (1973). Catecholamine-containing neurones and electrical self-stimulation: 2. A theoretical interpretation and some psychiatric implications. Psychol. Med..

[7] Everitt BJ, Robbins TW (2016). Drug addiction: updating actions to habits to compulsions ten years on. Annu. Rev. Psychol..

[8] Vink, J. M. Genetics of addiction: future focus on gene × environment interaction? *J. Stud. Alcohol Drugs*10.1037/a0017376 (2016). 10.15288/jsad.2016.77.684 27588524

[9] Mitchell, M. R., Berridge, K. C. & Mahler, S. V. Endocannabinoid-enhanced ‘“Liking”’ in nucleus accumbens shell hedonic hotspot requires endogenous opioid signals. *Cannabis Cannabinoid Res.*10.1089/can.2018.0021 (2018). 10.1089/can.2018.0021 PMC6069591 30069500

[10] Grueter BA, Rothwell PE, Malenka RC, Sheng M, Triller A (2012). Integrating synaptic plasticity and striatal circuit function in addiction: this review comes from a themed issue on Synaptic structure and function Edited. Curr. Opin. Neurobiol..

[11] Kauer JA, Malenka RC (2007). Synaptic plasticity and addiction. Nat. Rev. Neurosci..

[12] Fowler JS, Volkow ND, Kassed CA, Chang L (2007). Imaging the addicted human brain. Science & practice perspectives/a publication of the National Institute on Drug Abuse. Natl Inst. Health.

[13] Goldstein, R. Z. & Volkow, N. D. Drug addiction and its underlying neurobiological basis: neuroimaging evidence for the involvement of the frontal cortex NIH public access. *Am. J. Psychiatry* 159, 1642–1652 (2002). 10.1176/appi.ajp.159.10.1642 PMC1201373 12359667

[14] Demirakca, T. et al. Effects of alcoholism and continued abstinence on brain volumes in both genders. *Alcoholism* **35**, 1678–1685 (2011). 10.1111/j.1530-0277.2011.01514.x 21599718

[15] Khn S. et al. Brain grey matter deficits in smokers: focus on the cerebellum. Brain Struct. Funct. 217, 517–522 (2012). 10.1007/s00429-011-0346-5 21909705

[16] Almeida OP (2008). Smoking is associated with reduced cortical regional gray matter density in brain regions associated with incipient Alzheimer disease. Am. J. Geriatr. Psychiatry.

[17] Battistella, G. et al. Long-term effects of Cannabis on brain structure giovanni. *Neuropsychopharmacology* **39**, 2041–2048 (2014). 10.1038/npp.2014.67 PMC4104335 24633558

[18] Matochik JA, Eldreth DA, Cadet J-L, Bolla KI (2005). Altered brain tissue composition in heavy marijuana users. Drug Alcohol Depend.

[19] Matochik, J. A., London, E. D., Eldreth, D. A., Cadet, J.-L. & Bolla, K. I. Frontal cortical tissue composition in abstinent cocaine abusers: a magnetic resonance imaging study. *Neuroimage* **19**, 1095–1102 (2003). 10.1016/s1053-8119(03)00244-1 12880835

[20] Gallinat J (2006). Smoking and structural brain deficits: a volumetric MR investigation. Eur. J. Neurosci..

[21] Sim ME (2007). Cerebellar gray matter volume correlates with duration of cocaine use in cocaine-dependent subjects. Neuropsychopharmacology.

[22] Bu, L. et al. Functional connectivity abnormalities of brain regions with structural deficits in young adult male smokers. *Front. Hum. Neurosci.*10.3389/fnhum.2016.00494 (2016). 10.3389/fnhum.2016.00494 PMC5047919 27757078

[23] Wetherill, R. R. et al. Cannabis, cigarettes, and their co-occurring use: disentangling differences in gray matter volume. *Int. J. Neuropsychopharmacol.*10.1093/ijnp/pyv061 (2015). 10.1093/ijnp/pyv061 PMC4648161 26045474

[24] Aoki Y (2013). Volume reductions in frontopolar and left perisylvian cortices in methamphetamine induced psychosis. Schizophr. Res..

[25] Chanraud, S. et al. Brain morphometry and cognitive performance in detoxified alcohol-dependents with preserved psychosocial functioning. *Neuropsychopharmacology*10.1038/sj.npp.1301219 (2007). 10.1038/sj.npp.1301219 17047671

[26] Jang, D.-P. et al. The relationship between brain morphometry and neuropsychological performance in alcohol dependence. *Neurosci. Lett.* **428**, 21–26 (2007). 10.1016/j.neulet.2007.09.047 17951002

[27] Gipson CD, Kupchik YM, Kalivas PW (2014). Rapid, transient synaptic plasticity in addiction. Neuropharmacology.

[28] Higgins, J. P. & Green, S. *Cochrane Handbook for Systematic Reviews of Interventions*. (Wiley, Hoboken, 2011).

[29] Moher D, Liberati A, Tetzlaff J, Altman DG, Group TP (2009). Preferred reporting items for systematic reviews and meta-analyses: the PRISMA statement. PLoS Med..

[30] *Covidence Systematic Review Software* (Veritas Health Innovation, Melbourne). https://support.covidence.org/help/how-can-i-cite-covidence.

[31] Poldrack RA (2008). Guidelines for reporting an fMRI study. Neuroimage.

[32] Tahmasian M (2019). Practical recommendations to conduct a neuroimaging meta-analysis for neuropsychiatric disorders. Hum. Brain Mapp..

[33] Nichols TE (2017). Best practices in data analysis and sharing in neuroimaging using MRI. Nat. Neurosci..

[34] Eickhoff SB, Bzdok D, Laird AR, Kurth F, Fox PT (2012). Activation likelihood estimation meta-analysis revisited. Neuroimage.

[35] Laird, A. R. et al. Comparison of the disparity between Talairach and MNI coordinates in functional neuroimaging data: validation of the Lancaster transform. *Neuroimage*10.1016/j.neuroimage.2010.02.048 (2010). 10.1016/j.neuroimage.2010.02.048 PMC2856713 20197097

[36] Eickhoff, S. B. et al. Coordinate-based activation likelihood estimation meta-analysis of neuroimaging data: a random-effects approach based on empirical estimates of spatial uncertainty. *Hum. Brain Mapp.*10.1002/hbm.20718 (2009). 10.1002/hbm.20718 PMC2872071 19172646

[37] Fox, P. T. et al. *User Manual for GingerALE 2.3* (Research Imaging Institute, UT Health Science Center, San Antonio, 2013).

[38] Robinson JL, Laird AR, Glahn DC, Lovallo WR, Fox PT (2010). Metaanalytic connectivity modeling: delineating the functional connectivity of the human amygdala. Hum. Brain Mapp..

[39] Laird AR (2013). Networks of task co-activations. Neuroimage.

[40] Laird, A. R. et al. Investigating the Functional Heterogeneity of the Default Mode Network Using Coordinate-Based Meta-Analytic Modeling. 10.1523/JNEUROSCI.4004-09.2009 (2019). 10.1523/JNEUROSCI.4004-09.2009 PMC2820256 19923283

[41] Gorgolewski KJ (2015). NeuroVault.org: a web-based repository for collecting and sharing unthresholded statistical maps of the human brain. Front. Neuroinform..

[42] Jenkinson M, Beckmann CF, Behrens TEJ, Woolrich MW, Smith SM (2012). FSL. Neuroimage.

[43] Acar F, Seurinck R, Eickhoff SB, Moerkerke B (2018). Assessing robustness against potential publication bias in activation likelihood estimation (ALE) meta-analyses for fMRI. PLoS ONE.

[44] Friston K (1995). Statistical parametric maps in functional imaging: a general linear approach. Hum. Brain Mapp..

[45] Cox RW (1996). AFNI: software for analysis and visualization of functional magnetic resonance neuroimages. Comput. Biomed. Res..

[46] Morales M, Pickel VM (2012). Insights to drug addiction derived from ultrastructural views of the mesocorticolimbic system. Ann. N. Y. Acad. Sci..

[47] Haber, Suzanne N. “Corticostriatal Circuitry.” *Dialogues Clin. Neurosci.* **18**, 7–21 (2016). 10.31887/DCNS.2016.18.1/shaber PMC4826773 27069376

[48] Volkow, N. D. & Baler, R. Addiction: a disease of self-control. *Neurosci. Hum. Person* 1–6 (2013).

[49] Dalley JW, Everitt BJ, Robbins TW (2011). Impulsivity, compulsivity, and top-down cognitive control. Neuron.

[50] Menon V, Uddin LQ (2010). Saliency, switching, attention and control: a network model of insula function. Brain Struct. Funct..

[51] Betka S (2019). Signatures of alcohol use in the structure and neurochemistry of insular cortex: a correlational study. Psychopharmacology.

[52] Paulus MP, Tapert SF, Schulteis G (2009). The role of interoception and alliesthesia in addiction. Pharmacol. Biochem. Behav..

[53] Droutman V, Read SJ, Bechara A (2015). Revisiting the role of the insula in addiction. Trends Cogn. Sci..

[54] Naqvi, N. H. & Bechara, A. The hidden island of addiction: the insula. *Trends Neurosci.*10.1016/j.tins.2008.09.009 (2009). 10.1016/j.tins.2008.09.009 PMC3698860 18986715

[55] Liang X (2015). Interactions between the salience and default-mode networks are disrupted in cocaine addiction. J. Neurosci..

[56] Volkow ND, Fowler JS, Wang G-J (2004). The addicted human brain viewed in the light of imaging studies: brain circuits and treatment strategies. Neuropharmacology.

[57] Tang YY, Posner MI, Rothbart MK, Volkow ND (2015). Circuitry of self-control and its role in reducing addiction. Trends Cogn. Sci..

[58] Renteria R, Baltz ET, Gremel CM (2018). Chronic alcohol exposure disrupts top-down control over basal ganglia action selection to produce habits. Nat. Commun..

[59] Nurmedov, S. et al. Thalamic and cerebellar gray matter volume reduction in synthetic cannabinoids users. *Eur. Addict. Res.* **21**, 315–320 (2015). 10.1159/000430437 26021304

[60] Wilson, B. A., Winegardner, J., Heugten, C. M. V. & Ownsworth, T. (Eds.). *Neuropsychological Rehabilitation: The International Handbook*, 1st edn. (Routledge, 2017) 10.4324/9781315629537.

[61] Garza-Villarreal EA (2017). The effect of crack cocaine addiction and age on the microstructure and morphology of the human striatum and thalamus using shape analysis and fast diffusion kurtosis imaging. Transl. Psychiatry.

[62] Bubb EJ, Metzler-Baddeley C, Aggleton JP (2018). The cingulum bundle: anatomy, function, and dysfunction. Neurosci. Biobehav. Rev..

[63] Mamiya, P. C., Richards, T. L. & Kuhl, P. K. Right forceps minor and anterior thalamic radiation predict executive function skills in young bilingual adults. *Front. Psychol.* **9**, 118 (2018). 10.3389/fpsyg.2018.00118 PMC5811666 29479331

[64] Yamamoto BK, Moszczynska A, Gudelsky GA (2010). Amphetamine toxicities. Ann. N. Y. Acad. Sci..

[65] Wiers CE, Cabrera E, Skarda E, Volkow ND, Wang GJ (2016). PET imaging for addiction medicine: from neural mechanisms to clinical considerations. Prog. Brain Res..

[66] Kahlig KM, Galli A (2003). Regulation of dopamine transporter function and plasma membrane expression by dopamine, amphetamine, and cocaine. Eur. J. Pharmacol..

[67] Macey TA, Lowe JD, Chavkin C (2006). Mu opioid receptor activation of ERK1/2 is GRK3 and arrestin dependent in striatal neurons. J. Biol. Chem..

[68] Bertrand D, Terry AV (2018). The wonderland of neuronal nicotinic acetylcholine receptors. Biochem. Pharmacol..

[69] Chau A, Salazar AM, Krueger F, Cristofori I, Grafman J (2015). The effect of claustrum lesions on human consciousness and recovery of function. Conscious. Cogn..

[70] Müller VI (2018). Ten simple rules for neuroimaging meta-analysis. Neurosci. Biobehav. Rev..

[71] Glisky, E. L. Changes in cognitive function in human aging. *Brain Aging*10.1201/9781420005523-1 (2019).

[72] Guerri, C. & Pascual, M. Mechanisms involved in the neurotoxic, cognitive, and neurobehavioral effects of alcohol consumption during adolescence. 10.1016/j.alcohol.2009.10.003 (2010). 10.1016/j.alcohol.2009.10.003 20113871

[73] Jones SA, Morales AM, Lavine JB, Nagel BJ (2017). Convergent neurobiological predictors of emergent psychopathology during adolescence. Birth Defects Res..

[74] Winters, K. C., Tanner-Smith, E. E., Bresani, E. & Meyers, K. Current advances in the treatment of adolescent drug use. *Adolesc. Health. Med. Ther.*10.2147/AHMT.S48053 (2014). 10.2147/AHMT.S48053 PMC4241949 25429247

[75] Gogtay N, Thompson PM (2010). Mapping gray matter development: implications for typical development and vulnerability to psychopathology. Brain Cogn..

[76] Hser YI (2001). An evaluation of drug treatments for adolescents in 4 US cities. Arch. Gen. Psychiatry.

[77] Cerdá M, Sagdeo A, Johnson J, Galea S (2010). Genetic and environmental influences on psychiatric comorbidity: a systematic review. J. Affect. Disord..

[78] McHugo GJ (2017). The prevalence of traumatic brain injury among people with co-occurring mental health and substance use disorders. J. Head. Trauma Rehabil..

[79] Wing VC, Wass CE, Soh DW, George TP (2012). A review of neurobiological vulnerability factors and treatment implications for comorbid tobacco dependence in schizophrenia. Ann. N. Y. Acad. Sci..

