# Supplementary material for: Gray and white matter morphology in substance use disorders: a neuroimaging systematic review and meta-analysis
Source: Transl Psychiatry. 2021 Jan 11;11:29. doi: 10.1038/s41398-020-01128-2 (PMC7801701; doi:10.1038/s41398-020-01128-2)
Supplement: Supplementary file 1 — Supplementary Information [file 41398_2020_1128_MOESM1_ESM.docx]

**SUPPLEMENTARY INFORMATION**

**Gray and white matter morphology in substance use disorders: A neuroimaging systematic review and meta-analysis.**

**METHODS**

**Primary literature search and selection.**

This systematic review and meta-analysis followed procedures from the Cochrane Handbook for Systematic Reviews^1^, and from the Center for Reviews and Dissemination (<https://www.york.ac.uk/crd/>). The review protocol was pre-registered in PROSPERO ([CRD42017071222](http://www.crd.york.ac.uk/PROSPERO/display_record.php?ID=CRD42017071222)). The PRISMA statement was used to address relevant items in this systematic review and meta-analysis^2^.

**Search strategy.**

We conducted a systematic literature search in PubMed, Scopus and PsycInfo, using both keywords and MeSH terms for articles published up to August 10^th^, 2020. Keyword terms and MeSH terms included: substance-related disorders, alcohol-related disorders, amphetamine-related disorders, cocaine-related disorders, inhalant abuse, marijuana abuse, substance abuse (intravenous), tobacco use disorder, drug utilization, cannabis, cocaine, crack cocaine, alcoholics, amphetamine, methamphetamine, N-Methyl-3,4-methylenedioxyamphetamine. No restrictions were placed on study design, but in order to be eligible for inclusion, the studies must have used VBM analyses (search terms in below).

**Study eligibility.**

Studies were included if they met the following criteria: (1) an original report of whole-brain VBM analyses, (2) the study population included substance users above 18 years of age, with a continuous level of usage (at least once a month in the last 6 months), (3) studies included a substance user group and a non-exposed group (healthy control group), and (4) results were reported in stereotactic coordinates either Talairach or Montreal Neurological Institute (MNI) three-dimensional-coordinate system. If any of these data points were not reported in the paper, we contacted authors to retrieve this information. We contacted a total of 4 authors, with no response.

Studies were excluded using the following criteria: (1) review articles with no original experimental data, (2) neuroimaging data from non-MRI studies (e.g. PET), (3) the type of addiction was gambling, internet, etc. (non-substance addiction), and (4) the VBM analyses were region-of-interest (ROI)-based and not whole-brain-based.

Two reviewers (VP and ST) independently screened by title and abstract and selected articles for full-text review, and also performed full-text reviews. Screening and data extraction were performed using the Covidence tool^3^. Any disagreements that arose between the reviewers were resolved through discussion or by a third and/or fourth reviewer (SA / EGV). A total of 29 disagreements were resolved in the title and abstract screening, and 21 disagreements were resolved during full-text screening. A total of 60 studies fulfilled criteria and were therefore included for data extraction.

**Data extraction.**

From each study, the following variables were extracted: first author, year of publication, population of interest, use of the Diagnostic and Statistical Manual of Mental Disorders (DSM), type of addiction (pure or dual = comorbidity), number of participants, sex, age, type of substance, type of consumption, years of consumption, age of onset of consumption, MRI-system, MRI-model, Head coil, image acquisition, T1w sequence, voxel size and analysis software. The main outcome to extract was any change in gray and/or white matter analyzed using VBM, in stereotactic coordinates, comparing a substance user group and a healthy control group (SU vs HC).

**Search strings**

Last search date: 10.08.2020

**PsycInfo**

(voxel-based morphometry OR voxel based morphometry OR vbm) AND (addiction OR substance-related disorders OR alcohol-related disorders OR amphetamine-related disorders OR cocaine-related disorders OR inhalant abuse OR marijuana abuse OR substance abuse intravenous OR tobacco use disorder OR drug utilization OR drug abuse OR drug dependency OR substance utilization OR substance abuse OR substance dependency OR cannabis OR marihuana OR marijuana OR tetrahydrocannabinol OR thc OR cocaine OR crack cocaine OR alcohol OR alcoholics OR amphetamine OR methamphetamine OR n-methyl-3,4-methylenedioxyamphetamine OR mdma OR ecstasy OR heroin OR nicotine OR tobacco)

302 results

**Scopus**

( ( TITLE-ABS-KEY ( **voxel-based morphometry**)  OR  TITLE-ABS-KEY ( **voxel based morphometry**) OR  TITLE-ABS-KEY ( **vbm**) )  AND  ( TITLE-ABS-KEY ( **addiction**) OR  TITLE-ABS-KEY ( **substance-related disorders**)  OR  TITLE-ABS-KEY ( **alcohol-related disorders**)  OR  TITLE-ABS-KEY ( **amphetamine-related disorders**)  OR  TITLE-ABS-KEY ( **cocaine-related disorders**)  OR  TITLE-ABS-KEY ( **inhalant abuse**)  OR  TITLE-ABS-KEY ( **marijuana abuse**)  OR  TITLE-ABS-KEY ( **substance abuse intravenous**)  OR  TITLE-ABS-KEY ( **tobacco use disorder**)  OR  TITLE-ABS-KEY ( **drug utilization**)   OR  TITLE-ABS-KEY ( **drug abuse**)  OR  TITLE-ABS-KEY ( **drug dependency**)  OR  TITLE-ABS-KEY ( **substance utilization**)  OR  TITLE-ABS-KEY ( **substance abuse**)  OR  TITLE-ABS-KEY ( **substance dependency**)  OR  TITLE-ABS-KEY ( **cannabis**)  OR  TITLE-ABS-KEY ( **marihuana**)  OR  TITLE-ABS-KEY ( **marijuana**)  OR  TITLE-ABS-KEY ( **tetrahydrocannabinol**)  OR  TITLE-ABS-KEY ( **thc**)  OR  TITLE-ABS-KEY ( **cocaine**) OR  TITLE-ABS-KEY ( **crack cocaine**) OR  TITLE-ABS-KEY ( **alcohol**) OR  TITLE-ABS-KEY ( **alcoholics**) OR  TITLE-ABS-KEY ( **amphetamine**) OR  TITLE-ABS-KEY ( **methamphetamine**) OR  TITLE-ABS-KEY ( **n-methyl-3,4-methylenedioxyamphetamine**) OR  TITLE-ABS-KEY ( **MDMA**) OR  TITLE-ABS-KEY ( **ecstasy**) OR  TITLE-ABS-KEY ( **heroin**) OR  TITLE-ABS-KEY ( **nicotine**) OR  TITLE-ABS-KEY ( **tobacco**) ) )

471 results

**PubMed**

(((((((“voxel-based morphometry”[All Fields]) ((“voxel-based”[All Fields]) AND (“morphometry”[All Fields])) OR (“voxel based morphometry”[All Fields]) OR ((“voxel”[All Fields]) AND (“based”[All Fields]) AND (“morphometry”[All Fields])) OR (“vbm”[All Fields])))) AND ((((“addiction”[All Fields]) OR (“substance-related disorders”[MeSH Terms]) OR ((“substance-related”[All Fields]) AND (“disorders”[All Fields])) OR (“substance-related disorders”[All Fields]) OR (“substance related disorders”[All Fields]) OR ((“substance”[All Fields]) AND (“related”[All Fields]) AND (“disorders”[All Fields])) OR (“alcohol-related disorders”[MeSH Terms]) OR (“alcohol-related disorders”[All Fields]) OR (“alcohol related disorders”[All Fields]) OR ((“alcohol-related”[All Fields]) AND (“disorders”[All Fields])) OR ((“alcohol”[All Fields]) AND (“related” [All Fields]) AND (“disorders” [All Fields])) OR (“amphetamine-related disorders”[MeSH Terms]) OR (“amphetamine-related disorders”[All Fields]) OR ((“amphetamine-related” [All Fields]) AND (“disorders” [All Fields])) OR (“amphetamine related disorders” [All Fields]) OR ((“amphetamine” [All Fields]) AND (“related” [All Fields]) AND (“disorders” [All Fields])) OR (“cocaine-related disorders”[MeSH Terms]) OR (“cocaine-related disorders”[All Fields]) OR ((“cocaine-related” [All Fields]) AND (“disorders” [All Fields])) OR (“cocaine related disorders” [All Fields]) OR ((“cocaine” [All Fields]) AND (“related” [All Fields]) AND (“disorders” [All Fields])) OR (“inhalant abuse”[MeSH Terms]) OR (“inhalant abuse”[All Fields]) OR ((“inhalant” [All Fields]) AND (“abuse” [All Fields])) OR (“marijuana abuse”[MeSH Terms]) OR (“marijuana abuse”[All Fields]) OR ((“marijuana” [All Fields]) AND (“abuse” [All Fields])) OR (“substance abuse intravenous”[MeSH Terms]) OR (“substance abuse intravenous”[All Fields]) OR ((“substance” [All Fields]) AND (“abuse” [All Fields]) AND (“intravenous” [All Fields])) OR (“tobacco use disorder”[MeSH Terms]) OR (“tobacco use disorder”[All Fields]) OR ((“tobacco” [All Fields]) AND (“use” [All Fields]) AND (“disorder” [All Fields])) OR (“drug utilization”[MeSH Terms]) OR (“drug utilization”[All Fields]) OR ((“drug” [All Fields]) AND (“utilization” [All Fields])) OR (“drug abuse”[All Fields]) OR ((“drug” [All Fields]) AND (“abuse” [All Fields])) OR (“drug dependency”[All Fields]) OR ((“drug” [All Fields]) AND (“dependency” [All Fields])) OR (“substance utilization”[All Fields]) OR ((“substance” [All Fields]) AND (“utilization” [All Fields])) OR (“substance abuse”[All Fields]) OR ((“substance” [All Fields]) AND (“abuse” [All Fields])) OR (“substance dependency”[All Fields]) OR ((“substance” [All Fields]) AND (“dependency” [All Fields])) OR (“cannabis”[MeSH Terms]) OR (“cannabis”[All Fields]) OR (“marihuana”[All Fields]) OR (“marijuana”[All Fields]) OR (“tetrahydrocannabinol”[All Fields]) OR (“thc”[All Fields]) OR (“cocaine”[MeSH Terms]) OR (“cocaine”[All Fields]) OR (“crack cocaine”[MeSH Terms]) OR (“crack cocaine”[All Fields]) OR ((“crack” [All Fields]) AND (“cocaine” [All Fields])) OR (“alcohol”[All Fields]) OR (“alcoholics”[MeSH Terms]) OR (“alcoholics”[All Fields]) OR (“amphetamine”[MeSH Terms]) OR (“amphetamine”[All Fields]) OR (“methamphetamine”[MeSH Terms]) OR (“methamphetamine”[All Fields]) OR (“n-methyl-3,4-methylenedioxyamphetamine”[MeSH Terms]) OR (“n-methyl-3,4-methylenedioxyamphetamine”[All Fields]) OR (“mdma”[All Fields]) OR (“ecstasy”[All Fields]) OR (“heroin”[MeSH Terms]) OR (“heroin”[All Fields]) OR (“nicotine”[MeSH Terms]) OR (“nicotine”[All Fields]) OR (“tobacco”[MeSH Terms]) OR (“tobacco”[All Fields])))) 322 results

**Meta-analytic connectivity modelling (MACM) and functional characterization**

We conducted meta-analytic connectivity modelling (MACM) to analyse co-activation patterns of regions-of-interest (ROI) resulting from the primary outcomes, aiming to functionally segregate each region’s putative contribution to behavioural domains^4^. Co-activation analyses were performed using Sleuth^5^ and GingerALE^6^ from the BrainMap database. To identify regions of significant convergence, we conducted an ALE meta-analysis over all foci retrieved after searching Sleuth by each addiction-related ROI independently, and included the experiment level search criteria of “context: normal mapping” and “activations: activation only”. Addiction-related ROIs were created with Mango (<http://rii.uthscsa.edu/mango//userguide.html>) with a 5mm-radius sphere. The functional characterization of addiction-related clusters was based on the “Behavioral Domain” (BD) meta-data categories available for each neuroimaging study in the BrianMap database which include action, perception, emotion, cognition and interoception.

**Fail-Safe N analysis (FSN)**

Coordinate-based meta-analyses such as ALE can be subject to different forms of publication bias which may impact results and invalidate findings (e.g., the file drawer problem). We performed the Fail-Safe N analysis (FSN) as a measure of robustness against potential publication bias. It refers to the amount of contra-evidence that can be added to a meta-analysis before the results change, and can be obtained for each cluster that survives thresholding in an ALE meta-analysis. A higher FSN indicates more stable results and hence a higher robustness. It is estimated for normal mapping that a 95% confidence interval for the number of studies that report no local maxima varies from 5 to 30 per 100 published studies. Using the upper bound and the fact that our meta-analysis consists of 60 experiments, a possible estimate for the number of experiments that remain in the file drawer is 18. Therefore, the minimum FSN was defined as 18 (Results in **Supplementary Table 7**).

**RESULTS**

**
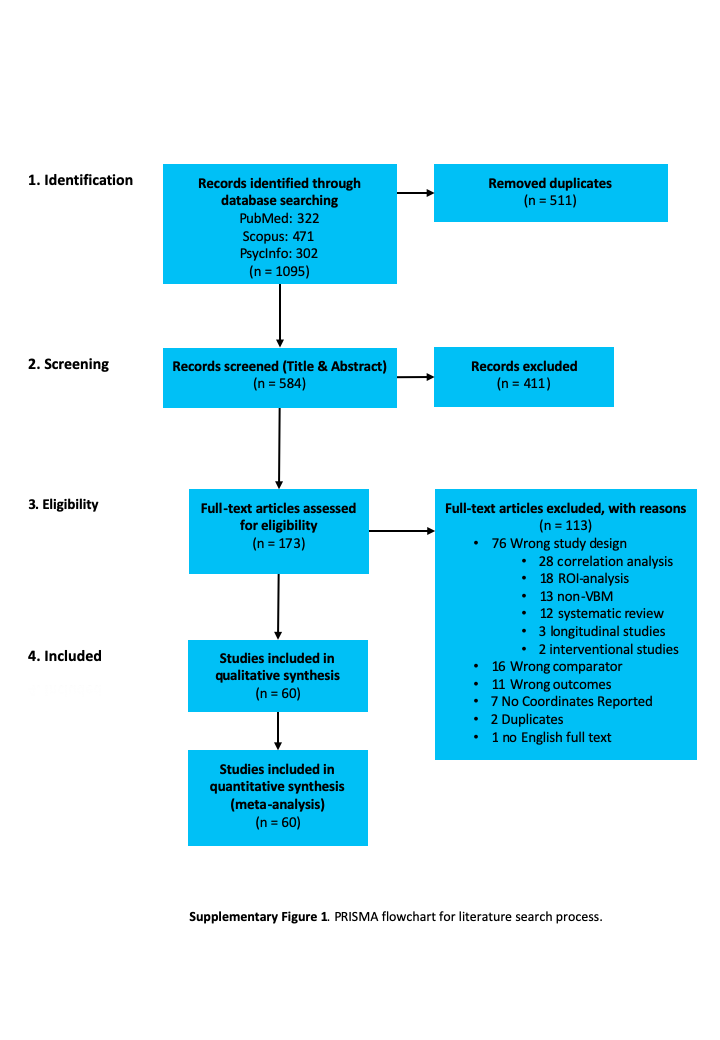
**

**PRISMA CHECKLIST**

SI = Supplementary Information

| **Section/topic** | **#** | **Checklist item** | **Reported on page #** |
| --- | --- | --- | --- |
| **TITLE** | | |  |
| Title | 1 | Identify the report as a systematic review, meta-analysis, or both. | 1 |
| **ABSTRACT** | | |  |
| Structured summary | 2 | Provide a structured summary including, as applicable: background; objectives; data sources; study eligibility criteria, participants, and interventions; study appraisal and synthesis methods; results; limitations; conclusions and implications of key findings; systematic review registration number. | 2 |
| **INTRODUCTION** | | |  |
| Rationale | 3 | Describe the rationale for the review in the context of what is already known. | 3 |
| Objectives | 4 | Provide an explicit statement of questions being addressed with reference to participants, interventions, comparisons, outcomes, and study design (PICOS). | 4 |
| **METHODS** | | |  |
| Protocol and registration | 5 | Indicate if a review protocol exists, if and where it can be accessed (e.g., Web address), and, if available, provide registration information including registration number. | 4 |
| Eligibility criteria | 6 | Specify study characteristics (e.g., PICOS, length of follow-up) and report characteristics (e.g., years considered, language, publication status) used as criteria for eligibility, giving rationale. | 5 & SI |
| Information sources | 7 | Describe all information sources (e.g., databases with dates of coverage, contact with study authors to identify additional studies) in the search and date last searched. | 4 & SI |
| Search | 8 | Present full electronic search strategy for at least one database, including any limits used, such that it could be repeated. | SI |
| Study selection | 9 | State the process for selecting studies (i.e., screening, eligibility, included in systematic review, and, if applicable, included in the meta-analysis). | 4 & SI |
| Data collection process | 10 | Describe method of data extraction from reports (e.g., piloted forms, independently, in duplicate) and any processes for obtaining and confirming data from investigators. | 5 & SI |
| Data items | 11 | List and define all variables for which data were sought (e.g., PICOS, funding sources) and any assumptions and simplifications made. | 5 & SI |
| Risk of bias in individual studies | 12 | Describe methods used for assessing risk of bias of individual studies (including specification of whether this was done at the study or outcome level), and how this information is to be used in any data synthesis. | 5 & SI |
| Summary measures | 13 | State the principal summary measures (e.g., risk ratio, difference in means). | 6 |
| Synthesis of results | 14 | Describe the methods of handling data and combining results of studies, if done, including measures of consistency (e.g., I^2^) for each meta-analysis. | 6 |

| **Section/topic** | **#** | **Checklist item** | **Reported on page #** |
| --- | --- | --- | --- |
| Risk of bias across studies | 15 | Specify any assessment of risk of bias that may affect the cumulative evidence (e.g., publication bias, selective reporting within studies). | 6 |
| Additional analyses | 16 | Describe methods of additional analyses (e.g., sensitivity or subgroup analyses, meta-regression), if done, indicating which were pre-specified. | 6 |
| **RESULTS** | | |  |
| Study selection | 17 | Give numbers of studies screened, assessed for eligibility, and included in the review, with reasons for exclusions at each stage, ideally with a flow diagram. | 6 & SI |
| Study characteristics | 18 | For each study, present characteristics for which data were extracted (e.g., study size, PICOS, follow-up period) and provide the citations. | 6 |
| Risk of bias within studies | 19 | Present data on risk of bias of each study and, if available, any outcome level assessment (see item 12). | 5, 7 & SI |
| Results of individual studies | 20 | For all outcomes considered (benefits or harms), present, for each study: (a) simple summary data for each intervention group (b) effect estimates and confidence intervals, ideally with a forest plot. | 7 |
| Synthesis of results | 21 | Present results of each meta-analysis done, including confidence intervals and measures of consistency. | 7 |
| Risk of bias across studies | 22 | Present results of any assessment of risk of bias across studies (see Item 15). | SI |
| Additional analysis | 23 | Give results of additional analyses, if done (e.g., sensitivity or subgroup analyses, meta-regression [see Item 16]). | SI |
| **DISCUSSION** | | |  |
| Summary of evidence | 24 | Summarize the main findings including the strength of evidence for each main outcome; consider their relevance to key groups (e.g., healthcare providers, users, and policy makers). | 9 |
| Limitations | 25 | Discuss limitations at study and outcome level (e.g., risk of bias), and at review-level (e.g., incomplete retrieval of identified research, reporting bias). | 12 |
| Conclusions | 26 | Provide a general interpretation of the results in the context of other evidence, and implications for future research. | 15 |
| **FUNDING** | | |  |
| Funding | 27 | Describe sources of funding for the systematic review and other support (e.g., supply of data); role of funders for the systematic review. | 15 |

| **Supplementary Table 1. Characteristics of MRI acquisition and analysis.** | | | | | | | | | | |
| --- | --- | --- | --- | --- | --- | --- | --- | --- | --- | --- |
|  | Author | Year | GM/WM | Teslas | MRI-system | MRI-model | Head-coil | T1w sequence | Voxel size (mm) | Analysis Software |
| 1 | Almeida | 2008 | GM | 1.5 | Siemens | Symphony | - | MPRAGE | 1x1x1 | SPM 2 |
| 2 | Aoki | 2013 | GM+WM | 1.5 | Philips | Gyroscan Intera | SENSE | 3D | 1x1x1 | SPM 8 |
| 3 | Bach | 2019a | GM | 3 | Siemens | Trio | - | MPRAGE | 1x1x1 | SPM12 |
| 4 | Bach | 2019b | GM | 3 | Siemens | Trio | - | MPRAGE | 1x1x1 | SPM12 |
| 5 | Banca | 2016 | GM | 3 | Siemens | Trio | 32-channel | 3D | 1x1x1 | SPM 8 |
| 6 | Barrós- | 2011 | GM | 1.5 | Siemens | Avanto | Quadrature | GE | 1x1x1 | SPM 5 VBM 7.0 |
| 7 | Battistella | 2014 | GM | 3 | Siemens | Trio | 32-channel | MPRAGE | 1x1x1 | SPM 8 |
| 8 | Brody | 2004 | GM | 1.5 | Siemens | Vision | - | SPGR | 1.5x1.5x1.5 | SPM 99 |
| 9 | Bu | 2016 | GM | 3 | Philips | Achieva | - | MPRAGE | 1x1x1 | SPM 8 VBM 8 |
| 10 | Chanraud | 2009 | GM | 1.5 | General Electric | - | - | IR- FSPGR | 0.94x0.94x1.3 | SPM 2 |
| 11 | Chanraud | 2007 | GM+WM | 1.5 | General Electric | Signa | - | IR- FSPGR | 0.94x0.94x1.3 | SPM 2 |
| 12 | Crunelle | 2014 | GM | 3 | Philips | Achieva | 32-channel | 3D | 1x1x1 | SPM 8 |
| 13 | Daumann | 2011 | GM | 3 | Siemens | Magnetom Trio Tim | Quadrature | 3D | 1x1x1.25 | FSL-VBM |
| 14 | Demirakca | 2011 | GM | 1.5 | Siemens | Vision | CP | MPRAGE | 1x1x1 | SPM8 |
| 15 | Filbey | 2014 | GM | 3 | Siemens | Trio | 12-channel | MPRAGE | 1x1x1 | DARTEL AFNI |
| 16 | Franklin | 2002 | GM+WM | 1.5 | General Electric | - | - | SPGR | 1x1x1 | SPM 99 |
| 17 | Franklin | 2014 | GM | 3 | Siemens | Trio | 8-channel | MPRAGE | 1x1x1 | SPM 8 |
| 18 | Fritz | 2014 | GM+WM | 1.5 | Siemens | Magnetom Avanto | - | MPRAGE | 1x1x1 | SPM 8 VBM 8 |
| 19 | Galandra | 2018 | GM | 3 | General Electric | Discovery | - | IR-FSPGR | 1x1x1 | SPM12 |
| 20 | Galandra | 2020 | GM | 3 | General Electric | Discovery | 16-channel | IR-FSPGR | 1x1x1 | SPM12 |
| 21 | Gallinat | 2006 | GM+WM | 3 | Bruker | Medspec | Quadrature | MDEFT | 1x1x1.5 | SPM 2 |
| 22 | Gardini | 2012 | GM+WM | 3 | Philips | Intera | - | TFE | 1x1x1 | SPM5 |
| 23 | Gilman | 2014 | GM+WM | 3 | Siemens | Trio | 32-channel | MPRAGE | 1x1x1 | FSL-VBM |
| 24 | Grodin | 2013 | GM | 1.5 | General Electric | - | Quadrature | MPRAGE | 0.94x0.94x2 | FSL-VBM |
| 25 | Hanlon | 2014 | GM | 3 | Siemens | TIM Trio | - | 3D | 1x1x1 | SPM8 DARTEL 10 |
| 26 | Hanlon | 2016 | GM | 3 | Siemens | TIM Trio | - | 3D | 1x1x1 | SPM 8 DARTEL 10 |
| 27 | Jang | 2007 | GM+WM | 3 | General Electric | Signa | - | SGR | 1x1x1 | SPM 2 |
| 28 | Jan | 2012 | GM | 1.5 | Siemens | Magnetom Avanto | - | MPRAGE | 1.25x1.25x1.25 | FSL-VBM 1.1 |
| 29 | Li | 2019 | GM | 3 | General Electric | Signa Excite | 8-channel | FSPGR | 1x1x1 | SPM5 |
| 30 | Liao | 2011 | GM | 3 | Siemens | Allegra | Birdcage | MPRAGE | 1x1x1 | SPM 5 VBM 5.1 |
| 31 | Liao | 2012 | GM | 3 | Siemens | Allegra | - | MPRAGE | 1x1x1 | SPM 5 VBM 5.1 |
| 32 | Lin | 2012 | GM | 3 | General Electric | Signa | 8 channel | IR-FSPGR | 0.5x0.5x1.3 | SPM 5 VBM 5 |
| 33 | Liu | 2009 | GM | 1.5 | General Electric | Signa Twinspeed | Birdcage | SPGR | 0.94x0.94x1.8 | SPM 2 VBM 2 |
| 34 | Lyoo | 2006 | GM | 1.5 | General Electric | - | Birdcage | SPGR | 1.5x1.5x1.5 | SPM 99 |
| 35 | Mackey | 2014 | GM | 3 | General Electric | Signa Excite | - | SPGR | 1x1x1 | FSL-VBM |
| 36 | Matochik | 2005 | GM+WM | 1.5 | General Electric | Signa | - | SPGR | 0.94x0.94x1.5 | SPM 99 |
| 37 | Matuskey | 2014 | GM | 3 | Siemens | Trio | CP | MPRAGE | 0.98x0.98x1 | FSL-VBM |
| 38 | Meade | 2020 | GM | 3 | General Electric | Discovery | 8-channel | 3D | 1x1x1 | FSL-VBM |
| 39 | Mechtcheriakov | 2007 | GM+WM | 1.5 | Siemens | Symphony | - | FLASH | 0.98x0.98x1.5 | SPM2 |
| 40 | Morales | 2012 | GM | 1.5 | Siemens | Sonata | Quadrature | MPRAGE | 1x1x1 | SPM 8 VBM 8 |
| 41 | Moreno-A | 2018 | GM | 1.5 | General Electric | Signa | - | 3D | 0.47x0.47x1 | FSL-VBM |
| 42 | Moreno-L | 2012 | GM+WM | 3 | Philips | Achieva | 8-channel | TFE | 0.94x0.94x1 | SPM 8 VBM 8 |
| 43 | Mwansisya | 2016 | GM | 1.5 | General Electric | Signa Twinspeed | Birdcage | SPGR | 0.94x0.94x1.8 | SPM 5 VBM 5 |
| 44 | Noyan | 2016 | GM | 1.5 | Philips | Achieva | 8-channel | MPRAGE | 0.94x0.94x1.2 | SPM 8 VBM 8 |
| 45 | Nurmedov | 2015 | GM | 1.5 | Philips | Achieva | 8-channel | MPRAGE | 1.25x1.25x1.2 | SPM 8 VBM 8 |
| 46 | Nurmedov | 2016 | GM | 1.5 | Philips | Achieva | 8-channel | MPRAGE | 0.94x0.94x1.2 | SPM 8 VBM 8 |
| 47 | Peng | 2015 | GM+WM | 3 | Siemens | Magnetom Trio Tim | 8-channel | 3D | 1x1x1 | SPM 8 DARTEL |
| 48 | Peng | 2018 | GM | 3 | Siemens | Magnetom Trio | 8-channel | MPRAGE | 1x1x1 | SPM8 |
| 49 | Potvin | 2007 | GM | 1.5 | Siemens | Magnetom Vision | - | 3D | 0.94x0.94x0.94 | SPM 2 |
| 50 | Qiu | 2013 | GM | 1.5 | Philips | Achieva Nova Dual | - | FFE | 0.99x0.99x1 | SPM 5 VBM 5 |
| 51 | Qiu | 2014 | GM | 1.5 | Philips | Achieva Nova Dual | 16-channel | FFE | 1x1x1 | SPM 8 VBM 8 |
| 52 | Segobin | 2014 | GM+WM | 1.5 | General Electric | Signa Advance | - | SPGR | 0.94x0.94x1.5 | SPM 5 VBM 5 |
| 53 | Sim | 2007 | GM+WM | 1.5 | General Electric | Horizon Echo-Speed | Birdcage | SPGR | 0.94x0.94x1.5 | SPM 2 |
| 54 | Stoeckel | 2016 | GM | 3 | Siemens | TrioTim | 32-channel | MPRAGE | 1x1.3x1.3 | SPM 8 VBM 8 |
| 55 | vanEijk | 2013 | GM+WM | 3 | Siemens | TrioTim | - | MPRAGE | 1x1x1 | SPM 8 VBM 8 |
| 56 | vanHolst | 2012 | GM | 3 | Phillips | Intera | SENSE | 3D | 1x1x1 | SPM 8 DARTEL |
| 57 | Wang | 2016 | GM | 3 | Siemens | Trio | - | MPRAGE | 1x1x1 | SPM8 |
| 58 | Wetherill | 2015 | GM | 3 | Siemens | Trio | 8-channel | 3D | 1x1x1 | SPM 8 VBM 8 |
| 59 | Yip | 2017 | GM | 3 | Siemens | Trio | - | MPRAGE | 1x1x1 | FSL VBM |
| 60 | Zhang | 2011 | GM | 3 | Siemens | Allegra | Birdcage | MPRAGE | 1x1x1 | FSL VBM |
| GM, grey matter; WM, white matter; MRI, magnetic resonance imaging; FFE, fast field echo sequence; FLASH, fast low angle shot sequence; FSL, functional MRI of the brain software library; GE, gradient echo pulse; IR-FSPGR, fast spoiled gradient sequence with inversion preparation; MPRAGE, magnetization-prepared rapid acquisition with gradient echo sequence; MDEFT, modified driven equilibrium Fourier transform; SPGR, spoiled gradient recalled sequence; SPM, statistical parametric mapping; TFE, turbo field echo sequence; VBM, voxel-based morphometry. | | | | | | | | | | |

| **Supplementary Table 2. Summary of MRI quality.** | | | | | | | | | | | | | | |
| --- | --- | --- | --- | --- | --- | --- | --- | --- | --- | --- | --- | --- | --- | --- |
|  | Author | Year | MRI design described | Age reported | Sample gender reported | Matched control group* | Sample handedness reported | Ethics approval reported | Image acquisition described | Image processing described | Statistical MRI-analysis described | Software package specified | Multiple comparison correction described | Figures  and  tables |
| 1 | Almeida | 2008 | Y | Y | Y | a,b,c,d | Y | Y | Y | Y | Y | Y | N | Y |
| 2 | Aoki | 2013 | Y | Y | Y | a,b,c | Y | Y | Y | Y | Y | Y | Y | Y |
| 3 | Bach | 2019a | Y | Y | Y | a | N | Y | Y | Y | Y | Y | Y | Y |
| 4 | Bach | 2019b | Y | Y | Y | a | N | Y | Y | Y | Y | Y | Y | Y |
| 5 | Banca | 2016 | Y | Y | Y | a,b | N | Y | Y | Y | Y | Y | Y | Y |
| 6 | Barrós- | 2011 | Y | Y | Y | a,b,c,d | Y | N | Y | Y | Y | Y | Y | Y |
| 7 | Battistella | 2014 | Y | Y | Y | a,b,d | N | Y | Y | Y | Y | Y | Y | Y |
| 8 | Brody | 2004 | Y | Y | Y | a,b,c | Y | Y | Y | Y | Y | Y | N | Y |
| 9 | Bu | 2016 | Y | Y | Y | a,b,c,d | Y | Y | Y | Y | Y | Y | Y | Y |
| 10 | Chanraud | 2009 | Y | Y | Y | a,b,c,d | Y | Y | Y | Y | Y | Y | Y | Y |
| 11 | Chanraud | 2007 | Y | Y | Y | a,b,c,d | Y | Y | Y | Y | Y | Y | Y | Y |
| 12 | Crunelle | 2014 | Y | Y | Y | a,b | N | Y | Y | Y | Y | Y | Y | Y |
| 13 | Daumann | 2011 | Y | Y | Y | a,b,d | N | Y | Y | Y | Y | Y | Y | Y |
| 14 | Demirakca | 2011 | Y | Y | Y | a,b | N | Y | Y | Y | Y | Y | Y | Y |
| 15 | Filbey | 2014 | Y | Y | Y | a,b,d | N | Y | Y | Y | Y | Y | Y | Y |
| 16 | Franklin | 2002 | Y | Y | Y | a | Y | Y | Y | Y | Y | Y | Y | Y |
| 17 | Franklin | 2014 | Y | Y | Y | a,b,d | N | Y | Y | Y | Y | Y | Y | Y |
| 18 | Fritz | 2014 | Y | Y | Y | N | N | Y | Y | Y | Y | Y | Y | Y |
| 19 | Galandra | 2018 | Y | Y | Y | a,b,d | N | Y | Y | Y | Y | Y | Y | Y |
| 20 | Galandra | 2020 | Y | Y | Y | a,b,d | N | Y | Y | Y | Y | Y | Y | Y |
| 21 | Gallinat | 2006 | Y | Y | Y | a,b,c | Y | Y | Y | Y | Y | Y | Y | Y |
| 22 | Gardini | 2012 | Y | Y | N | a,b | N | Y | Y | Y | Y | Y | U | Y |
| 23 | Gilman | 2014 | Y | Y | Y | a,b,c,d | Y | Y | Y | y | Y | Y | Y | Y |
| 24 | Grodin | 2013 | Y | Y | Y | a,b,d | N | Y | Y | Y | Y | Y | Y | Y |
| 25 | Hanlon | 2014 | Y | Y | Y | a,b,d | N | Y | Y | Y | Y | Y | Y | Y |
| 26 | Hanlon | 2016 | Y | Y | Y | a,b,d | N | Y | Y | Y | Y | Y | Y | Y |
| 27 | Jang | 2007 | Y | Y | Y | a,b,d | N | Y | Y | Y | Y | Y | Y | Y |
| 28 | Jan | 2012 | Y | Y | Y | a,b | N | Y | Y | Y | Y | Y | Y | Y |
| 29 | Li | 2019 | Y | Y | Y | a,b,c,d | Y | Y | Y | Y | Y | Y | N | Y |
| 30 | Liao | 2011 | Y | Y | Y | a,b,c | Y | Y | Y | Y | y | Y | Y | Y |
| 31 | Liao | 2012 | Y | Y | Y | a,b,c | Y | Y | Y | Y | Y | Y | Y | Y |
| 32 | Lin | 2012 | Y | Y | Y | a,b,c | N | Y | Y | Y | Y | Y | Y | Y |
| 33 | Liu | 2009 | Y | Y | Y | a,b,c,d | Y | Y | Y | Y | Y | Y | N | Y |
| 34 | Lyoo | 2006 | Y | Y | Y | a,b,c | Y | Y | Y | Y | Y | Y | Y | Y |
| 35 | Mackey | 2014 | Y | Y | Y | a,b,d | N | N | Y | Y | Y | Y | Y | Y |
| 36 | Matochik | 2005 | Y | Y | Y | a,b,c,d | Y | Y | Y | Y | Y | Y | Y | Y |
| 37 | Matuskey | 2014 | Y | Y | Y | a,b | N | N | Y | U | Y | Y | Y | Y |
| 38 | Meade | 2020 | Y | Y | Y | a,b,d | N | Y | Y | Y | Y | Y | Y | Y |
| 39 | Mechtcheriakov | 2007 | Y | Y | Y | a,b,d | N | Y | Y | Y | Y | Y | Y | Y |
| 40 | Morales | 2012 | Y | Y | Y | a,b,d | N | Y | Y | Y | Y | Y | Y | Y |
| 41 | Moreno-A | 2018 | Y | Y | Y | a,b,c | Y | Y | Y | Y | Y | Y | Y | Y |
| 42 | Moreno-L | 2012 | Y | Y | Y | a,b | N | Y | Y | Y | Y | Y | Y | Y |
| 43 | Mwansisya | 2016 | Y | Y | Y | a,b,c,d | Y | Y | Y | Y | Y | Y | N | Y |
| 44 | Noyan | 2016 | Y | Y | Y | a,b,d | N | N | Y | Y | Y | Y | Y | Y |
| 45 | Nurmedov | 2015 | Y | Y | Y | a,b,c,d | Y | Y | Y | Y | Y | Y | Y | Y |
| 46 | Nurmedov | 2016 | Y | Y | Y | a,b,c,d | N | Y | Y | Y | Y | Y | Y | Y |
| 47 | Peng | 2015 | Y | Y | Y | a,b,c,d | Y | Y | Y | Y | Y | Y | Y | Y |
| 48 | Peng | 2018 | Y | Y | Y | a,b,c,d | Y | Y | Y | Y | Y | Y | Y | Y |
| 49 | Potvin | 2007 | Y | Y | Y | a,b,d | N | Y | Y | Y | Y | Y | Y | Y |
| 50 | Qiu | 2013 | Y | Y | Y | a,b,c,d | Y | Y | Y | Y | Y | Y | Y | Y |
| 51 | Qiu | 2014 | Y | Y | Y | a,b,c,d | Y | Y | Y | Y | Y | Y | Y | Y |
| 52 | Segobin | 2014 | Y | Y | N | a,b,d | N | Y | Y | Y | Y | Y | Y | Y |
| 53 | Sim | 2007 | Y | Y | Y | a,b,c,d | Y | Y | Y | Y | Y | Y | Y | Y |
| 54 | Stoeckel | 2016 | Y | Y | Y | a,b,c,d | Y | Y | Y | Y | Y | Y | Y | Y |
| 55 | van Eijk | 2013 | Y | Y | Y | a,b | N | Y | Y | Y | Y | Y | Y | Y |
| 56 | van Holst | 2012 | Y | Y | Y | a,b | N | Y | Y | Y | Y | Y | Y | Y |
| 57 | Wang | 2016 | Y | Y | Y | a,b,c | Y | Y | Y | Y | Y | Y | Y | Y |
| 58 | Wetherill | 2015 | Y | Y | Y | a,b,d | N | Y | Y | Y | Y | Y | Y | Y |
| 59 | Yip | 2017 | Y | Y | Y | a,b | N | N | Y | Y | Y | Y | Y | Y |
| 60 | Zhang | 2011 | Y | Y | Y | a,b,c,d | Y | Y | Y | Y | Y | Y | Y | Y |
| Y, yes; N, no; U, unclear.  * a, age; b, sex; c, handedness; d, education. | | | | | | | | | | | | | |  |

| **Supplementary Table 3. Type of consumption subgroup anatomic likelihood estimation meta-analytic results for studies comparing brain morphological changes between SU and HC (all substances), at cluster level inference p < 0.05 (FWE).** | | | | | | | | |
| --- | --- | --- | --- | --- | --- | --- | --- | --- |
| Cluster number | Volume (mm^3^) | MNI coordinates | | | ALE | P | Z | Label (Side, region) |
|  |  | x | y | z |  |  |  |  |
| *Lower GM volume with use (SU < HC): Addiction* | | | | | | | | |
| 1 | 24088 | -2 | 14 | 58 | 3E-02 | 4E-06 | 4.5 | L Superior Frontal Gyrus BA6 |
|  |  | -10 | 58 | -16 | 3E-02 | 5E-06 | 4.4 | L Superior Frontal Gyrus BA10 |
|  |  | 0 | 48 | -16 | 3E-02 | 5E-06 | 4.4 | L Anterior Cingulate BA32 |
|  |  | 8 | 48 | 0 | 3E-02 | 6E-06 | 4.4 | R Anterior Cingulate BA32 |
|  |  | -2 | 36 | 30 | 2E-02 | 9E-06 | 4.3 | L Medial Frontal Gyrus BA6 |
|  |  | 0 | 24 | 46 | 2E-02 | 1E-05 | 4.2 | L Medial Frontal Gyrus BA6 |
|  |  | -4 | 46 | 16 | 2E-02 | 1E-05 | 4.2 | L Medial Frontal Gyrus BA9 |
|  |  | -2 | 20 | 32 | 2E-02 | 4E-05 | 3.9 | L Cingulate Gyrus BA32 |
|  |  | 8 | 22 | -14 | 2E-02 | 9E-05 | 3.8 | R Anterior Cingulate BA32 |
|  |  | 4 | 44 | 14 | 2E-02 | 9E-05 | 3.7 | R Anterior Cingulate BA32 |
|  |  | 2 | 0 | 52 | 2E-02 | 3E-04 | 3.4 | L Medial Frontal Gyrus BA6 |
|  |  | 6 | 38 | -14 | 2E-02 | 1E-03 | 3.1 | R Anterior Cingulate BA32 |
|  |  | 6 | 8 | 30 | 1E-02 | 1E-03 | 3.0 | R Cingulate Gyrus BA24 |
|  |  | -10 | 30 | -14 | 1E-02 | 2E-03 | 2.9 | L Anterior Cingulate BA24 |
|  |  | 8 | 40 | -22 | 1E-02 | 8E-03 | 2.4 | R Medial Frontal Gyrus BA11 |
|  |  | 2 | 36 | -30 | 1E-02 | 8E-03 | 2.4 | R Rectal Gyrus BA11 |
|  |  | -10 | 44 | -18 | 1E-02 | 1E-02 | 2.3 | L Medial Frontal Gyrus BA10 |
|  |  | 14 | 38 | -26 | 1E-02 | 1E-02 | 2.3 | R Inferior Frontal Gyrus BA11 |
|  |  | -2 | 50 | 2 | 1E-02 | 1E-02 | 2.2 | L Anterior Cingulate BA32 |
|  |  | -12 | 14 | 66 | 1E-02 | 1E-02 | 2.2 | L Superior Frontal Gyrus BA6 |
|  |  | 28 | 20 | -14 | 1E-02 | 1E-02 | 2.2 | R Claustrum |
|  |  | 18 | 38 | -14 | 1E-02 | 1E-02 | 2.2 | R Medial Frontal Gyrus BA10 |
|  |  | 14 | 34 | -6 | 1E-02 | 1E-02 | 2.2 | R Anterior Cingulate BA24 |
|  |  | 8 | 8 | 38 | 1E-02 | 2E-02 | 2.2 | R Cingulate Gyrus BA24 |
| 2 | 23968 | -6 | -24 | 0 | 4E-02 | 2E-08 | 5.5 | L Thalamus |
|  |  | 2 | -16 | 6 | 3E-02 | 5E-06 | 4.4 | L Thalamus |
|  |  | -16 | -32 | 0 | 2E-02 | 2E-05 | 4.1 | L Thalamus |
|  |  | 56 | -20 | -8 | 2E-02 | 2E-05 | 4.1 | R Superior Temporal Gyrus BA22 |
|  |  | 6 | -2 | 8 | 2E-02 | 4E-05 | 4.0 | R Thalamus |
|  |  | 32 | -16 | -6 | 2E-02 | 4E-05 | 3.9 | R Lentiform Nucleus |
|  |  | 40 | -18 | 10 | 2E-02 | 5E-05 | 3.9 | R Insula BA13 |
|  |  | 58 | -30 | 2 | 2E-02 | 3E-04 | 3.5 | R Superior Temporal Gyrus BA22 |
|  |  | 6 | -14 | 14 | 2E-02 | 3E-04 | 3.4 | Right Thalamus |
|  |  | 48 | -28 | 18 | 2E-02 | 3E-04 | 3.4 | R Insula BA13 |
|  |  | -12 | -4 | 12 | 2E-02 | 1E-03 | 3.1 | L Thalamus |
|  |  | 14 | -32 | 2 | 1E-02 | 2E-03 | 2.9 | R Thalamus |
|  |  | 60 | -24 | 14 | 1E-02 | 4E-03 | 2.6 | R Postcentral Gyrus BA40 |
|  |  | 20 | -26 | 4 | 1E-02 | 6E-03 | 2.5 | R Thalamus |
|  |  | 16 | -14 | -12 | 1E-02 | 6E-03 | 2.5 | R Brainstem |
|  |  | -8 | 8 | 10 | 1E-02 | 6E-03 | 2.5 | L Caudate |
|  |  | -6 | 16 | 16 | 1E-02 | 7E-03 | 2.5 | L Anterior Cingulate BA33 |
|  |  | 0 | -10 | 24 | 1E-02 | 9E-03 | 2.4 | L Cingulate Gyrus BA23 |
|  |  | -4 | -6 | 2 | 1E-02 | 9E-03 | 2.4 | L Thalamus |
|  |  | -8 | 6 | 16 | 1E-02 | 9E-03 | 2.4 | L Caudate |
|  |  | 18 | -24 | -6 | 1E-02 | 1E-02 | 2.3 | R Brainstem |
|  |  | 52 | -40 | 4 | 1E-02 | 1E-02 | 2.3 | R Superior Temporal Gyrus BA41 |
|  |  | 56 | -46 | 10 | 1E-02 | 1E-02 | 2.2 | R Superior Temporal Gyrus BA22 |
|  |  | -2 | 10 | 2 | 1E-02 | 1E-02 | 2.2 | L Caudate |
|  |  | -8 | -12 | 14 | 1E-02 | 1E-02 | 2.2 | L Thalamus |
| 3 | 10248 | -44 | -10 | -6 | 2E-02 | 6E-05 | 3.8 | L Insula BA13 |
|  |  | -36 | 22 | -2 | 1E-02 | 2E-03 | 2.9 | L Insula BA13 |
|  |  | -40 | 12 | -2 | 1E-02 | 2E-03 | 2.9 | L Insula BA13 |
|  |  | -40 | -18 | 14 | 1E-02 | 2E-03 | 2.8 | L Insula BA13 |
|  |  | -36 | -12 | 4 | 1E-02 | 4E-03 | 2.6 | L Claustrum |
|  |  | -40 | 4 | -2 | 1E-02 | 7E-03 | 2.5 | L Claustrum |
|  |  | -42 | 12 | 6 | 1E-02 | 7E-03 | 2.4 | L Insula BA13 |
|  |  | -46 | -2 | 2 | 1E-02 | 7E-03 | 2.4 | L Insula BA13 |
|  |  | -54 | 18 | 2 | 1E-02 | 8E-03 | 2.4 | L Precentral Gyrus BA44 |
|  |  | -48 | 16 | -2 | 1E-02 | 8E-03 | 2.4 | L Insula BA13 |
|  |  | -38 | -28 | 10 | 1E-02 | 1E-02 | 2.3 | L Transverse Temporal Gyrus BA41 |
|  |  | -60 | 2 | -4 | 1E-02 | 1E-02 | 2.3 | L Superior Temporal Gyrus |
|  |  | -50 | 2 | -4 | 1E-02 | 1E-02 | 2.2 | L Superior Temporal Gyrus BA22 |
|  |  | -50 | -26 | 14 | 1E-02 | 2E-02 | 2.1 | L Transverse Temporal Gyrus BA41 |
| *Lower GM volume with use (SU < HC): Long-term use* | | | | | | | | |
| 1 | 25976 | -42.4 | 8.8 | -19.6 | 2E-04 | 4E-02 | 1.8 | L Superior temporal gyrus BA38 |
| 2 | 21472 | 4 | -18 | -2 | 9E-03 | 7E-05 | 3.8 | R Thalamus |
|  |  | 4 | -6 | -4 | 9E-03 | 7E-05 | 3.8 | R Thalamus |
|  |  | -4 | -8 | -2 | 9E-03 | 8E-05 | 3.8 | L Thalamus |
|  |  | 22 | -18 | -10 | 8E-03 | 4E-04 | 3.3 | R Substantia nigra |
| 3 | 16720 | 40 | 11.3 | -30 | 1E-03 | 2E-02 | 2.1 | R Superior temporal gyrus BA38 |
| *Lower GM volume with use (SU < HC): Occasional use* | | | | | | | | |
| 1 | 18336 | -2 | 50 | 28 | 1E-02 | 5E-05 | 3.9 | L Medial frontal gyrus |
|  |  | -8 | 40 | 20 | 1E-02 | 8E-05 | 3.8 | L Anterior cingulate cortex |
|  |  | -16 | 36 | 18 | 1E-02 | 9E-05 | 3.7 | L Anterior cingulate cortex |
|  |  | -22 | 44 | 28 | 1E-02 | 2E-04 | 3.6 | L Superior frontal gyrus |
|  |  | 4 | 56 | 20 | 9E-03 | 3E-04 | 3.5 | R Medial frontal gyrus |
|  |  | -38 | 44 | 22 | 9E-03 | 4E-04 | 3.3 | L Superior frontal gyrus |
| *Higher GM volume with use (HC < SU): Addiction* | | | | | | | | |
| 1 | 26320 | -26 | -4 | 2 | 3E-02 | 1E-14 | 7.6 | L Putamen |
|  |  | -28 | 8 | -8 | 1E-02 | 9E-06 | 4.3 | L Putamen |
|  |  | -16 | 6 | -8 | 1E-02 | 7E-05 | 3.8 | L Lateral globus pallidus |
|  |  | -16 | 20 | -16 | 9E-03 | 3E-04 | 3.4 | L Subcallosal gyrus |
| 2 | 26000 | 22 | 18 | 4 | 1E-02 | 2E-05 | 4.1 | R Caudate |
|  |  | 38 | -14 | -6 | 1E-02 | 5E-05 | 3.9 | R Claustrum |
|  |  | 22 | 2 | -10 | 1E-02 | 9E-05 | 3.8 | R Lateral globus pallidus |
|  |  | 18 | 20 | -16 | 9E-03 | 3E-04 | 3.4 | R Subcallosal gyrus |
| *Higher GM volume with use (HC < SU): Long-term use* | | | | | | | | |
| 1 | 30008 | -5.3 | -57.3 | -28 | 5E-04 | 2E-02 | 2.1 | L Cerebellum |
| 2 | 21840 | 24 | -8 | 8 | 1E-02 | 4E-05 | 4.0 | R Lateral Globus Pallidus |
|  |  | 2 | -12 | 0 | 8E-03 | 3E-04 | 3.5 | L Thalamus |
| 3 | 11352 | 28 | -30 | 56 | 8E-03 | 3E-04 | 3.5 | R Postcentral Gyrus BA3 |
| 4 | 11352 | -32 | -30 | 58 | 8E-03 | 3E-04 | 3.5 | L Postcentral Gyrus BA3 |
| *Higher GM volume with use (HC < SU): Occasional use* | | | | | | | | |
| 1 | 36752 | -22 | 1.3 | -5.3 | 2E-04 | 2E-02 | 2.0 | L Putamen |
| 2 | 23736 | 30 | -68 | 28 | 2E-04 | 2E-02 | 2.1 | R Precuneus |
| 3 | 13176 | -20 | -78 | 26 | 1E-02 | 3E-05 | 4.0 | L Cuneus |
| 4 | 13176 | 30 | -10 | 44 | 1E-02 | 3E-05 | 4.0 | R Middle frontal gyrus |
| 5 | 13176 | -24 | -36 | 58 | 1E-02 | 3E-05 | 4.0 | L Postcentral gyrus |
| *Lower WM volume with use (SU < HC): Addiction* | | | | | | | | |
| 1 | 21160 | 8 | -26 | -2 | 1E-02 | 1E-04 | 3.7 | R Anterior thalamic radiation |
|  |  | 4 | -32 | 12 | 1E-02 | 1E-04 | 3.7 | R Corpus callosum |
|  |  | -4 | 2 | 24 | 1E-02 | 1E-04 | 3.6 | L Corpus callosum |
|  |  | 2 | 10 | 22 | 1E-02 | 1E-04 | 3.6 | R Corpus callosum |
|  |  | 32 | -32 | 0 | 1E-02 | 2E-04 | 3.5 | R Optic radiation |
|  |  | -4 | -14 | 18 | 1E-02 | 2E-04 | 3.5 | L Fornix |
|  |  | 6 | -22 | 12 | 1E-02 | 2E-04 | 3.5 | R Anterior thalamic radiation |
|  |  | 0 | -31 | 12 | 1E-02 | 2E-04 | 3.5 | Corpus callosum |
|  |  | 20 | -34 | 2 | 9E-03 | 7E-04 | 3.2 | R Fornix |
| 2 | 8624 | -6 | -38 | -14 | 1E-02 | 9E-05 | 3.7 | L Superior cerebellar peduncle |
|  |  | -8 | -28 | -30 | 1E-02 | 3E-04 | 3.4 | L Corticospinal tract |
|  |  | -14 | -30 | -18 | 9E-03 | 3E-04 | 3.4 | L Corticospinal tract |
| *Lower WM volume with use (SU < HC): Long-term use* | | | | | | | | |
| 1 | 14592 | -20 | -48 | 64 | 8E-03 | 4E-06 | 4.5 | L Corticospinal tract |
| *Lower WM volume with use (SU < HC): Occasional use* | | | | | | | | |
| 1 | 54176 | 10 | -52 | 6 | 1E-02 | 4E-06 | 4.4 | R Cingulum |
|  |  | 13 | -54 | 24 | 1E-02 | 1E-05 | 4.2 | R Cingulum |
|  |  | -12 | -27 | 8 | 1E-02 | 2E-05 | 4.1 | L Fornix |
|  |  | -5 | -26 | -2 | 1E-02 | 3E-05 | 4.0 | L Anterior thalamic radiation |
|  |  | 5 | -27 | -2 | 9E-03 | 4E-05 | 3.9 | R Anterior thalamic radiation |
|  |  | 11 | -33 | 9 | 9E-03 | 8E-05 | 3.8 | R Fornix |
| 2 | 12856 | 9 | 45 | -20 | 9E-03 | 1E-04 | 3.7 | R Forceps minor |
| *Higher WM volume with use (HC < SU): Addiction* | | | | | | | | |
| - | - | - | - | - | - | - | - | - |
| *Higher WM volume with use (HC < SU): Long-term use* | | | | | | | | |
| 1 | 27736 | 14 | -14 | -16 | 2E-04 | 1E-02 | 2.3 | R Corticospinal tract |
| 2 | 14712 | -46 | -6 | -30 | 8E-03 | 2E-05 | 4.2 | L Inferior longitudinal fasciculus |
| 3 | 14712 | -22 | -24 | -8 | 8E-03 | 2E-05 | 4.2 | L Optic radiation |
| *Higher WM volume with use (HC < SU): Occasional use* | | | | | | | | |
| - | - | - | - | - | - | - | - | - |
| GM, grey matter; WM, white matter; SU, substance user; HC, healthy control; ALE, anatomic likelihood estimation; P, p-value; Z, peak z-value; R, right; L, left. | | | | | | | | |

| **Supplementary Table 4. Contrast analysis of type of consumption subgroup anatomic likelihood estimation meta-analytic results for studies comparing brain morphological changes between SU and HC (all substances), at cluster level inference p < 0.05 (FWE).** | | | | | | | | |
| --- | --- | --- | --- | --- | --- | --- | --- | --- |
| Cluster number | Volume (mm^3^) | MNI coordinates | | | ALE | P | Z | Label (Side, region) |
|  |  | x | y | z |  |  |  |  |
| *Lower GM volume with use (SU < HC): Addiction + long-term use* | | | | | | | | |
| 1 | 4808 | 4 | -18 | -2 | 9E-03 | - | - | R Thalamus |
|  |  | -4 | -8 | 0 | 9E-03 | - | - | L Thalamus |
|  |  | 6 | -6 | -4 | 8E-03 | - | - | R Thalamus |
|  |  | 22 | -18 | -10 | 8E-03 | - | - | R Brainstem |
|  |  | -40 | 8 | -6 | 3E-03 | - | - | L Insula |
|  |  | -50 | 4 | -6 | 3E-04 | - | - | L Superior Temporal Gyrus |
|  |  | -50 | 14 | -4 | 2E-04 | - | - | L Insula |
|  |  | -48 | 16 | -6 | 2E-04 | - | - | L Insula |
| *Lower GM volume with use (SU < HC): Addiction – long-term use* | | | | | | | | |
| 1 | 1664 | 8 | -18 | -10 | - | 2E-02 | 2.1 | R Red nucleus |
|  |  | 6 | -10 | -14 | - | 2E-02 | 2.0 | R Mamillary body |
|  |  | 18 | -22 | -14 | - | 2E-02 | 2.0 | R Hypothalamus |
| 2 | 680 | 22 | -16 | -15 | - | 3E-02 | 1.9 | R Sustantia nigra |
|  |  | 24 | -26 | -14 | - | 3E-02 | 1.9 | R Parahippocampal gyrus |
|  |  | -12 | 0 | -2 | - | 4E-02 | 1.7 | R Parahippocampal gyrus |
|  |  | -6 | 2 | -6 | - | 4E-02 | 1.7 | R Parahippocampal gyrus |
|  |  | -10 | -6 | -6 | - | 4E-02 | 1.7 | R Parahippocampal gyrus |
| 3 | 112 | -16 | 8 | -8 | - | 3E-02 | 1.8 | L Putamen |
| *Lower GM volume with use (SU < HC): Long-term use + occasional use* | | | | | | | | |
| - | - | - | - | - | - | - | - | - |
| *Lower GM volume with use (SU < HC): Long-term use – occasional use* | | | | | | | | |
| - | - | - | - | - | - | - | - | - |
| *Lower GM volume with use (SU < HC): Occasional use + addiction* | | | | | | | | |
| 1 | 1240 | -8 | 42 | 20 | 9E-03 | - | - | L Anterior Cingulate BA32 |
|  |  | -6 | 48 | 24 | 3E-03 | - | - | L Medial Frontal Gyrus BA9 |
|  |  | 2 | 50 | 16 | 2E-03 | - | - | L Medial Frontal Gyrus BA9 |
| 2 | 40 | -4 | 38 | 26 | 1E-03 | - | - | L Anterior Cingulate BA32 |
| 3 | 32 | -6 | 40 | 28 | 8E-04 | - | - | L Medial Frontal Gyrus BA9 |
| 4 | 32 | -2 | 42 | 30 | 9E-04 | - | - | L Medial Frontal Gyrus BA9 |
| 5 | 8 | -2 | 36 | 24 | 9E-04 | - | - | L Anterior Cingulate BA32 |
| *Lower GM volume with use (SU < HC): Occasional use - addiction* | | | | | | | | |
| 1 | 6472 | 0 | 50 | 24 | - | 4E-03 | 2.6 | L Medial frontal gyrus |
|  |  | -1.2 | 56.4 | 24 | - | 5E-03 | 2.6 | L Medial frontal gyrus |
|  |  | -14 | 40 | 26 | - | 6E-03 | 2.5 | L Medial frontal gyrus |
|  |  | -12 | 40 | 20 | - | 7E-03 | 2.4 | L Medial frontal gyrus |
|  |  | -2 | 44 | 24 | - | 1E-02 | 2.3 | L Medial frontal gyrus |
|  |  | -14 | 44 | 28 | - | 1E-02 | 2.2 | L Anterior cingulate cortex |
|  |  | -15 | 30 | 16 | - | 2E-02 | 2.1 | L Medial frontal gyrus |
|  |  | -18 | 28 | 22 | - | 2E-02 | 2.1 | L Medial frontal gyrus |
|  |  | -6 | 48 | 26 | - | 2E-02 | 2.0 | L Anterior cingulate cortex |
|  |  | -20 | 34 | 20 | - | 3E-02 | 1.9 | L Medial frontal gyrus |
|  |  | -10 | 46 | 30 | - | 3E-02 | 1.9 | L Superior temporal gyrus |
| 2 | 824 | -49.3 | -1.7 | -11.7 | - | 6E-03 | 2.5 | L Superior temporal gyrus |
| 3 | 112 | 0 | 60 | -6 | - | 3E-02 | 1.8 | L Superior temporal gyrus |
|  |  | -4 | 64 | -6 | - | 5E-02 | 1.7 | L Superior temporal gyrus |
| *Higher GM volume with use (HC < SU): Addiction + long-term use* | | | | | | | | |
| 1 | 224 | 22 | -2 | 0 | 2E-04 | - | - | R Lateral Globus Pallidus |
| 2 | 136 | 30 | -12 | 0 | 1E-04 | - | - | R Putamen |
|  |  | 32 | -8 | 0 | 1E-04 | - | - | R Putamen |
| *Higher GM volume with use (HC < SU): Addiction – long-term use* | | | | | | | | |
| 1 | 6368 | 8.3 | -19.5 | -0.2 | - | 3E-03 | 2.8 | R Thalamus |
|  |  | 8 | -16 | -10 | - | 5E-03 | 2.5 | R Red Nucleus |
|  |  | 14 | -14 | -4 | - | 7E-03 | 2.5 | R Thalamus |
|  |  | 18 | -18 | 4 | - | 1E-02 | 2.2 | R Thalamus VPLN |
|  |  | 0.1 | -19.7 | -6.1 | - | 3E-02 | 1.8 | L Red Nucleus |
|  |  | 11.5 | -9.5 | 7.3 | - | 4E-02 | 1.8 | R Thalamus |
|  |  | 10 | -12 | -8.7 | - | 7E-02 | 0.0 | R Subthalamic Nucleus |
| *Higher GM volume with use (HC < SU): Long-term use + occasional use* | | | | | | | | |
| 1 | 6080 | -28 | -32 | 58 | 3E-03 | - | - | L Postcentral gyrus |
| 2 | 880 | -5 | -5 | -6 | 1E-04 | - | - | L Hypothalamus |
| 3 | 576 | 30 | -20 | 50 | 8E-05 | - | - | R Precentral gyrus |
|  |  | 28 | -22 | 46 | 4E-05 | - | - | R Cingulate gyrus |
| *Higher GM volume with use (HC < SU): Long-term use – occasional use* | | | | | | | | |
| - | - | - | - | - | - | - | - | - |
| *Higher GM volume with use (HC < SU): Occasional use + addiction* | | | | | | | | |
| 1 | 13992 | -14 | 4 | -8 | 6E-03 | - | - | L Globus pallidus |
|  |  | -24 | -8 | -4 | 3E-03 | - | - | L Globus pallidus |
|  |  | -28 | 10 | -2 | 2E-03 | - | - | L Putamen |
| *Higher GM volume with use (HC < SU): Occasional use - addiction* | | | | | | | | |
| - | - | - | - | - | - | - | - | - |
| *Lower WM volume with use (SU < HC): Addiction + long-term use* | | | | | | | | |
| - | - | - | - | - | - | - | - | - |
| *Lower WM volume with use (SU < HC): Addiction – long-term use* | | | | | | | | |
| - | - | - | - | - | - | - | - | - |
| *Lower WM volume with use (SU < HC): Long-term use + occasional use* | | | | | | | | |
| - | - | - | - | - | - | - | - | - |
| *Lower WM volume with use (SU < HC): Long-term use – occasional use* | | | | | | | | |
| - | - | - | - | - | - | - | - | - |
| *Lower WM volume with use (SU < HC): Occasional use + addiction* | | | | | | | | |
| 1 | 9440 | 6 | -26 | -2 | 9E-03 | - | - | R Anterior thalamic radiation |
|  |  | 8 | -32 | 12 | 5E-03 | - | - | R Corpus callosum |
|  |  | 14 | -34 | 6 | 3E-03 | - | - | R Fornix |
|  |  | -6 | -28 | 10 | 2E-03 | - | - | L Anterior thalamic radiation |
|  |  | -6 | -20 | 12 | 2E-04 | - | - | L Anterior thalamic radiation |
| 2 | 768 | -5 | -32 | -8 | 4E-04 | - | - | L Anterior thalamic radiation |
| 3 | 8 | 2 | -36 | -12 | 6E-06 | - | - | R Anterior thalamic radiation |
| 4 | 8 | 2 | -38 | -10 | 6E-06 | - | - | R Anterior thalamic radiation |
| 5 | 8 | -4 | -16 | 10 | 8E-06 | - | - | L Anterior thalamic radiation |
| *Lower WM volume with use (SU < HC): Occasional use - addiction* | | | | | | | | |
| - | - | - | - | - | - | - | - | - |
| *Higher WM volume with use (HC < SU): Addiction + long-term use* | | | | | | | | |
| - | - | - | - | - | - | - | - | - |
| *Higher WM volume with use (HC < SU): Addiction – long-term use* | | | | | | | | |
| - | - | - | - | - | - | - | - | - |
| *Higher WM volume with use (HC < SU): Long-term use + occasional use* | | | | | | | | |
| - | - | - | - | - | - | - | - | - |
| *Higher WM volume with use (HC < SU): Long-term use – occasional use* | | | | | | | | |
| - | - | - | - | - | - | - | - | - |
| *Higher WM volume with use (HC < SU): Occasional use + addiction* | | | | | | | | |
| - | - | - | - | - | - | - | - | - |
| *Higher WM volume with use (HC < SU): Occasional use - addiction* | | | | | | | | |
| - | - | - | - | - | - | - | - | - |
| GM, grey matter; WM, white matter; SU, substance user; HC, healthy control; ALE, anatomic likelihood estimation; P, p-value; Z, peak z-value; R, right; L, left. | | | | | | | | |

| **Supplementary Table 5. Type of substance subgroup anatomic likelihood estimation meta-analytic results for studies comparing brain morphological changes between SU and HC, at cluster level inference p < 0.05 (FWE).** | | | | | | | | |
| --- | --- | --- | --- | --- | --- | --- | --- | --- |
| Cluster number | Volume (mm^3^) | MNI coordinates | | | ALE | P | Z | Label (Side, region) |
|  |  | x | y | z |  |  |  |  |
| *Lower GM volume with use (SU < HC): Alcohol* | | | | | | | | |
| 1 | 13400 | 0 | 34 | 30 | 2E-02 | 4E-06 | 4.4 | L Anterior Cingulate Gyrus BA32 |
|  |  | -2 | 22 | 32 | 2E-02 | 5E-06 | 4.4 | L Anterior Cingulate Gyrus BA32 |
|  |  | -2 | 46 | 14 | 2E-02 | 2E-05 | 4.2 | L Anterior Cingulate BA32 |
|  |  | 2 | 0 | 54 | 1E-02 | 2E-04 | 3.5 | L Medial Frontal Gyrus BA6 |
|  |  | -12 | 44 | 10 | 1E-02 | 1E-03 | 3.0 | L Medial Frontal Gyrus BA9 |
|  |  | 6 | 50 | -4 | 1E-02 | 1E-03 | 3.0 | R Anterior Cingulate BA32 |
|  |  | -2 | 4 | 46 | 1E-02 | 2E-03 | 2.9 | L Cingulate Gyrus BA24 |
|  |  | 8 | 44 | 4 | 1E-02 | 3E-03 | 2.7 | R Anterior Cingulate BA32 |
|  |  | -16 | 36 | 18 | 1E-02 | 3E-03 | 2.7 | L Anterior Cingulate BA32 |
|  |  | 0 | 16 | 50 | 1E-02 | 4E-03 | 2.7 | L Superior Frontal Gyrus BA6 |
|  |  | -2 | 50 | 2 | 9E-03 | 4E-03 | 2.7 | L Anterior Cingulate BA32 |
| *Lower GM volume with use (SU < HC): Tobacco* | | | | | | | | |
| 1 | 19064 | -8 | -52 | -2 | 2E-02 | 3E-07 | 5.0 | L Culmen |
|  |  | -4 | -54 | -2 | 2E-02 | 3E-07 | 5.0 | L Culmen |
|  |  | -8 | -66 | 16 | 2E-02 | 5E-07 | 4.9 | L Posterior Cingulate |
|  |  | -4 | -66 | 12 | 2E-02 | 7E-06 | 4.3 | L Cuneus |
|  |  | 8 | -66 | 6 | 1E-02 | 1E-04 | 3.7 | R Lingual Gyrus |
|  |  | 18 | -68 | 20 | 1E-02 | 2E-04 | 3.5 | R Posterior Cingulate |
|  |  | 24 | -66 | 12 | 1E-02 | 8E-04 | 3.1 | R Posterior Cingulate |
|  |  | 14 | -74 | 28 | 1E-02 | 9E-04 | 3.1 | R Cuneus |
|  |  | 16 | -74 | 8 | 1E-02 | 1E-03 | 3.1 | R Lingual Gyrus |
|  |  | 6 | -66 | 18 | 1E-02 | 1E-03 | 3.1 | R Posterior Cingulate |
|  |  | -16 | -58 | 16 | 1E-02 | 1E-03 | 3.1 | L Posterior Cingulate |
| 2 | 8928 | -6 | -26 | 0 | 3E-02 | 5E-10 | 6.1 | L Thalamus |
|  |  | 4 | -18 | 2 | 1E-02 | 4E-04 | 3.4 | R Thalamus |
|  |  | -22 | -34 | 0 | 1E-02 | 8E-04 | 3.1 | L Parahippocampal Gyrus |
|  |  | 6 | -16 | 14 | 1E-02 | 9E-04 | 3.1 | R Thalamus |
|  |  | 20 | -18 | 14 | 1E-02 | 1E-03 | 3.1 | R Thalamus |
| 3 | 7736 | -6 | 10 | 10 | 2E-02 | 7E-06 | 4.4 | L Caudate |
|  |  | -6 | 14 | 16 | 1E-02 | 2E-04 | 3.5 | L Caudate |
|  |  | -4 | 32 | 6 | 1E-02 | 1E-03 | 3.0 | L Anterior Cingulate |
|  |  | -8 | 30 | 20 | 9E-03 | 2E-03 | 2.9 | L Anterior Cingulate |
|  |  | -12 | -4 | 12 | 9E-03 | 2E-03 | 2.9 | L Thalamus |
| 4 | 7600 | -40 | 12 | -2 | 1E-02 | 2E-04 | 3.6 | L Insula |
|  |  | -36 | -12 | 4 | 1E-02 | 3E-04 | 3.4 | L Claustrum |
|  |  | -42 | 12 | 6 | 1E-02 | 5E-04 | 3.3 | L Insula |
|  |  | -40 | -16 | 0 | 1E-02 | 6E-04 | 3.2 | L Claustrum |
|  |  | -46 | 4 | -10 | 1E-02 | 7E-04 | 3.2 | L Superior Temporal Gyrus |
|  |  | -48 | 16 | -2 | 1E-02 | 9E-04 | 3.1 | L Insula |
|  |  | -44 | -10 | -8 | 1E-02 | 1E-03 | 3.0 | L Insula |
| *Lower GM volume with use (SU < HC): Cocaine* | | | | | | | | |
| 1 | 11656 | 28 | 20 | -16 | 1E-02 | 2E-04 | 3.5 | R Claustrum |
|  |  | 36 | 6 | -8 | 9E-03 | 5E-04 | 3.3 | R Claustrum |
|  |  | 36 | 12 | 0 | 9E-03 | 5E-04 | 3.3 | R Claustrum |
|  |  | 30 | 8 | -26 | 9E-03 | 7E-04 | 3.2 | R Inferior frontal gyrus |
|  |  | 23.3 | -5.3 | -24 | 9E-03 | 7E-04 | 3.2 | R Parahippocampal gyrus |
| *Lower GM volume with use (SU < HC): Cannabis* | | | | | | | | |
| 1 | 23304 | -50 | 10 | -14 | 1E-02 | 8E-05 | 3.8 | L Superior temporal gyrus |
|  |  | -36 | 8 | -10 | 1E-02 | 8E-05 | 3.8 | L Claustrum |
|  |  | -56 | 2 | -28 | 1E-02 | 8E-05 | 3.8 | L Middle temporal gyrus |
|  |  | -35 | 12 | -23 | 0E+00 | 1E-01 | 1.3 | L Inferior frontal gyrus |
|  |  | -48 | -6 | -12 | 1E-02 | 8E-05 | 3.8 | L Superior temporal gyrus |
| 2 | 17848 | 2 | -16 | 6 | 1E-02 | 1E-05 | 4.2 | L Thalamus / Medial Dorsal Nucleus |
|  |  | 4 | -18 | 0 | 1E-02 | 2E-05 | 4.2 | R Thalamus |
|  |  | 4 | -6 | -4 | 9E-03 | 1E-04 | 3.7 | R Thalamus |
|  |  | -4 | -8 | -2 | 9E-03 | 1E-04 | 3.7 | L Thalamus |
|  |  | 22 | -18 | -10 | 8E-03 | 7E-04 | 3.2 | R Substania nigra |
| *Lower GM volume with use (SU < HC): Opioids* | | | | | | | | |
| 1 | 11496 | -4 | 12 | -16 | 1E-02 | 1E-04 | 3.7 | L Anterior cingulate cortex |
|  |  | -16 | 20 | -28 | 1E-02 | 2E-04 | 3.5 | L Inferior frontal gyrus |
|  |  | -12 | 4 | -23 | 1E-02 | 2E-04 | 3.5 | L Subcallosal gyrus |
|  |  | -30 | 19 | -17 | 9E-03 | 4E-04 | 3.4 | L Orbitofrontal cortex |
|  |  | -8 | 34 | -32 | 6E-03 | 5E-03 | 2.6 | L Rectal gyrus |
| 2 | 9504 | 32 | 56 | 30 | 1E-02 | 6E-05 | 3.8 | R Superior frontal gyrus |
|  |  | -4 | 38 | 30 | 1E-02 | 2E-04 | 3.5 | L Medial frontal gyrus |
|  |  | 12 | 38 | 38 | 9E-03 | 2E-04 | 3.5 | R Medial frontal gyrus |
|  |  | 22 | 56 | 40 | 9E-03 | 7E-04 | 3.2 | R Superior frontal gyrus |
| 3 | 9056 | 44 | -2 | 12 | 1E-02 | 5E-05 | 3.9 | R Insula |
|  |  | 44 | -36 | 18 | 9E-03 | 3E-04 | 3.5 | R Superior temporal gyrus |
|  |  | 48 | -18 | 14 | 9E-03 | 4E-04 | 3.4 | R Insula |
| 4 | 9040 | 32 | 28 | 36 | 1E-02 | 1E-04 | 3.6 | R Middle frontal gyrus |
|  |  | 52 | 22 | 22 | 9E-03 | 3E-04 | 3.5 | R Inferior frontal gyrus |
|  |  | 42 | 32 | 22 | 8E-03 | 9E-04 | 3.1 | R Middle frontal gyrus |
| *Lower GM volume with use (SU < HC): Stimulants* | | | | | | | | |
| 1 | 49544 | -2 | 50 | 28 | 1E-02 | 5E-06 | 4.4 | L Medial frontal gyrus |
|  |  | 2 | 52 | -16 | 1E-02 | 2E-05 | 4.1 | R Medial frontal gyrus |
|  |  | 6 | 48 | 0 | 1E-02 | 2E-05 | 4.1 | R Anterior cingulate cortex |
|  |  | -2 | 48 | -22 | 1E-02 | 2E-05 | 4.1 | L Medial frontal gyrus |
|  |  | 26 | 60 | -8 | 1E-02 | 3E-05 | 4.0 | R Superior frontal gyrus |
|  |  | 4 | 56 | 20 | 9E-03 | 4E-05 | 3.9 | R Medial frontal gyrus |
|  |  | -2 | 62 | -6 | 9E-03 | 9E-05 | 3.7 | L Medial frontal gyrus |
| 2 | 14392 | -6 | 13 | 63 | 2E-03 | 7E-03 | 2.5 | L Superior frontal gyrus |
| 3 | 9512 | -2 | -38 | 52 | 1E-02 | 4E-05 | 3.9 | L Precuneus |
| 4 | 9128 | -44 | 42 | -6 | 1E-02 | 3E-05 | 4.0 | L Middle frontal gyrus |
| 5 | 9120 | -57 | -6 | 4 | 9E-03 | 6E-05 | 3.9 | L Precentral gyrus |
| *Lower GM volume with use (SU < HC): Polysubstance* | | | | | | | | |
| 1 | 16280 | 0 | -10 | 24 | 1E-02 | 7E-05 | 3.8 | L Cingulate gyrus |
|  |  | 2 | -16 | 6 | 1E-02 | 8E-05 | 3.8 | L Thalamus / Medial Dorsal Nucleus |
|  |  | -4 | -20 | -2 | 1E-02 | 1E-04 | 3.7 | L Thalamus |
|  |  | -4 | -6 | 2 | 1E-02 | 1E-04 | 3.6 | L Thalamus |
|  |  | 8 | -2 | -2 | 1E-02 | 3E-04 | 3.5 | R Thalamus |
|  |  | -4 | 10 | 0 | 9E-03 | 4E-04 | 3.4 | L Caudate |
| 2 | 8416 | 66 | 6 | -8 | 1E-02 | 1E-04 | 3.7 | R Superior temporal gyrus |
|  |  | 66 | -8 | 4 | 1E-02 | 2E-04 | 3.6 | R Superior temporal gyrus |
|  |  | 70 | -24 | -6 | 1E-02 | 2E-04 | 3.5 | R Superior temporal gyrus |
|  |  | 70 | -14 | 8 | 9E-03 | 3E-04 | 3.4 | R Transverse temporal gyrus |
| 3 | 7872 | -66 | -10 | 4 | 1E-02 | 2E-04 | 3.6 | L Superior temporal gyrus |
|  |  | -60 | 2 | -4 | 1E-02 | 2E-04 | 3.6 | L Superior temporal gyrus |
|  |  | -66 | -4 | -16 | 1E-02 | 2E-04 | 3.6 | L Middle temporal gyrus |
| *Higher GM volume with use (HC < SU): Alcohol* | | | | | | | | |
| - | - | - | - | - | - | - | - | - |
| *Higher GM volume with use (HC < SU): Tobacco* | | | | | | | | |
| 1 | 26952 | 8 | -66 | 6 | 2E-02 | 2E-08 | 5.5 | R Lingual gyrus |
|  |  | 20 | -40 | -8 | 1E-02 | 1E-05 | 4.2 | R Parahippocampal gyrus |
| 2 | 25768 | -26 | -4 | 4 | 1E-02 | 4E-07 | 5.0 | L Putamen |
|  |  | -28 | 8 | -8 | 1E-02 | 1E-05 | 4.2 | L Putamen |
| 3 | 12128 | 38 | -14 | -6 | 1E-02 | 3E-05 | 4.0 | R Claustrum |
| 4 | 12024 | 22 | 18 | 4 | 1E-02 | 1E-05 | 4.2 | R Caudate |
| 5 | 7752 | -48 | -82 | 10 | 1E-02 | 2E-05 | 4.1 | L Middle occipital gyrus |
| *Higher GM volume with use (HC < SU): Cocaine* | | | | | | | | |
| - | - | - | - | - | - | - | - | - |
| *Higher GM volume with use (HC < SU): Cannabis* | | | | | | | | |
| 1 | 26352 | -26 | -4 | 2 | 1E-02 | 9E-06 | 4.3 | L Putamen |
|  |  | -24 | -12 | -8 | 1E-02 | 4E-05 | 3.9 | L Lateral globus pallidus |
|  |  | -30 | 14 | 2 | 1E-02 | 7E-05 | 3.8 | L Claustrum |
|  |  | -12 | 2 | -10 | 1E-02 | 7E-05 | 3.8 | L Medial globus pallidus |
|  |  | 2 | -12 | 0 | 8E-03 | 5E-04 | 3.3 | L Thalamus |
| 2 | 16208 | 26 | -16 | 64 | 1E-02 | 2E-05 | 4.2 | R Precentral gyrus |
|  |  | 30 | -10 | 44 | 1E-02 | 7E-05 | 3.8 | R Middle frontal gyrus |
|  |  | 28 | -30 | 56 | 8E-03 | 5E-04 | 3.3 | R Postcentral gyrus |
| *Higher GM volume with use (HC < SU): Opioids* | | | | | | | | |
| - | - | - | - | - | - | - | - | - |
| *Higher GM volume with use (HC < SU): Stimulants* | | | | | | | | |
| - | - | - | - | - | - | - | - | - |
| *Higher GM volume with use (HC < SU): Polysubstance* | | | | | | | | |
| 1 | 13824 | 18 | 20 | -16 | 9E-03 | 3E-05 | 4.0 | R Subcallosal gyrus |
| 2 | 13800 | -16 | 20 | -16 | 9E-03 | 3E-05 | 4.0 | L Subcallosal gyrus |
| 3 | 12408 | -26 | -4 | 2 | 1E-02 | 4E-06 | 4.5 | L Putamen |
| *Lower WM volume with use (SU < HC): Alcohol* | | | | | | | | |
| 1 | 12760 | -8 | -28 | -32 | 1E-02 | 8E-05 | 3.8 | L Corticospinal tract |
|  |  | 2 | -34 | -48 | 9E-03 | 9E-05 | 3.7 | R Anterior thalamic radiation |
|  |  | -15 | -29 | -18 | 9E-03 | 2E-04 | 3.6 | L Corticospinal tract |
| *Lower WM volume with use (SU < HC): Tobacco* | | | | | | | | |
| 1 | 75544 | 6 | -26 | -2 | 2E-02 | 3E-08 | 5.4 | R Anterior thalamic radiation |
|  |  | 8 | -56 | 8 | 1E-02 | 2E-06 | 4.6 | R Optic radiation |
|  |  | 8 | -32 | 10 | 1E-02 | 3E-06 | 4.5 | R Corpus callosum |
|  |  | 8 | -60 | 18 | 1E-02 | 5E-06 | 4.4 | R Optic radiation |
|  |  | 12 | -54 | 24 | 1E-02 | 1E-05 | 4.2 | R Cingulum |
|  |  | -6 | -38 | -12 | 1E-02 | 4E-05 | 3.9 | L Anterior thalamic radiation |
|  |  | -12 | -28 | 8 | 1E-02 | 5E-05 | 3.9 | L Fornix |
|  |  | -4 | -26 | -2 | 1E-02 | 6E-05 | 3.9 | L Anterior thalamic radiation |
|  |  | -2 | -30 | 12 | 1E-02 | 7E-05 | 3.8 | L Corpus callosum |
|  |  | 6 | -22 | 12 | 1E-02 | 7E-05 | 3.8 | R Anterior thalamic radiation |
|  |  | -16 | -42 | 2 | 1E-02 | 1E-04 | 3.6 | L Corpus callosum |
|  |  | -4 | -16 | 18 | 1E-02 | 1E-04 | 3.6 | L Fornix |
| *Lower WM volume with use (SU < HC): Cocaine* | | | | | | | | |
| 1 | 24528 | 12 | 12 | -3 | 1E-02 | 2E-05 | 4.1 | R Anterior thalamic radiation |
|  |  | 7 | 33 | -15 | 9E-03 | 9E-05 | 3.8 | R Cingulum |
| 2 | 12408 | 46 | -26 | 12 | 1E-02 | 4E-06 | 4.4 | R Acoustic radiation |
| 3 | 12336 | -26 | 18 | -17 | 1E-02 | 2E-05 | 4.1 | L Inferior occipito-frontal fascicle |
| 4 | 12128 | 28 | -74 | -44 | 1E-02 | 8E-06 | 4.3 | R Middle cerebellar peduncle |
| 5 | 12128 | -48 | -25 | -3 | 1E-02 | 5E-05 | 3.9 | L Acoustic radiation |
| 6 | 12128 | -20 | 57 | -3 | 1E-02 | 5E-05 | 3.9 | L Inferior occipito-frontal fascicle |
| *Lower WM volume with use (SU < HC): Cannabis* | | | | | | | | |
| - | - | - | - | - | - | - | - | - |
| *Lower WM volume with use (SU < HC): Opioids* | | | | | | | | |
| - | - | - | - | - | - | - | - | - |
| *Lower WM volume with use (SU < HC): Stimulants* | | | | | | | | |
| - | - | - | - | - | - | - | - | - |
| *Lower WM volume with use (SU < HC): Polysubstance* | | | | | | | | |
| - | - | - | - | - | - | - | - | - |
| *Higher WM volume with use (HC < SU): Alcohol* | | | | | | | | |
| - | - | - | - | - | - | - | - | - |
| *Higher WM volume with use (HC < SU): Tobacco* | | | | | | | | |
| - | - | - | - | - | - | - | - | - |
| *Higher WM volume with use (HC < SU): Cocaine* | | | | | | | | |
| - | - | - | - | - | - | - | - | - |
| *Higher WM volume with use (HC < SU): Cannabis* | | | | | | | | |
| - | - | - | - | - | - | - | - | - |
| *Higher WM volume with use (HC < SU): Opioids* | | | | | | | | |
| - | - | - | - | - | - | - | - | - |
| *Higher WM volume with use (HC < SU): Stimulants* | | | | | | | | |
| - | - | - | - | - | - | - | - | - |
| *Higher WM volume with use (HC < SU): Polysubstance* | | | | | | | | |
| - | - | - | - | - | - | - | - | - |
| GM, grey matter; WM, white matter; SU, substance user; HC, healthy control; ALE, anatomic likelihood estimation; P, p-value; Z, peak z-value; R, right; L, left. | | | | | | | | |

| **Supplementary Table 6. Contrast analysis of type of substance subgroup** **anatomic likelihood estimation meta-analytic results for studies comparing brain morphological changes between SU and HC, at cluster level inference p < 0.05 (FWE).** | | | | | | | | |
| --- | --- | --- | --- | --- | --- | --- | --- | --- |
| Cluster number | Volume (mm^3^) | MNI coordinates | | | ALE | P | Z | Label (Side, region) |
|  |  | x | y | z |  |  |  |  |
| *Lower GM volume with use (SU < HC): Alcohol + Tobacco* | | | | | | | | |
| - | - | - | - | - | - | - | - | - |
| *Lower GM volume with use (SU < HC): Alcohol - Tobacco* | | | | | | | | |
| 1 | 8344 | -3.3 | 24 | 46 | - | 1E-04 | 3.7 | L Medial Frontal Gyrus BA6 |
|  |  | 0.7 | 19.5 | 46.9 | - | 1E-04 | 3.7 | L Medial Frontal Gyrus BA6 |
|  |  | 0 | 18 | 40 | - | 2E-04 | 3.5 | L Cingulate Gyrus BA32 |
|  |  | -3 | 36 | 36 | - | 3E-04 | 3.4 | L Medial Frontal Gyrus BA8 |
|  |  | 1.6 | 32.4 | 38.4 | - | 7E-04 | 3.2 | L Medial Frontal Gyrus BA8 |
|  |  | 4 | 25 | 38 | - | 9E-04 | 3.1 | R Cingulate Gyrus BA32 |
|  |  | 8 | 28 | 34 | - | 7E-04 | 3.2 | R Cingulate Gyrus BA32 |
|  |  | -4 | 28 | 34 | - | 1E-03 | 3.0 | L Cingulate Gyrus BA32 |
|  |  | 1 | 22 | 34 | - | 1E-03 | 3.0 | L Cingulate Gyrus BA32 |
|  |  | 7 | 36 | 24 | - | 2E-03 | 2.8 | R Cingulate Gyrus BA32 |
|  |  | 4 | 32 | 28 | - | 4E-03 | 2.6 | R Cingulate Gyrus BA32 |
|  |  | -14 | 44 | 6 | - | 6E-03 | 2.5 | L Anterior Cingulate BA32 |
|  |  | 5 | 48 | 14 | - | 1E-02 | 2.2 | R Medial Frontal Gyrus BA9 |
|  |  | -2 | 50 | 10 | - | 1E-02 | 2.2 | L Medial Frontal Gyrus BA9 |
|  |  | -2 | 44 | 12 | - | 2E-02 | 2.2 | L Anterior Cingulate BA32 |
|  |  | -6 | 52 | 4 | - | 2E-02 | 2.1 | L Medial Frontal Gyrus BA10 |
|  |  | 0 | 8 | 45.5 | - | 2E-02 | 2.1 | L Cingulate Gyrus BA24 |
|  |  | -4.5 | 46.5 | 6.5 | - | 2E-02 | 2.1 | L Anterior Cingulate BA32 |
|  |  | 10 | 46 | 12 | - | 2E-02 | 2.0 | R Anterior Cingulate BA32 |
|  |  | -6 | 6 | 44 | - | 4E-02 | 1.7 | L Cingulate Gyrus BA24 |
|  |  | 1 | 5.7 | 52.3 | - | 4E-02 | 1.7 | L Medial Frontal Gyrus BA6 |
|  |  | -3 | 2 | 52 | - | 5E-02 | 1.7 | L Medial Frontal Gyrus BA6 |
| 2 | 2448 | -52 | -14 | 18 | - | 5E-03 | 2.6 | L Postcentral Gyrus BA43 |
|  |  | -52 | -10 | 8 | - | 9E-03 | 2.4 | L Precentral Gyrus BA13 |
|  |  | -50 | -24 | 18 | - | 1E-02 | 2.3 | L Insula BA40 |
|  |  | -38 | -24 | 16 | - | 1E-02 | 2.3 | L Insula BA13 |
|  |  | -34 | -24 | 18 | - | 1E-02 | 2.2 | L Insula BA13 |
|  |  | -35 | -20 | 18 | - | 2E-02 | 2.2 | L Insula BA13 |
|  |  | -42 | -16 | 16 | - | 2E-02 | 2.2 | L Insula BA13 |
|  |  | -30 | -24 | 14 | - | 2E-02 | 2.1 | L Insula BA13 |
|  |  | -48 | -18 | 12 | - | 2E-02 | 2.1 | L Transverse Temporal Gyrus |
|  |  | -42 | -24 | 10 | - | 2E-02 | 2.1 | L Transverse Temporal Gyrus |
|  |  | -52 | -28 | 18 | - | 2E-02 | 2.0 | L Superior Temporal Gyrus |
|  |  | -42 | -10 | 14 | - | 2E-02 | 2.0 | L Insula BA13 |
|  |  | -36 | -20 | 10 | - | 3E-02 | 1.8 | L Claustrum |
| 3 | 1624 | 18 | -42 | -8 | - | 3E-03 | 2.7 | R Culmen |
|  |  | 15 | -36 | 1 | - | 5E-03 | 2.6 | R Parahippocampal Gyrus |
|  |  | 12 | -39.2 | -7.6 | - | 1E-02 | 2.2 | R Culmen |
| 4 | 704 | 46 | -20 | 10 | - | 6E-03 | 2.5 | R Insula BA13 |
|  |  | 42.5 | -17 | 14.5 | - | 8E-03 | 2.4 | R Insula BA13 |
|  |  | 36 | -18 | 14 | - | 2E-02 | 2.1 | R Claustrum |
| 5 | 2768 | 7 | -71 | 9 | - | 2E-02 | 2.0 | R Cuneus BA30 |
|  |  | 15.3 | -68.7 | 10.7 | - | 2E-02 | 2.0 | R Posterior Cingulate Cortex BA30 |
|  |  | 16 | -71 | 16 | - | 2E-02 | 2.0 | R Cuneus BA30 |
|  |  | 5.2 | -60.4 | 6.8 | - | 5E-02 | 1.7 | R Posterior Cingulate Cortex BA30 |
|  |  | 19.7 | -75.7 | 8 | - | 3E-02 | 1.9 | R Lingual Gyrus BA18 |
|  |  | 2.7 | -62.7 | 4 | - | 4E-02 | 1.7 | R Culmen of Vermis |
|  |  | 14 | -64 | 4 | - | 5E-02 | 1.7 | R Lingual Gyrus BA18 |
|  |  | 6 | -60 | 16 | - | 5E-02 | 1.7 | R Posterior Cingulate Cortex BA30 |
| 6 | 1184 | -2 | -28 | -6 | - | 2E-02 | 2.0 | L Brainstem. |
|  |  | 0 | -26 | 1 | - | 2E-02 | 2.0 | L Thalamus |
|  |  | 0 | -22 | 0 | - | 2E-02 | 2.0 | L Thalamus |
|  |  | 1.3 | -22 | 4.7 | - | 2E-02 | 2.0 | L Thalamus |
|  |  | -4 | -34 | -4 | - | 2E-02 | 2.0 | L Culmen |
|  |  | -8 | -29 | -4 | - | 2E-02 | 2.0 | L Thalamus |
| 7 | 1112 | -12 | 18 | 14 | - | 3E-02 | 1.9 | L Caudate |
|  |  | -1.3 | 19.3 | 14 | - | 3E-02 | 1.9 | L Caudate |
|  |  | -10 | 22 | 14 | - | 3E-02 | 1.9 | L Caudate |
|  |  | -5.2 | 21.8 | 10.1 | - | 3E-02 | 1.9 | L Caudate |
| 8 | 792 | 0.6 | -57 | 1.9 | - | 3E-02 | 1.9 | L Culmen |
|  |  | -3 | -54.7 | 7.3 | - | 4E-02 | 1.8 | R Posterior Cingulate Cortex BA30 |
|  |  | -4.9 | -58.4 | 0 | - | 4E-02 | 1.7 | L Culmen |
|  |  | -1 | -54.3 | -4.3 | - | 5E-02 | 1.7 | L Culmen |
|  |  | -8 | -54 | 9 | - | 5E-02 | 1.7 | R Posterior Cingulate Cortex BA30 |
| 9 | 160 | -6 | -56 | 16 | - | 3E-02 | 1.9 | R Posterior Cingulate Cortex BA23 |
|  |  | -10 | -56 | 18 | - | 3E-02 | 1.9 | R Posterior Cingulate Cortex BA30 |
|  |  | -4 | -60 | 20 | - | 4E-02 | 1.7 | R Posterior Cingulate Cortex BA23 |
|  |  | -10 | -62 | 22 | - | 4E-02 | 1.7 | L Precuneus BA 31 |
| *Lower GM volume with use (SU < HC): Alcohol + Cocaine* | | | | | | | | |
| - | - | - | - | - | - | - | - | - |
| *Lower GM volume with use (SU < HC): Alcohol - Cocaine* | | | | | | | | |
| 1 | 8096 | 1 | 23.5 | 45.5 | - | 3E-03 | 2.8 | L Medial Frontal Gyrus BA6 |
|  |  | -6 | 34 | 32 | - | 3E-03 | 2.8 | L Medial Frontal Gyrus BA6 |
|  |  | -0.3 | 20.1 | 41.6 | - | 3E-03 | 2.8 | L Cingulate Gyrus BA32 |
|  |  | -2 | 24.5 | 39 | - | 7E-03 | 2.4 | L Cingulate Gyrus BA32 |
|  |  | -8 | 21 | 31 | - | 4E-03 | 2.7 | L Cingulate Gyrus BA32 |
|  |  | -4 | 28 | 32 | - | 4E-03 | 2.7 | L Cingulate Gyrus BA32 |
|  |  | -0.5 | 7.7 | 45.9 | - | 5E-03 | 2.6 | L Cingulate Gyrus BA24 |
|  |  | -2 | 38 | 36 | - | 5E-03 | 2.6 | L Medial Frontal Gyrus BA8 |
|  |  | 1.2 | 17 | 50 | - | 7E-03 | 2.5 | L Superior Frontal Gyrus BA6 |
|  |  | -0.8 | 19 | 31 | - | 7E-03 | 2.4 | L Cingulate Gyrus BA24 |
|  |  | 0 | 29 | 27 | - | 7E-03 | 2.4 | L Cingulate Gyrus BA32 |
|  |  | 6 | 32 | 29.3 | - | 8E-03 | 2.4 | R Cingulate Gyrus BA32 |
|  |  | -2 | 23.5 | 28 | - | 8E-03 | 2.4 | L Cingulate Gyrus BA32 |
|  |  | 4 | 29 | 36.6 | - | 8E-03 | 2.4 | R Cingulate Gyrus BA32 |
|  |  | -2 | 32 | 24 | - | 1E-02 | 2.3 | L Cingulate Gyrus BA32 |
|  |  | -1 | 2 | 42.5 | - | 1E-02 | 2.3 | L Cingulate Gyrus BA24 |
|  |  | 2 | 12 | 54 | - | 1E-02 | 2.2 | L Superior Frontal Gyrus BA6 |
|  |  | 10 | 35 | 24 | - | 2E-02 | 2.2 | R Anterior Cingulate BA32 |
|  |  | 4 | 22 | 28 | - | 2E-02 | 2.1 | R Cingulate Gyrus BA32 |
|  |  | 2.8 | 34.3 | 21.8 | - | 2E-02 | 2.1 | L Cingulate Gyrus BA32 |
|  |  | 2.6 | 6.3 | 52.6 | - | 3E-02 | 2.0 | R Medial Frontal Gyrus BA6 |
|  |  | 2.8 | -2.2 | 52.3 | - | 3E-02 | 1.9 | R Medial Frontal Gyrus BA6 |
|  |  | 8 | 34 | 18 | - | 3E-02 | 1.9 | R Anterior Cingulate BA32 |
|  |  | 4.7 | 38.7 | 18.7 | - | 3E-02 | 1.8 | R Anterior Cingulate BA32 |
|  |  | 2.4 | 1.8 | 57.5 | - | 5E-02 | 1.7 | L Medial Frontal Gyrus BA6 |
| 2 | 264 | 52 | -14 | -10 | - | 8E-03 | 2.4 | R Superior Temporal Gyrus BA22 |
|  |  | 56 | -16 | -14 | - | 2E-02 | 2.1 | R Superior Temporal Gyrus BA22 |
|  |  | 59 | -16 | -10.5 | - | 3E-02 | 1.9 | R Middle Temporal Gyrus BA21 |
| 3 | 240 | -50 | -26 | 20 | - | 2E-02 | 2.2 | L Insula BA40 |
|  |  | -54 | -15 | 18 | - | 3E-02 | 1.9 | L Postcentral Gyrus BA43 |
|  |  | -56 | -14 | 14 | - | 4E-02 | 1.8 | L Postcentral Gyrus BA43 |
|  |  | -50 | -18.7 | 18 | - | 4E-02 | 1.7 | L Insula BA13 |
| 4 | 288 | 31.4 | 17.3 | -17.1 | - | 3E-02 | 1.8 | R Inferior Frontal Gyrus BA47 |
| *Lower GM volume with use (SU < HC): Alcohol + Cannabis* | | | | | | | | |
| - | - | - | - | - | - | - | - | - |
| *Lower GM volume with use (SU < HC): Alcohol - Cannabis* | | | | | | | | |
| 1 | 9160 | -6 | 36 | 28 | - | 1E-03 | 3.0 | L Medial Frontal Gyrus BA9 |
|  |  | -2.4 | 37 | 32.2 | - | 4E-03 | 2.6 | L Medial Frontal Gyrus BA9 |
|  |  | 0 | 41.6 | 19.2 | - | 8E-03 | 2.4 | L Anterior Cingulate Cortex BA32 |
|  |  | 1 | 23.5 | 45.5 | - | 2E-02 | 2.2 | L Medial Frontal Gyrus BA6 |
|  |  | -14 | 36 | 18 | - | 8E-03 | 2.4 | L Anterior Cingulate Cortex BA32 |
| *Lower GM volume with use (SU < HC): Alcohol + Opioids* | | | | | | | | |
| 1 | 1160 | -4 | 38 | 30 | 9E-03 | - | - | L Medial Frontal Gyrus BA9 |
|  |  | 6 | 36 | 34 | 1E-03 | - | - | R Medial Frontal Gyrus BA6 |
|  |  | 8 | 32 | 36 | 9E-04 | - | - | R Medial Frontal Gyrus BA8 |
| *Lower GM volume with use (SU < HC): Alcohol - Opioids* | | | | | | | | |
| 1 | 1184 | 18.8 | 56.6 | 33.8 | - | 2E-02 | 2.1 | R Superior Frontal Gyrus BA8 |
|  |  | 33 | 48 | 32 | - | 2E-02 | 2.0 | R Middle Frontal Gyrus BA9 |
|  |  | 38 | 52 | 34 | - | 3E-02 | 1.9 | R Middle Frontal Gyrus BA9 |
|  |  | 22.2 | 56.3 | 38.4 | - | 3E-02 | 1.9 | R Superior Frontal Gyrus BA8 |
|  |  | 29 | 51 | 35 | - | 3E-02 | 1.8 | R Superior Frontal Gyrus BA8 |
|  |  | 27.5 | 52 | 40 | - | 4E-02 | 1.7 | R Superior Frontal Gyrus BA8 |
| 2 | 216 | -21.6 | 21.8 | -33.6 | - | 2E-02 | 2.1 | L Inferior Frontal Gyrus BA47 |
|  |  | -22 | 16 | -34 | - | 3E-02 | 1.9 | L Inferior Frontal Gyrus BA47 |
| 3 | 160 | -2 | 28 | 44 | - | 3E-02 | 1.8 | L Medial Frontal Gyrus BA8 |
|  |  | 1 | 30 | 40 | - | 5E-02 | 1.7 | L Medial Frontal Gyrus BA8 |
| 4 | 128 | 4 | 24 | 42 | - | 3E-02 | 1.8 | R Cingulate Gyrus BA32 |
|  |  | 4 | 20 | 40 | - | 4E-02 | 1.7 | R Cingulate Gyrus BA32 |
|  |  | 3 | 20 | 44 | - | 4E-02 | 1.7 | L Cingulate Gyrus BA32 |
| *Lower GM volume with use (SU < HC): Alcohol + Stimulants* | | | | | | | | |
| 1 | 2944 | 6 | 48 | -2 | 9E-03 | - | - | R Anterior Cingulate BA32 |
|  |  | 2 | 50 | 16 | 2E-03 | - | - | L Medial Frontal Gyrus BA9 |
| 2 | 1072 | 0 | 12 | 54 | 3E-03 | - | - | L Superior Frontal Gyrus BA6 |
|  |  | 0 | 6 | 56 | 2E-03 | - | - | L Medial Frontal Gyrus BA6 |
| 3 | 200 | -2 | 40 | 28 | 2E-04 | - | - | L Medial Frontal Gyrus BA9 |
| *Lower GM volume with use (SU < HC): Alcohol - Stimulants* | | | | | | | | |
| 1 | 9032 | 6 | 60 | 4 | - | 2E-02 | 2.1 | R Medial Frontal Gyrus BA10 |
|  |  | -6 | 54 | -8 | - | 3E-02 | 1.9 | L Medial Frontal Gyrus BA10 |
|  |  | -2.4 | 68.3 | -6.8 | - | 3E-02 | 1.9 | L Medial Frontal Gyrus BA10 |
|  |  | 4.6 | 65.2 | -1.2 | - | 4E-02 | 1.7 | R Medial Frontal Gyrus BA10 |
|  |  | -11.9 | 60.2 | -6.6 | - | 5E-02 | 1.7 | L Medial Frontal Gyrus BA10 |
| 2 | 4720 | 5.7 | 55.8 | 25.6 | - | 2E-02 | 2.1 | R Superior Frontal Gyrus BA9 |
|  |  | 6.3 | 65.9 | 18.7 | - | 3E-02 | 1.9 | R Superior Frontal Gyrus BA10 |
|  |  | 0.6 | 53.9 | 33.5 | - | 3E-02 | 1.8 | L Medial Frontal Gyrus BA6 |
| *Lower GM volume with use (SU < HC): Alcohol + Polysubstance* | | | | | | | | |
| - | - | - | - | - | - | - | - | - |
| *Lower GM volume with use (SU < HC): Alcohol - Polysubstance* | | | | | | | | |
| 1 | 600 | -6 | 38 | 30 | - | 3E-03 | 2.7 | L Medial Frontal Gyrus BA9 |
|  |  | -2 | 40 | 32 | - | 5E-03 | 2.5 | L Medial Frontal Gyrus BA6 |
|  |  | -2 | 32 | 24 | - | 4E-02 | 1.8 | L Cingulate Gyrus BA32 |
| 2 | 264 | -16 | 36 | 22 | - | 2E-02 | 2.0 | L Medial Frontal Gyrus BA9 |
|  |  | -16 | 40 | 20 | - | 3E-02 | 1.9 | L Anterior Cingulate BA32 |
|  |  | -20 | 34 | 16 | - | 4E-02 | 1.7 | L Anterior Cingulate BA32 |
| 3 | 280 | -10 | 11 | -4 | - | 3E-03 | 2.8 | L Caudate Head |
| 4 | 128 | -0.5 | -9.5 | 5.5 | - | 5E-02 | 1.7 | L Thalamus |
| *Lower GM volume with use (SU < HC): Tobacco + Cocaine* | | | | | | | | |
| - | - | - | - | - | - | - | - | - |
| *Lower GM volume with use (SU < HC): Tobacco - Cocaine* | | | | | | | | |
| 1 | 136 | 4 | -21 | 6 | - | 3E-02 | 1.9 | R Thalamus MDN |
| *Lower GM volume with use (SU < HC): Tobacco + Cannabis* | | | | | | | | |
| 1 | 3192 | -46 | -8 | -10 | 6E-03 | - | - | L Insula |
|  |  | -50 | 6 | -12 | 4E-03 | - | - | L Superior temporal gyrus |
|  |  | -40 | 10 | -8 | 4E-03 | - | - | L Insula |
| *Lower GM volume with use (SU < HC): Tobacco - Cannabis* | | | | | | | | |
| - | - | 12.6 | -13.4 | -7.6 | - | 4E-02 | 1.7 | R Subthalamic nucleus |
| *Lower GM volume with use (SU < HC): Tobacco + Opioids* | | | | | | | | |
| 1 | 2152 | -6.6 | -34.8 | -0.3 | - | 4E-02 | 1.7 | L Culmen |
| 2 | 496 | -16.5 | -36.3 | -2.5 | - | 4E-02 | 1.8 | L Parahippocampal Gyrus |
|  |  | -6.5 | -30.1 | -3.9 | - | 4E-02 | 1.7 | L Thalamus |
| 3 | 328 | 5.4 | -18.5 | 3.1 | - | 4E-02 | 1.7 | R Thalamus |
| 4 | 240 | -9.5 | -21.9 | 3.6 | - | 4E-02 | 1.8 | L Thalamus |
| 4 | 240 | -7.8 | -46 | -1.6 | - | 4E-02 | 1.8 | L Culmen |
| *Lower GM volume with use (SU < HC): Tobacco - Opioids* | | | | | | | | |
| 1 | 2152 | 48.9 | -18.7 | 15.9 | - | 2E-02 | 2.2 | R Insula BA13 |
| 2 | 496 | 44.7 | 40 | 23 | - | 2E-02 | 2.1 | R Middle Frontal Gyrus BA9 |
|  |  | 38 | 36 | 27.3 | - | 3E-02 | 1.9 | R Middle Frontal Gyrus BA9 |
| 3 | 328 | 49.1 | 16.6 | 19.3 | - | 2E-02 | 2.1 | R Inferior Frontal Gyrus BA9 |
| 4 | 240 | 26.5 | 54.4 | 36 | - | 3E-02 | 1.9 | R Superior Frontal Gyrus BA8 |
| 5 | 1816 | -6.6 | -34.8 | -0.3 | - | 4E-02 | 1.7 | L Culmen |
|  |  | -16.5 | -36.3 | -2.5 | - | 4E-02 | 1.8 | L Parahippocampal Gyrus |
|  |  | -6.5 | -30.1 | -3.9 | - | 4E-02 | 1.7 | L Thalamus |
| 6 | 840 | 5.4 | -18.5 | 3.1 | - | 4E-02 | 1.7 | R Thalamus |
| 7 | 328 | -9.5 | -21.9 | 3.6 | - | 4E-02 | 1.8 | L Thalamus |
| 8 | 144 | -7.8 | -46 | -1.6 | - | 4E-02 | 1.8 | L Culmen |
| *Lower GM volume with use (SU < HC): Tobacco + Stimulants* | | | | | | | | |
| - | - | - | - | - | - | - | - | - |
| *Lower GM volume with use (SU < HC): Tobacco - Stimulants* | | | | | | | | |
| 1 | 17160 | 2.3 | 53.9 | 9.5 | - | 2E-02 | 2.1 | R Medial frontal gyrus |
|  |  | -1.3 | 54.1 | 25.2 | - | 2E-02 | 2.1 | L Medial frontal gyrus |
|  |  | 5.7 | 50.8 | -0.8 | - | 3E-02 | 1.9 | R Anterior cingulate cortex |
|  |  | -4.4 | 55.5 | 14.4 | - | 3E-02 | 1.8 | L Medial frontal gyrus |
|  |  | 1.4 | 52.7 | 35.3 | - | 3E-02 | 1.8 | L Medial frontal gyrus |
|  |  | 2 | 66.3 | -1.9 | - | 4E-02 | 1.8 | R Medial frontal gyrus |
|  |  | -5.5 | 40.8 | 30.4 | - | 5E-02 | 1.7 | L Medial frontal gyrus |
|  |  | -2.6 | 47.1 | 38 | - | 5E-02 | 1.7 | L Medial frontal gyrus |
|  |  | 10.7 | 49.3 | -13.3 | - | 5E-02 | 1.7 | R Anterior cingulate cortex |
|  |  | 0 | 42.6 | 0 | - | 5E-02 | 1.7 | L Anterior cingulate cortex |
|  |  | 1.2 | 57.6 | -21.8 | - | 5E-02 | 1.7 | L Medial frontal gyrus |
|  |  | 10.2 | 62.6 | 15.2 | - | 5E-02 | 1.7 | R Superior frontal gyrus |
| *Lower GM volume with use (SU < HC): Tobacco + Polysubstance* | | | | | | | | |
| 1 | 2616 | -6 | -20 | -2 | 8E-03 | - | - | L Thalamus |
|  |  | 2 | -18 | 4 | 8E-03 | - | - | L Thalamus MDN |
|  |  | 4 | -16 | 10 | 4E-03 | - | - | R Thalamus MDN |
|  |  | 4 | -14 | 20 | 2E-03 | - | - | R Thalamus |
| 2 | 472 | -6 | 10 | 4 | 5E-03 | - | - | L Caudate Body |
| *Lower GM volume with use (SU < HC): Tobacco - Polysubstance* | | | | | | | | |
| 1 | 2320 | -7 | 8 | -6 | - | 5E-03 | 2.6 | L Caudate |
|  |  | -6 | -4 | -6 | - | 1E-02 | 2.3 | L Hypothalamus |
|  |  | -2 | -12 | 12 | - | 2E-02 | 2.1 | L Thalamus |
|  |  | 0 | -2 | -2 | - | 2E-02 | 2.0 | L Thalamus |
|  |  | -8 | 2 | 0 | - | 2E-02 | 2.0 | L Medial globus pallidus |
|  |  | -1 | 4 | -1.5 | - | 3E-02 | 2.0 | L Caudate |
|  |  | -4 | 2 | 6 | - | 3E-02 | 1.9 | L Caudate |
|  |  | 6 | 6 | 2 | - | 3E-02 | 1.9 | R Caudate |
|  |  | 1 | -8 | 7 | - | 3E-01 | 0.0 | L Thalamus |
|  |  | -4 | -6 | 4 | - | 3E-02 | 1.9 | L Thalamus |
|  |  | 4 | 9.5 | -2.5 | - | 4E-02 | 1.7 | R Caudate |
|  |  | -4.4 | -11.8 | 3.6 | - | 4E-02 | 1.7 | L Thalamus / Medial dorsal nucleus |
|  |  | -6.5 | -10 | -1.5 | - | 5E-02 | 1.7 | L Thalamus |
| 2 | 312 | -2 | -11.3 | 16.7 | - | 3E-02 | 2.0 | L Thalamus |
|  |  | 2 | -12.8 | 15.6 | - | 3E-02 | 1.8 | L Thalamus |
|  |  | 6 | -14 | 20 | - | 4E-02 | 1.7 | R Thalamus |
| *Lower GM volume with use (SU < HC): Cocaine + Cannabis* | | | | | | | | |
| - | - | - | - | - | - | - | - | - |
| *Lower GM volume with use (SU < HC): Cocaine - Cannabis* | | | | | | | | |
| - | - | - | - | - | - | - | - | - |
| *Lower GM volume with use (SU < HC): Cocaine + Opioids* | | | | | | | | |
| - | - | - | - | - | - | - | - | - |
| *Lower GM volume with use (SU < HC): Cocaine - Opioids* | | | | | | | | |
| - | - | - | - | - | - | - | - | - |
| *Lower GM volume with use (SU < HC): Cocaine + Stimulants* | | | | | | | | |
| - | - | - | - | - | - | - | - | - |
| *Lower GM volume with use (SU < HC): Cocaine - Stimulants* | | | | | | | | |
| - | - | - | - | - | - | - | - | - |
| *Lower GM volume with use (SU < HC): Cocaine + Polysubstance* | | | | | | | | |
| - | - | - | - | - | - | - | - | - |
| *Lower GM volume with use (SU < HC): Cocaine - Polysubstance* | | | | | | | | |
| - | - | - | - | - | - | - | - | - |
| *Lower GM volume with use (SU < HC): Cannabis + Opioids* | | | | | | | | |
| 1 | 1304 | -26 | 16 | -16 | 4E-03 | - | - | L Inferior frontal gyrus |
|  |  | -33 | 14 | -13 | 1E-03 | - | - | L Insula |
|  |  | -18 | 18 | -20 | 8E-04 | - | - | L Subcallosal gyrus |
| 2 | 56 | -17 | 9 | -18 | 6E-04 | - | - | L Orbitofrontal cortex |
| 3 | 8 | -22 | 14 | -24 | 3E-04 | - | - | L Inferior frontal gyrus |
| 4 | 8 | -18 | 10 | -22 | 3E-04 | - | - | L Inferior frontal gyrus |
| 5 | 8 | -12 | 14 | -14 | 3E-04 | - | - | L Subcallosal gyrus |
| *Lower GM volume with use (SU < HC): Cannabis - Opioids* | | | | | | | | |
| 1 | 168 | 32.3 | 49.3 | 31.2 | - | 5E-02 | 1.7 | R Middle frontal gyrus |
| 2 | 136 | 15.8 | 43.3 | 33.1 | - | 5E-02 | 1.7 | R Medial frontal gyrus |
| *Lower GM volume with use (SU < HC): Cannabis + Stimulants* | | | | | | | | |
| 1 | 352 | -52 | -6 | -4 | 4E-04 | - | - | L Superior temporal gyrus |
| *Lower GM volume with use (SU < HC): Cannabis - Stimulants* | | | | | | | | |
| - | - | - | - | - | - | - | - | - |
| *Lower GM volume with use (SU < HC): Cannabis + Polysubstance* | | | | | | | | |
| 1 | 8264 | 2 | -16 | 6 | 1E-02 | - | - | L Thalamus / Medial dorsal nucleus |
|  |  | -4 | -8 | 0 | 9E-03 | - | - | L Thalamus |
|  |  | 6 | -4 | -4 | 7E-03 | - | - | R Thalamus |
| 2 | 96 | -56 | 6 | -10 | 8E-04 | - | - | L Superior temporal gyrus |
|  |  | -54 | 0 | -8 | 5E-04 | - | - | L Superior temporal gyrus |
| 3 | 64 | -60 | -2 | -22 | 8E-04 | - | - | L Middle temporal gyrus |
| 4 | 8 | -62 | -4 | -24 | 4E-04 | - | - | L Middle temporal gyrus |
| 5 | 8 | -64 | -2 | -24 | 3E-04 | - | - | L Middle temporal gyrus |
| 6 | 8 | -62 | 2 | -20 | 3E-04 | - | - | L Middle temporal gyrus |
| 7 | 8 | -56 | 0 | -10 | 3E-04 | - | - | L Superior temporal gyrus |
| 8 | 8 | -56 | -2 | -8 | 3E-04 | - | - | L Superior temporal gyrus |
| 9 | 8 | -58 | 10 | -8 | 3E-04 | - | - | L Superior temporal gyrus |
| 10 | 8 | -54 | -2 | -6 | 4E-04 | - | - | L Superior temporal gyrus |
| 11 | 8 | -52 | 4 | -6 | 3E-04 | - | - | L Superior temporal gyrus |
| 12 | 8 | -56 | 10 | -6 | 3E-04 | - | - | L Superior temporal gyrus |
| *Lower GM volume with use (SU < HC): Cannabis - Polysubstance* | | | | | | | | |
| 1 | 168 | -6.4 | 14.9 | 2.5 | - | 3E-02 | 1.9 | L Caudate |
| *Lower GM volume with use (SU < HC): Opioids + Stimulants* | | | | | | | | |
| 1 | 1112 | -2 | 44 | 30 | 2E-03 | - | - | L Medial frontal gyrus |
| 2 | 16 | -4 | 40 | -30 | 8E-05 | - | - | L Orbitofrontal gyrus |
| 3 | 8 | -6 | 42 | -32 | 4E-05 | - | - | L Orbitofrontal gyrus |
| 4 | 8 | -6 | 38 | -28 | 4E-05 | - | - | L Medial frontal gyrus |
| 5 | 8 | 6 | 42 | 32 | 5E-05 | - | - | R Medial frontal gyrus |
| 6 | 8 | 8 | 44 | 32 | 4E-05 | - | - | R Medial frontal gyrus |
| 7 | 8 | 6 | 44 | 34 | 7E-05 | - | - | R Medial frontal gyrus |
| *Lower GM volume with use (SU < HC): Opioids - Stimulants* | | | | | | | | |
| - | - | - | - | - | - | - | - | - |
| *Lower GM volume with use (SU < HC): Opioids + Polysubstance* | | | | | | | | |
| - | - | - | - | - | - | - | - | - |
| *Lower GM volume with use (SU < HC): Opioids - Polysubstance* | | | | | | | | |
| 1 | 1360 | -0.4 | -6.3 | 5.9 | - | 2E-02 | 2.1 | L Thalamus |
| 2 | 192 | -9.6 | 10.2 | 4.8 | - | 2E-02 | 2.0 | L Caudate |
|  |  | -9 | 15 | 1 | - | 4E-02 | 1.8 | L Caudate |
| *Lower GM volume with use (SU < HC): Stimulants + Polysubstance* | | | | | | | | |
| 1 | 3160 | -62 | -6 | 4 | 4E-03 | - | - | L Superior temporal gyrus |
|  |  | -60 | -10 | 4 | 4E-03 | - | - | L Superior temporal gyrus |
|  |  | -60 | -2 | 0 | 2E-03 | - | - | L Superior temporal gyrus |
| *Lower GM volume with use (SU < HC): Stimulants - Polysubstance* | | | | | | | | |
| - | - | - | - | - | - | - | - | - |
| *Higher GM volume with use (HC < SU): Tobacco + Cannabis* | | | | | | | | |
| 1 | 10824 | -26 | -4 | 2 | 1E-02 | - | - | L Putamen |
|  |  | -28 | 10 | -2 | 2E-03 | - | - | L Putamen |
|  |  | -20 | 6 | -10 | 6E-04 | - | - | L Putamen |
| *Higher GM volume with use (HC < SU): Tobacco - Cannabis* | | | | | | | | |
| 1 | 7512 | 28 | -17.8 | 54.3 | - | 3E-02 | 1.8 | R Precentral gyrus |
| 2 | 168 | 0.7 | -16.6 | -7.3 | - | 3E-02 | 1.9 | L Red nucleus |
| *Higher GM volume with use (HC < SU): Tobacco + Polysubstance* | | | | | | | | |
| 1 | 12408 | -26 | -4 | 2 | 1E-02 | - | - | L Putamen |
| 2 | 2472 | -22 | 14 | -10 | 4E-04 | - | - | L Putamen |
|  |  | -22 | 12 | -14 | 4E-04 | - | - | L Putamen |
| 3 | 1552 | 20 | 18 | -6 | 2E-04 | - | - | R Putamen |
| *Higher GM volume with use (HC < SU): Tobacco - Polysubstance* | | | | | | | | |
| - | - | - | - | - | - | - | - | - |
| *Higher GM volume with use (HC < SU): Cannabis + Polysubstance* | | | | | | | | |
| 1 | 7720 | -26 | -4 | 2 | 1E-02 | - | - | L Putamen |
| 2 | 864 | -16 | 10 | -14 | 3E-04 | - | - | L Putamen |
|  |  | -12 | 12 | -10 | 2E-04 | - | - | L Putamen |
| 3 | 200 | -24 | 18 | -6 | 7E-05 | - | - | L Putamen |
| 4 | 8 | -20 | 14 | -2 | 5E-06 | - | - | L Putamen |
| *Higher GM volume with use (HC < SU): Cannabis - Polysubstance* | | | | | | | | |
| - | - | - | - | - | - | - | - | - |
| *WM volume decrease with use (SU < HC): Alcohol + Tobacco* | | | | | | | | |
| 1 | 1512 | -10 | -32 | -14 | 1E-03 | - | - | R Anterior thalamic radiation |
| *WM volume decrease with use (SU < HC): Alcohol - Tobacco* | | | | | | | | |
| - | - | - | - | - | - | - | - | - |
| *WM volume decrease with use (SU < HC): Alcohol + Cocaine* | | | | | | | | |
| - | - | - | - | - | - | - | - | - |
| *WM volume decrease with use (SU < HC): Alcohol - Cocaine* | | | | | | | | |
| - | - | - | - | - | - | - | - | - |
| *WM volume decrease with use (SU < HC): Alcohol + Cannabis* | | | | | | | | |
| - | - | - | - | - | - | - | - | - |
| *WM volume decrease with use (SU < HC): Alcohol - Cannabis* | | | | | | | | |
| - | - | - | - | - | - | - | - | - |
| *WM volume decrease with use (SU < HC): Alcohol + Opioids* | | | | | | | | |
| - | - | - | - | - | - | - | - | - |
| *WM volume decrease with use (SU < HC): Alcohol - Opioids* | | | | | | | | |
| - | - | - | - | - | - | - | - | - |
| *WM volume decrease with use (SU < HC): Alcohol + Stimulants* | | | | | | | | |
| - | - | - | - | - | - | - | - | - |
| *WM volume decrease with use (SU < HC): Alcohol - Stimulants* | | | | | | | | |
| - | - | - | - | - | - | - | - | - |
| *WM volume decrease with use (SU < HC): Alcohol + Polysubstance* | | | | | | | | |
| - | - | - | - | - | - | - | - | - |
| *WM volume decrease with use (SU < HC): Alcohol - Polysubstance* | | | | | | | | |
| - | - | - | - | - | - | - | - | - |
| *WM volume decrease with use (SU < HC): Tobacco + Cocaine* | | | | | | | | |
| - | - | - | - | - | - | - | - | - |
| *WM volume decrease with use (SU < HC): Tobacco - Cocaine* | | | | | | | | |
| - | - | - | - | - | - | - | - | - |
| *WM volume decrease with use (SU < HC): Tobacco + Cannabis* | | | | | | | | |
| - | - | - | - | - | - | - | - | - |
| *WM volume decrease with use (SU < HC): Tobacco - Cannabis* | | | | | | | | |
| - | - | - | - | - | - | - | - | - |
| *WM volume decrease with use (SU < HC): Tobacco + Opioids* | | | | | | | | |
| - | - | - | - | - | - | - | - | - |
| *WM volume decrease with use (SU < HC): Tobacco - Opioids* | | | | | | | | |
| - | - | - | - | - | - | - | - | - |
| *WM volume decrease with use (SU < HC): Tobacco + Stimulants* | | | | | | | | |
| - | - | - | - | - | - | - | - | - |
| *WM volume decrease with use (SU < HC): Tobacco - Stimulants* | | | | | | | | |
| - | - | - | - | - | - | - | - | - |
| *WM volume decrease with use (SU < HC): Tobacco + Polysubstance* | | | | | | | | |
| - | - | - | - | - | - | - | - | - |
| *WM volume decrease with use (SU < HC): Tobacco - Polysubstance* | | | | | | | | |
| - | - | - | - | - | - | - | - | - |
| *WM volume decrease with use (SU < HC): Cocaine + Cannabis* | | | | | | | | |
| - | - | - | - | - | - | - | - | - |
| *WM volume decrease with use (SU < HC): Cocaine - Cannabis* | | | | | | | | |
| - | - | - | - | - | - | - | - | - |
| *WM volume decrease with use (SU < HC): Cocaine + Opioids* | | | | | | | | |
| - | - | - | - | - | - | - | - | - |
| *WM volume decrease with use (SU < HC): Cocaine - Opioids* | | | | | | | | |
| - | - | - | - | - | - | - | - | - |
| *WM volume decrease with use (SU < HC): Cocaine + Stimulants* | | | | | | | | |
| - | - | - | - | - | - | - | - | - |
| *WM volume decrease with use (SU < HC): Cocaine - Stimulants* | | | | | | | | |
| - | - | - | - | - | - | - | - | - |
| *WM volume decrease with use (SU < HC): Cocaine + Polysubstance* | | | | | | | | |
| - | - | - | - | - | - | - | - | - |
| *WM volume decrease with use (SU < HC): Cocaine - Polysubstance* | | | | | | | | |
| - | - | - | - | - | - | - | - | - |
| *WM volume decrease with use (SU < HC): Cannabis + Opioids* | | | | | | | | |
| - | - | - | - | - | - | - | - | - |
| *WM volume decrease with use (SU < HC): Cannabis - Opioids* | | | | | | | | |
| - | - | - | - | - | - | - | - | - |
| *WM volume decrease with use (SU < HC): Cannabis + Stimulants* | | | | | | | | |
| - | - | - | - | - | - | - | - | - |
| *WM volume decrease with use (SU < HC): Cannabis - Stimulants* | | | | | | | | |
| - | - | - | - | - | - | - | - | - |
| *WM volume decrease with use (SU < HC): Cannabis + Polysubstance* | | | | | | | | |
| - | - | - | - | - | - | - | - | - |
| *WM volume decrease with use (SU < HC): Cannabis - Polysubstance* | | | | | | | | |
| - | - | - | - | - | - | - | - | - |
| *WM volume decrease with use (SU < HC): Opioids + Stimulants* | | | | | | | | |
| - | - | - | - | - | - | - | - | - |
| *WM volume decrease with use (SU < HC): Opioids - Stimulants* | | | | | | | | |
| - | - | - | - | - | - | - | - | - |
| *WM volume decrease with use (SU < HC): Opioids + Polysubstance* | | | | | | | | |
| - | - | - | - | - | - | - | - | - |
| *WM volume decrease with use (SU < HC): Opioids - Polysubstance* | | | | | | | | |
| - | - | - | - | - | - | - | - | - |
| *WM volume decrease with use (SU < HC): Stimulants + Polysubstance* | | | | | | | | |
| - | - | - | - | - | - | - | - | - |
| *WM volume decrease with use (SU < HC): Stimulants - Polysubstance* | | | | | | | | |
| - | - | - | - | - | - | - | - | - |
| *WM volume increase with use (HC < SU): Tobacco + Cannabis* | | | | | | | | |
| - | - | - | - | - | - | - | - | - |
| *WM volume increase with use (HC < SU): Tobacco - Cannabis* | | | | | | | | |
| - | - | - | - | - | - | - | - | - |
| *WM volume increase with use (HC < SU): Tobacco + Polysubstance* | | | | | | | | |
| - | - | - | - | - | - | - | - | - |
| *WM volume increase with use (HC < SU): Tobacco - Polysubstance* | | | | | | | | |
| - | - | - | - | - | - | - | - | - |
| *WM volume increase with use (HC < SU): Cannabis + Polysubstance* | | | | | | | | |
| - | - | - | - | - | - | - | - | - |
| *WM volume increase with use (HC < SU): Cannabis - Polysubstance* | | | | | | | | |
| - | - | - | - | - | - | - | - | - |
| GM, grey matter; WM, white matter; SU, substance user; HC, healthy control; ALE, anatomic likelihood estimation; P, p-value; Z, peak z-value; R, right; L, left. | | | | | | | | |

| **Supplementary Table 7. Meta-analytic connectivity modeling results from primary outcomes, at cluster level inference p < 0.05 (FWE).** | | | | | | | | |
| --- | --- | --- | --- | --- | --- | --- | --- | --- |
| Cluster number | Volume (mm^3^) | MNI coordinates | | | ALE | P | Z | Label (Side, region) |
|  |  | x | y | z |  |  |  |  |
| *a. Right anterior cingulate cortex: 269 foci, 23 experiments, 332 subjects (x=8, y=48, z=0)* | | | | | | | | |
| 1 | 18040 | 8 | 48 | 0 | 1E-01 | 0E+00 | 15.2 | R Anterior cingulate cortex BA32 |
|  |  | -6 | 48 | -4 | 2E-02 | 9E-07 | 4.8 | L Anterior cingulate cortex BA32 |
|  |  | -26 | 42 | 20 | 8E-03 | 3E-03 | 2.7 | L Middle frontal gyrus BA9 |
|  |  | -22 | 36 | 10 | 8E-03 | 4E-03 | 2.6 | L Anterior cingulate cortex BA32 |
|  |  | 0 | 28 | -18 | 8E-03 | 5E-03 | 2.6 | L Anterior cingulate cortex BA32 |
|  |  | -2 | 42 | 22 | 6E-03 | 2E-02 | 2.0 | L Anterior cingulate cortex BA32 |
| *b. Left thalamus: 1025 foci, 48 experiments, 731 subjects (x=-6, y=-24, z=0)* | | | | | | | | |
| 1 | 111152 | x | y | z | ALE | P | Z | Label (Nearest Gray Matter within 5mm) |
|  |  | -6 | -24 | 0 | 2E-01 | 0E+00 | 20.2 | L Thalamus |
|  |  | 8 | -24 | 0 | 6E-02 | 2E-15 | 7.9 | R Thalamus |
|  |  | 20 | -26 | 2 | 4E-02 | 2E-09 | 5.9 | R Thalamus |
|  |  | 36 | 26 | 2 | 4E-02 | 1E-08 | 5.6 | R Insula BA13 |
|  |  | 8 | -6 | 6 | 4E-02 | 1E-08 | 5.6 | R Thalamus |
|  |  | -32 | 20 | 0 | 4E-02 | 2E-08 | 5.5 | L Claustrum |
|  |  | 10 | -14 | 12 | 3E-02 | 1E-07 | 5.2 | R Thalamus |
|  |  | -26 | -6 | 64 | 3E-02 | 4E-07 | 5.0 | L Middle Frontal Gyrus BA6 |
|  |  | -48 | 10 | 26 | 3E-02 | 2E-06 | 4.6 | L Inferior Frontal Gyrus BA9 |
|  |  | 36 | 14 | -6 | 3E-02 | 3E-06 | 4.5 | R Claustrum |
|  |  | -12 | 0 | 8 | 3E-02 | 5E-06 | 4.4 | L Thalamus |
|  |  | 48 | 6 | 30 | 2E-02 | 2E-05 | 4.1 | R Precentral Gyrus BA6 |
|  |  | -42 | 12 | -10 | 2E-02 | 2E-05 | 4.1 | L Insula BA13 |
|  |  | 22 | 10 | 4 | 2E-02 | 4E-05 | 4.0 | R Lentiform Nucleus |
|  |  | -40 | 0 | 42 | 2E-02 | 4E-05 | 4.0 | L Precentral Gyrus BA6 |
|  |  | 14 | 6 | -4 | 2E-02 | 4E-05 | 3.9 | R Lentiform Nucleus |
|  |  | -10 | 6 | -2 | 2E-02 | 6E-05 | 3.8 | L Lentiform Nucleus |
|  |  | -42 | -4 | 58 | 2E-02 | 6E-05 | 3.8 | L Precentral Gyrus BA6 |
|  |  | 22 | 2 | -6 | 2E-02 | 8E-05 | 3.8 | R Lentiform Nucleus |
|  |  | -42 | -8 | 48 | 2E-02 | 9E-05 | 3.7 | L Precentral Gyrus BA6 |
|  |  | -22 | 8 | 4 | 2E-02 | 1E-04 | 3.7 | L Lentiform Nucleus |
|  |  | -22 | 0 | -12 | 2E-02 | 1E-04 | 3.7 | L Lentiform Nucleus |
|  |  | -28 | -6 | 52 | 2E-02 | 1E-04 | 3.6 | L Precentral Gyrus BA6 |
|  |  | -58 | -16 | 20 | 2E-02 | 3E-04 | 3.5 | L Postcentral Gyrus BA43 |
|  |  | 50 | 38 | 0 | 2E-02 | 4E-04 | 3.4 | R Inferior Frontal Gyrus BA46 |
|  |  | -48 | 4 | 18 | 2E-02 | 4E-04 | 3.4 | L Inferior Frontal Gyrus BA44 |
|  |  | -46 | 28 | 10 | 2E-02 | 5E-04 | 3.3 | L Inferior Frontal Gyrus BA45 |
|  |  | -54 | -42 | 12 | 2E-02 | 7E-04 | 3.2 | L Superior Temporal Gyrus BA22 |
|  |  | 50 | 14 | 4 | 2E-02 | 8E-04 | 3.1 | R Precentral Gyrus BA44 |
|  |  | -54 | -12 | 40 | 2E-02 | 9E-04 | 3.1 | L Postcentral Gyrus BA3 |
|  |  | -60 | -28 | 10 | 2E-02 | 9E-04 | 3.1 | L Superior Temporal Gyrus BA42 |
|  |  | 46 | 32 | 24 | 2E-02 | 1E-03 | 3.1 | R Middle Frontal Gyrus BA9 |
|  |  | 26 | -32 | -8 | 2E-02 | 1E-03 | 3.1 | R Parahippocampal Gyrus BA27 |
|  |  | -56 | -24 | 24 | 2E-02 | 1E-03 | 3.0 | L Postcentral Gyrus BA40 |
|  |  | 20 | -4 | -14 | 2E-02 | 1E-03 | 3.0 | R Parahippocampal Gyrus |
|  |  | -32 | -2 | -16 | 2E-02 | 1E-03 | 3.0 | L Parahippocampal Gyrus |
|  |  | -60 | -12 | 8 | 2E-02 | 2E-03 | 2.9 | L Transverse Temporal Gyrus BA42 |
|  |  | 56 | 10 | 20 | 2E-02 | 2E-03 | 2.9 | R Inferior Frontal Gyrus BA44 |
|  |  | 30 | -18 | -4 | 2E-02 | 2E-03 | 2.9 | R Lentiform Nucleus |
|  |  | 34 | 42 | 28 | 2E-02 | 3E-03 | 2.8 | R Superior Frontal Gyrus BA9 |
|  |  | -12 | 18 | -2 | 2E-02 | 3E-03 | 2.8 | L Caudate |
|  |  | -30 | -20 | 64 | 2E-02 | 3E-03 | 2.8 | L Precentral Gyrus BA4 |
|  |  | -36 | 12 | 32 | 2E-02 | 3E-03 | 2.7 | L Precentral Gyrus BA9 |
|  |  | -16 | -6 | -2 | 2E-02 | 4E-03 | 2.7 | L Lentiform Nucleus |
|  |  | 32 | -4 | -14 | 1E-02 | 6E-03 | 2.5 | R Lentiform Nucleus |
|  |  | -46 | 6 | 4 | 1E-02 | 6E-03 | 2.5 | L Precentral Gyrus BA44 |
|  |  | 42 | 34 | 14 | 1E-02 | 7E-03 | 2.5 | R Middle Frontal Gyrus BA46 |
|  |  | -52 | -4 | 4 | 1E-02 | 8E-03 | 2.4 | L Superior Temporal Gyrus BA22 |
|  |  | -38 | 38 | 2 | 1E-02 | 1E-02 | 2.3 | L Inferior Frontal Gyrus BA46 |
|  |  | 18 | 22 | 0 | 1E-02 | 1E-02 | 2.2 | R Caudate |
|  |  | -54 | 8 | -6 | 1E-02 | 2E-02 | 2.1 | L Superior Temporal Gyrus BA22 |
|  |  | 6 | -14 | -14 | 1E-02 | 2E-02 | 2.0 | R Brainstem |
|  |  | -30 | 8 | -16 | 1E-02 | 3E-02 | 1.9 | L Subcallosal Gyrus BA34 |
|  |  | -38 | 22 | 30 | 1E-02 | 3E-02 | 1.9 | L Middle Frontal Gyrus BA9 |
|  |  | -12 | 10 | 16 | 1E-02 | 4E-02 | 1.8 | L Caudate |
|  |  | -46 | 38 | 0 | 1E-02 | 4E-02 | 1.8 | L Inferior Frontal Gyrus BA46 |
|  |  | -32 | 8 | 52 | 1E-02 | 4E-02 | 1.8 | L Middle Frontal Gyrus BA6 |
| *c. Left putamen: 1076 foci, 49 experiments, 727 subjects (x=-26, y=-4, z=2)* | | | | | | | | |
| 1 | 150424 | -26 | -4 | -2 | 2E-01 | 0E+00 | 19.8 | L Lentiform Nucleus |
|  |  | -12 | -20 | 4 | 6E-02 | 3E-16 | 8.1 | L Thalamus |
|  |  | 24 | 0 | 2 | 6E-02 | 9E-16 | 8.0 | R Lentiform Nucleus |
|  |  | 12 | -18 | 6 | 4E-02 | 1E-10 | 6.3 | R Thalamus |
|  |  | -56 | 6 | 28 | 3E-02 | 4E-08 | 5.3 | L Precentral Gyrus BA6 |
|  |  | -42 | 2 | 4 | 3E-02 | 2E-07 | 5.0 | L Insula BA13 |
|  |  | 56 | -6 | 30 | 3E-02 | 3E-07 | 5.0 | R Precentral Gyrus BA6 |
|  |  | 60 | 4 | 20 | 3E-02 | 3E-07 | 5.0 | R Precentral Gyrus BA6 |
|  |  | 36 | 20 | 2 | 3E-02 | 5E-07 | 4.9 | R Insula BA13 |
|  |  | -48 | -26 | 38 | 3E-02 | 7E-07 | 4.8 | L Postcentral Gyrus BA2 |
|  |  | -38 | -26 | 56 | 3E-02 | 2E-06 | 4.6 | L Postcentral Gyrus BA3 |
|  |  | -60 | -2 | 20 | 3E-02 | 2E-06 | 4.6 | L Precentral Gyrus BA4 |
|  |  | -56 | -6 | 28 | 3E-02 | 4E-06 | 4.5 | L Precentral Gyrus BA6 |
|  |  | -48 | -6 | 46 | 3E-02 | 5E-06 | 4.4 | L Precentral Gyrus BA4 |
|  |  | -50 | -14 | 42 | 3E-02 | 5E-06 | 4.4 | L Postcentral Gyrus BA3 |
|  |  | 50 | -10 | 40 | 3E-02 | 7E-06 | 4.4 | R Precentral Gyrus BA4 |
|  |  | -56 | -12 | 42 | 3E-02 | 7E-06 | 4.3 | L Postcentral Gyrus BA3 |
|  |  | 62 | -32 | 26 | 3E-02 | 7E-06 | 4.3 | R Inferior Parietal Lobule BA40 |
|  |  | 54 | -6 | 40 | 3E-02 | 8E-06 | 4.3 | R Precentral Gyrus BA4 |
|  |  | -40 | -30 | 14 | 3E-02 | 1E-05 | 4.2 | L Superior Temporal Gyrus BA41 |
|  |  | 56 | 8 | 30 | 3E-02 | 1E-05 | 4.2 | R Inferior Frontal Gyrus BA9 |
|  |  | 40 | -6 | 56 | 3E-02 | 2E-05 | 4.2 | R Precentral Gyrus BA6 |
|  |  | 26 | -2 | -18 | 2E-02 | 3E-05 | 4.0 | R Parahippocampal Gyrus |
|  |  | -52 | 12 | -2 | 2E-02 | 3E-05 | 4.0 | L Superior Temporal Gyrus BA22 |
|  |  | 12 | 2 | 8 | 2E-02 | 4E-05 | 3.9 | R Caudate |
|  |  | 66 | -16 | 6 | 2E-02 | 4E-05 | 3.9 | R Transverse Temporal Gyrus BA42 |
|  |  | -36 | -40 | 46 | 2E-02 | 5E-05 | 3.9 | L Inferior Parietal Lobule BA40 |
|  |  | 40 | 8 | -14 | 2E-02 | 7E-05 | 3.8 | R Extra-Nuclear BA13 |
|  |  | -64 | -30 | 14 | 2E-02 | 7E-05 | 3.8 | L Superior Temporal Gyrus BA42 |
|  |  | -40 | -10 | 56 | 2E-02 | 7E-05 | 3.8 | L Precentral Gyrus BA4 |
|  |  | 54 | -24 | 20 | 2E-02 | 1E-04 | 3.7 | R Insula BA13 |
|  |  | -50 | -36 | 14 | 2E-02 | 2E-04 | 3.6 | L Superior Temporal Gyrus BA41 |
|  |  | 42 | 4 | -6 | 2E-02 | 3E-04 | 3.4 | R Claustrum |
|  |  | 26 | -10 | 58 | 2E-02 | 3E-04 | 3.4 | R Sub-Gyral BA6 |
|  |  | -34 | -22 | 6 | 2E-02 | 4E-04 | 3.4 | L Claustrum |
|  |  | -52 | -24 | 18 | 2E-02 | 5E-04 | 3.3 | L Postcentral Gyrus BA40 |
|  |  | 44 | 0 | 10 | 2E-02 | 6E-04 | 3.3 | R Insula BA13 |
|  |  | 48 | -16 | 4 | 2E-02 | 6E-04 | 3.2 | R Insula BA13 |
|  |  | 52 | 12 | 0 | 2E-02 | 6E-04 | 3.2 | R Insula BA13 |
|  |  | 40 | 10 | 4 | 2E-02 | 7E-04 | 3.2 | R Insula BA13 |
|  |  | -50 | 30 | 16 | 2E-02 | 7E-04 | 3.2 | L Inferior Frontal Gyrus BA45 |
|  |  | -50 | -18 | 0 | 2E-02 | 8E-04 | 3.2 | L Superior Temporal Gyrus BA22 |
|  |  | -58 | 6 | 8 | 2E-02 | 8E-04 | 3.2 | L Precentral Gyrus BA6 |
|  |  | -44 | 6 | 28 | 2E-02 | 8E-04 | 3.1 | L Inferior Frontal Gyrus BA9 |
|  |  | 14 | -28 | -6 | 2E-02 | 1E-03 | 3.0 | R Thalamus |
|  |  | 46 | 20 | -4 | 2E-02 | 1E-03 | 3.0 | R Insula |
|  |  | -28 | -4 | 64 | 2E-02 | 1E-03 | 3.0 | L Middle Frontal Gyrus BA6 |
|  |  | 56 | -22 | 36 | 2E-02 | 2E-03 | 2.9 | R Postcentral Gyrus BA2 |
|  |  | 56 | 16 | -14 | 2E-02 | 2E-03 | 2.8 | R Superior Temporal Gyrus BA38 |
|  |  | -14 | 16 | 4 | 2E-02 | 3E-03 | 2.8 | L Caudate |
|  |  | 14 | -10 | -16 | 2E-02 | 3E-03 | 2.8 | R Parahippocampal Gyrus BA28 |
|  |  | -28 | -8 | 50 | 2E-02 | 4E-03 | 2.6 | L Middle Frontal Gyrus BA6 |
|  |  | 18 | 14 | -6 | 1E-02 | 5E-03 | 2.6 | R Lentiform Nucleus |
|  |  | -32 | -16 | 66 | 1E-02 | 5E-03 | 2.6 | L Precentral Gyrus BA4 |
|  |  | -38 | -46 | 56 | 1E-02 | 8E-03 | 2.4 | L Inferior Parietal Lobule BA40 |
|  |  | -28 | -56 | 54 | 1E-02 | 8E-03 | 2.4 | L Superior Parietal Lobule BA7 |
|  |  | 42 | -22 | 16 | 1E-02 | 9E-03 | 2.4 | R Insula BA13 |
|  |  | 20 | 8 | -16 | 1E-02 | 9E-03 | 2.3 | R Lentiform Nucleus |
|  |  | 42 | 22 | -16 | 1E-02 | 1E-02 | 2.2 | R Inferior Frontal Gyrus BA47 |
|  |  | 56 | -30 | 4 | 1E-02 | 2E-02 | 2.2 | R Superior Temporal Gyrus BA22 |
|  |  | 30 | -22 | 12 | 1E-02 | 2E-02 | 2.1 | R Claustrum |
|  |  | -52 | -8 | -16 | 1E-02 | 2E-02 | 2.1 | L Superior Temporal Gyrus BA21 |
|  |  | -54 | -36 | 0 | 1E-02 | 2E-02 | 2.1 | L Middle Temporal Gyrus |
|  |  | -50 | -14 | -12 | 1E-02 | 2E-02 | 2.1 | L Superior Temporal Gyrus BA22 |
|  |  | -50 | -46 | -4 | 1E-02 | 2E-02 | 2.0 | L Sub-Gyral BA37 |
|  |  | 30 | -14 | 8 | 1E-02 | 2E-02 | 2.0 | R Lentiform Nucleus |
|  |  | 18 | -24 | 18 | 1E-02 | 3E-02 | 1.9 | R Thalamus |
|  |  | -52 | 8 | 42 | 1E-02 | 3E-02 | 1.9 | L Middle Frontal Gyrus BA6 |
|  |  | -16 | -8 | 20 | 1E-02 | 3E-02 | 1.9 | L Caudate |
| *d. Left insula: 424 foci, 25 experiments, 354 subjects (x=-44, y=-10, z=-6)* | | | | | | | | |
| 1 | 49968 | -44 | -10 | -6 | 1E-01 | 0E+00 | 14.5 | L Insula BA13 |
|  |  | -64 | -28 | 6 | 2E-02 | 5E-06 | 4.4 | L Superior Temporal Gyrus BA22 |
|  |  | -40 | -24 | 20 | 2E-02 | 3E-05 | 4.0 | L Insula BA13 |
|  |  | -32 | 4 | 10 | 2E-02 | 3E-05 | 4.0 | L Claustrum |
|  |  | -50 | 6 | 0 | 2E-02 | 7E-05 | 3.8 | L Insula |
|  |  | -28 | -4 | -14 | 2E-02 | 9E-05 | 3.7 | L Parahippocampal Gyrus |
|  |  | -22 | 2 | 4 | 2E-02 | 1E-04 | 3.6 | L Lentiform Nucleus |
|  |  | -58 | -38 | 10 | 2E-02 | 2E-04 | 3.6 | L Superior Temporal Gyrus BA22 |
|  |  | -42 | -16 | 10 | 1E-02 | 3E-04 | 3.4 | L Insula BA13 |
|  |  | -36 | -34 | 12 | 1E-02 | 4E-04 | 3.3 | L Transverse Temporal Gyrus BA41 |
|  |  | -50 | -44 | 16 | 1E-02 | 7E-04 | 3.2 | L Superior Temporal Gyrus BA13 |
|  |  | -64 | -14 | 10 | 1E-02 | 8E-04 | 3.2 | L Transverse Temporal Gyrus BA42 |
|  |  | -54 | -24 | 24 | 1E-02 | 9E-04 | 3.1 | L Postcentral Gyrus BA40 |
|  |  | -42 | -6 | 10 | 1E-02 | 1E-03 | 3.1 | L Insula BA13 |
|  |  | -44 | 14 | 22 | 1E-02 | 2E-03 | 3.0 | L Inferior Frontal Gyrus BA9 |
|  |  | -32 | 24 | 2 | 1E-02 | 2E-03 | 2.9 | L Insula BA13 |
|  |  | -62 | -14 | 0 | 1E-02 | 4E-03 | 2.6 | L Superior Temporal Gyrus |
|  |  | -52 | -42 | 26 | 1E-02 | 5E-03 | 2.6 | L Inferior Parietal Lobule BA40 |
|  |  | -42 | -34 | 20 | 1E-02 | 5E-03 | 2.6 | L Superior Temporal Gyrus BA41 |
|  |  | -28 | -10 | 2 | 9E-03 | 6E-03 | 2.5 | L Lentiform Nucleus |
|  |  | -36 | 30 | -2 | 9E-03 | 7E-03 | 2.5 | L Inferior Frontal Gyrus BA47 |
|  |  | -42 | -26 | 2 | 9E-03 | 8E-03 | 2.4 | L Superior Temporal Gyrus BA22 |
|  |  | -50 | 28 | -4 | 9E-03 | 9E-03 | 2.4 | L Inferior Frontal Gyrus BA47 |
|  |  | -54 | 4 | 18 | 9E-03 | 1E-02 | 2.3 | L Inferior Frontal Gyrus BA44 |
|  |  | -38 | 8 | -20 | 8E-03 | 1E-02 | 2.3 | L Superior Temporal Gyrus BA38 |
|  |  | -44 | 28 | -2 | 8E-03 | 1E-02 | 2.3 | L Inferior Frontal Gyrus BA47 |
|  |  | -64 | -26 | 34 | 8E-03 | 1E-02 | 2.2 | L Inferior Parietal Lobule BA40 |
|  |  | -52 | -26 | 8 | 8E-03 | 1E-02 | 2.2 | L Superior Temporal Gyrus BA41 |
|  |  | -32 | 34 | -12 | 8E-03 | 2E-02 | 2.2 | L Inferior Frontal Gyrus BA47 |
|  |  | -16 | -6 | -16 | 8E-03 | 2E-02 | 2.1 | L Parahippocampal Gyrus BA28 |
|  |  | -62 | 4 | 6 | 6E-03 | 3E-02 | 1.8 | L Precentral Gyrus BA6 |
| 2 | 30128 | 56 | -26 | -2 | 2E-02 | 2E-06 | 4.6 | R Superior Temporal Gyrus BA22 |
|  |  | 60 | -6 | 0 | 2E-02 | 8E-06 | 4.3 | R Superior Temporal Gyrus BA22 |
|  |  | 50 | 14 | 4 | 2E-02 | 9E-06 | 4.3 | R Precentral Gyrus BA44 |
|  |  | 52 | -24 | 4 | 2E-02 | 1E-05 | 4.3 | R Superior Temporal Gyrus BA41 |
|  |  | 66 | -30 | 6 | 2E-02 | 1E-04 | 3.7 | R Superior Temporal Gyrus BA42 |
|  |  | 24 | 4 | 4 | 2E-02 | 1E-04 | 3.7 | R Lentiform Nucleus |
|  |  | 52 | -38 | 12 | 2E-02 | 1E-04 | 3.6 | R Superior Temporal Gyrus BA41 |
|  |  | 56 | -34 | 6 | 2E-02 | 2E-04 | 3.6 | R Superior Temporal Gyrus BA22 |
|  |  | 42 | -12 | -2 | 2E-02 | 2E-04 | 3.6 | R Claustrum |
|  |  | 56 | 4 | -6 | 1E-02 | 5E-04 | 3.3 | R Superior Temporal Gyrus BA22 |
|  |  | 36 | 24 | -2 | 1E-02 | 7E-04 | 3.2 | R Insula BA13 |
|  |  | 58 | 6 | -16 | 1E-02 | 8E-04 | 3.2 | R Superior Temporal Gyrus BA38 |
|  |  | 44 | 12 | -14 | 1E-02 | 9E-04 | 3.1 | R Extra-Nuclear BA13 |
|  |  | 46 | 22 | 10 | 1E-02 | 1E-03 | 3.0 | R Inferior Frontal Gyrus BA45 |
|  |  | 18 | 8 | -2 | 1E-02 | 1E-03 | 3.0 | R Lentiform Nucleus |
|  |  | 68 | -38 | 18 | 1E-02 | 3E-03 | 2.8 | R Superior Temporal Gyrus BA22 |
|  |  | 16 | -4 | -14 | 1E-02 | 3E-03 | 2.7 | R Parahippocampal Gyrus BA28 |
|  |  | 50 | 12 | 16 | 1E-02 | 4E-03 | 2.6 | No Gray Matter found |
|  |  | 62 | 12 | -6 | 9E-03 | 7E-03 | 2.5 | R Superior Temporal Gyrus BA22 |
|  |  | 34 | 8 | 12 | 8E-03 | 2E-02 | 2.2 | R Claustrum |
|  |  | 50 | -6 | -16 | 7E-03 | 2E-02 | 2.1 | R Sub-Gyral BA21 |
|  |  | 32 | 12 | -6 | 7E-03 | 3E-02 | 1.9 | R Lentiform Nucleus |
| 3 | 14888 | 8 | 4 | 64 | 1E-02 | 3E-04 | 3.5 | R Medial Frontal Gyrus BA6 |
|  |  | -8 | 16 | 52 | 1E-02 | 3E-04 | 3.4 | L Medial Frontal Gyrus BA6 |
|  |  | -2 | 0 | 66 | 1E-02 | 3E-04 | 3.4 | L Medial Frontal Gyrus BA6 |
|  |  | 4 | 14 | 46 | 1E-02 | 3E-04 | 3.4 | R Medial Frontal Gyrus BA32 |
|  |  | 0 | 4 | 46 | 1E-02 | 7E-04 | 3.2 | L Cingulate Gyrus BA24 |
|  |  | 0 | 12 | 34 | 1E-02 | 9E-04 | 3.1 | L Cingulate Gyrus BA24 |
|  |  | 6 | -10 | 64 | 1E-02 | 2E-03 | 2.8 | R Medial Frontal Gyrus BA6 |
|  |  | -6 | -8 | 50 | 1E-02 | 3E-03 | 2.7 | L Paracentral Lobule BA31 |
|  |  | -14 | -12 | 56 | 1E-02 | 5E-03 | 2.6 | L Medial Frontal Gyrus BA6 |
|  |  | -10 | -12 | 64 | 9E-03 | 6E-03 | 2.5 | L Precentral Gyrus BA6 |
|  |  | -4 | -10 | 70 | 9E-03 | 8E-03 | 2.4 | L Medial Frontal Gyrus BA6 |
|  |  | 2 | 14 | 64 | 9E-03 | 9E-03 | 2.4 | L Superior Frontal Gyrus BA6 |
|  |  | 10 | 4 | 36 | 9E-03 | 1E-02 | 2.3 | R Cingulate Gyrus BA24 |
|  |  | 6 | -10 | 48 | 8E-03 | 2E-02 | 2.1 | R Paracentral Lobule BA31 |
| GM, grey matter; WM, white matter; SU, substance user; HC, healthy control; ALE, anatomic likelihood estimation; P, p-value; Z, peak z-value; R, right; L, left. Addiction-related ROIs were created with Mango (<http://rii.uthscsa.edu/mango//userguide.html>) with a 5mm-radius sphere. | | | | | | | | |

| **Supplementary Table 8. Functional characterization of brain regions resulted from primary outcome according to BrainMap database.** | |
| --- | --- |
| *a. Right anterior cingulate cortex: 269 foci, 23 experiments, 332 subjects (x=8, y=48, z=0)* | |
| Action | Inhibition |
| Cognition | Attention, orthography, semantics, explicit memory, music reasoning, social cognition, somatic |
| Emotion | Anxiety, fear, reward |
| Interoception | Gastrointestinal/genitourinary, sexuality |
| Perception | Audition, gustation, olfaction, pain, vision |
| Paradigms | Classical conditioning, deception delayed match to sample, emotion induction, finger tapping/button pressing, go/no-go, meditation, micturition, music comprehension, olfactory monitor, pain monitor, paired associate recall, passive viewing, pitch monitor, reward, self-reflection, semantic monitor, sexual arousal/gratification, Stroop-color, taste, visual motion, visuospatial attention |
| *b. Left thalamus: 1025 foci, 48 experiments, 731 subjects (x=-6, y=-24, z=0)* | |
| Action | Execution, speech, inhibition, motor learning, observation, preparation, rest |
| Cognition | Attention, phonology, semantics, speech, syntax, explicit memory, working memory, music, reasoning, somatic |
| Emotion | Disgust, reward, valence |
| Interoception | Respiration regulation, sexuality |
| Perception | Audition, gustation, somesthesis, pain, vision, color |
| Paradigms | Affective pictures, chewing/swallowing, cued explicit recognition/recall, deception, delayed match to sample, emotion induction, emotional body language perception, encoding, episodic recall, face monitor/discrimination, film viewing, finger tapping/button press, flexion/extension, gambling, go/no-go, hand-eye coordination, meditation, motor learning, multi-tasking, music comprehension, oddball discrimination, pain monitor, paired associate recall, passive listening, passive viewing, phonological discrimination, pitch monitor/discrimination, pointing, reading (overt), reasoning/problem solving, recitation/repetition, reward, semantic monitor, sexual arousal/gratification, sleep, tactile monitor, taste, tone monitor, visual motion, visual pursuit, visuospatial attention, word generation (overt/covert) |
| *c. Left putamen: 1076 foci, 49 experiments, 727 subjects (x=-26, y=-4, z=2)* | |
| Action | Execution, speech, imagination, inhibition, motor learning |
| Cognition | Attention, language, explicit memory, working memory, music reasoning, social cognition, temporal |
| Emotion | Negative emotion, happiness, humor, reward |
| Interoception | Baroregulation, respiration regulation, sexuality, thermoregulation, vestibular |
| Perception | Audition, somesthesis, pain, vision-motion |
| Paradigms | Counting/calculation, delayed match to sample, driving, emotion induction, encoding, episodic recall, film viewing, finger tapping/button pressing, flexion/extension, go/no-go, grasping, hypercapnia, imagined movement, imagined objects/scenes, isometric force, motor learning, music comprehension, music production, orthographic discrimination, pain monitor, reading (overt/covert), recitation/repetition, reward, saccades, semantic monitor, sequence recall, Stroop-color, syntactic discrimination, tactile monitor, tone monitor, visual pursuit, visuospatial attention |
| *d. Left insula: 424 foci, 25 experiments, 354 subjects (x=-44, y=-10, z=-6)* | |
| Action | Execution |
| Cognition | Attention, Phonology, speech, working memory, music, somatic, spatial |
| Emotion | Intensity, sadness, negative emotion, happiness, valence |
| Interoception | Sexuality, vestibular |
| Perception | Audition, somesthesis, pain, vision |
| Paradigms | Acupuncture, affective pictures, cued explicit recognition/recall, emotion induction, face monitor, film viewing, finger tapping/button pressing, flexion/extension, go/no-go, music comprehension, music production, n-back, pain monitor, passive learning, passive viewing, pitch monitor, sexual arousal, tone monitor, transcranial magnetic stimulation, visuospatial attention, word generation (covert) |

Addiction-related ROIs were created with Mango (<http://rii.uthscsa.edu/mango//userguide.html>) with a 5mm-radius sphere.

| **Supplementary Table 9. FSN robustness assessment for significant ALE maps resulting from primary outcomes.** | | | | | | | | |
| --- | --- | --- | --- | --- | --- | --- | --- | --- |
| Cluster  number | Volume (mm^3^) | MNI coordinates | | | ALE | Label (Side, region) | Contributing | FNR |
|  |  | x | y | z |  |  | studies (k) |  |
| GM all foci | | | | | | | | |
| 1 | 23768 | 8 | 48 | 0 | 3E-02 | R Anterior Cingulate Cortex BA32 | 35 | >350 |
| 2 | 23256 | -6 | -24 | 0 | 4E-02 | L Thalamus | 30 | >300 |
| 3 | 22432 | -26 | -4 | 4 | 4E-02 | L Putamen | 20 | >200 |
| *GM volume decrease with use (SU < HC)* | | | | | | | | |
| 1 | 25360 | 8 | 48 | 0 | 3E-02 | R Anterior Cingulate Cortex BA32 | 20 | >200 |
| 2 | 22824 | -6 | -24 | 0 | 4E-02 | L Thalamus | 18 | 126 |
| 3 | 17064 | -44 | -10 | -6 | 2E-02 | L Insula BA13 | 16 | 82 |
| *GM volume increase with use (HC < SU)* | | | | | | | | |
| 1 | 22440 | -26 | -4 | 2 | 3E-02 | L Putamen | 30 | >300 |
| WM all foci | | | | | | | | |
| 1 | 37328 | 6 | -26 | -2 | 2E-02 | R Anterior thalamic radiation | 8 | >80 |
| *WM volume decrease with use (SU < HC)* | | | | | | | | |
| 1 | 33624 | 6 | -26 | -2 | 2E-02 | R Anterior thalamic radiation | 8 | >80 |
| *WM volume increase with use (HC < SU)* | | | | | | | | |
| 1 | 27736 | 14 | -14 | -16 | 2E-04 | R Corticospinal tract | 6 | 21 |
| 2 | 14712 | -46 | -6 | -30 | 8E-03 | L Superior longitudinal fasciculus | 5 | 19 |
| FSN, Fail-Safe N analysis | | | | | | | | |

| **Supplementary Table 10. Anatomic likelihood estimation meta-analytic results for studies comparing brain morphological changes between SU and HC at cluster level inference p < 0.05 (FWE). Sensitivity analysis including only studies with 3T scanner.** | | | | | | | | |
| --- | --- | --- | --- | --- | --- | --- | --- | --- |
| Cluster number | Volume (mm^3^) | MNI coordinates | | | ALE | P | Z | Label (Side region BA) |
|  |  | x | y | z |  |  |  |  |
| *a. GM: All foci* | | | | | | | | |
| 1 | 19808 | -24 | 16 | -8 | 1E-02 | 1E-03 | 3.0 | L Putamen |
| 2 | 12008 | 6 | 36 | -14 | 9E-03 | 8E-03 | 2.4 | R Anterior Cingulate Cortex BA32 |
| 3 | 10088 | -6 | -24 | 0 | 3E-02 | 1E-08 | 5.6 | L Thalamus |
| *b. GM: lower volume with use (SU < HC)* | | | | | | | | |
| 1 | 11664 | -8 | -66 | 16 | 2E-02 | 2E-06 | 4.6 | L Posterior Cingulate Cortex BA30 |
| 2 | 11240 | -46 | -8 | -10 | 1E-02 | 5E-04 | 3.3 | L Insula |
| 3 | 8192 | -6 | -24 | 0 | 3E-02 | 7E-09 | 5.7 | L Thalamus |
| *c. GM: Higher volume with use (HC < SU)* | | | | | | | | |
| 1 |  | -26 | -4 | 2 | 3E-02 | 4E-14 | 7.5 | L Putamen |
| *d. WM: All foci* | | | | | | | | |
| 1 |  | -6 | -24 | 0 | 3E-02 | 7E-09 | 5.7 | R Anterior thalamic radiation |
| *e. WM: Lower volume with use (SU < HC)* | | | | | | | | |
| 1 | 33624 | 6 | -26 | -2 | 2E-02 | 4E-07 | 4.9 | R Anterior thalamic radiation |
| *f. WM: Higher volume with use (HC < SU)* | | | | | | | | |
| 1 | 27736 | 14 | -14 | -16 | 2E-04 | 1E-02 | 2.3 | R Corticospinal tract |
| GM, grey matter; WM, white matter; SU, substance user; HC, healthy control; ALE, anatomic likelihood estimation; P, p-value; Z, peak z-value; R, right; L, left. | | | | | | | | |

**References**

1. Higgins, J. P. & Green, S. Cochrane Handbook for Systematic Reviews of Interventions. (2011).

2. Moher, D., Liberati, A., Tetzlaff, J., Altman, D. G. & Group, T. P. Preferred Reporting Items for Systematic Reviews and Meta-Analyses: The PRISMA Statement. **6**, 1–5 (2009).

3. Covidence systematic review software, Veritas Health Innovation, Melbourne, Australia. Available at www.covidence.org. Available at: https://support.covidence.org/help/how-can-i-cite-covidence.

4. Laird, A. R. *et al.* Networks of task co-activations. *Neuroimage* **80**, 505–514 (2013).

5. Laird, A. R. *et al.* *User Manual for Sleuth 2.0*.

6. Fox, P. T. *et al.* *User Manual for GingerALE 2.3*.

**Citations of included studies (n=60)**

Almeida, O. P., Garrido, G. J., Lautenschlager, N. T., Hulse, G. K., Jamrozik, K., Phil, D., & Flicker, L. (2008). Smoking Is Associated With Reduced Cortical Regional Gray Matter Density in Brain Regions Associated With Incipient Alzheimer Disease. *American Journal of Geriatric Psychiatry*, *16*, 92–98. https://doi.org/10.1097/JGP.0b013e318157cad2

Aoki, Y., Orikabe, L., Takayanagi, Y., Yahata, N., Mozue, Y., Sudo, Y., … Yamasue, H. (2013). Volume reductions in frontopolar and left perisylvian cortices in methamphetamine induced psychosis. *Schizophrenia Research*, *147*, 355–361. https://doi.org/10.1016/j.schres.2013.04.029

Bach, P. *et al.* Impaired working memory performance in opioid-dependent patients is related to reduced insula gray matter volume: a voxel-based morphometric study. *Eur. Arch. Psychiatry Clin. Neurosci.* **1**, 3

Bach, P. *et al.* Higher Social Rejection Sensitivity in Opioid-Dependent Patients Is Related to Smaller Insula Gray Matter Volume: A Voxel-Based Morphometric Study. *Soc. Cogn. Affect. Neurosci.* **14**, 1187–1195 (2019).

Banca, P., Lange, I., Worbe, Y., Howell, N. A., Irvine, M., Harrison, N. A., … Voon, V. (2016). Reflection impulsivity in binge drinking: behavioural and volumetric correlates. *Addiction Biology*.https://doi.org/10.1111/adb.12227

Barrós-Loscertales, A., Garavan, H., Bustamante, J. C. J. C., Ventura-Campos, N., Llopis, J. J. J. J., Belloch, V., … Ávila, C. (2011). Reduced striatal volume in cocaine-dependent patients. *NeuroImage*, *56*(3). https://doi.org/10.1016/j.neuroimage.2011.02.035

Battistella, G., Fornari, E., Annoni, J.-M. J.-M., Chtioui, H., Dao, K., Fabritius, M., … Giroud, C. (2014). Long-Term Effects of Cannabis on Brain Structure Giovanni. *Neuropsychopharmacology*, *39*. https://doi.org/10.1038/npp.2014.67

Brody, A. L., Mandelkern, M. A., Jarvik, M. E., Lee, G. S., Smith, E. C., Huang, J. C., … London, E. D. (2004). Differences between Smokers and Nonsmokers in Regional Gray Matter Volumes and Densities. *Biol Psychiatry*, *55*, 77–84. https://doi.org/10.1016/S0006-3223(03)00610-3

Bu, L., Yu, D., Su, S., Ma, Y., von Deneen, K. M., Luo, L., … Deneen, von K. (2016). Functional Connectivity Abnormalities of Brain Regions with Structural Deficits in Young Adult Male Smokers. *Frontiers in Human Neuroscience*. https://doi.org/10.3389/fnhum.2016.00494

Chanraud, S., Leroy, C., Martelli, C., Kostogianni, N., Delain, F., Aubin, H.-J., … Martinot, J.-L. (2009). Episodic Memory in Detoxified Alcoholics: Contribution of Grey Matter Microstructure Alteration. *PLoS ONE*, *4*(8). https://doi.org/10.1371/journal.pone.0006786

Chanraud, S., Martelli, C., Delain, F., Kostogianni, N., Douaud, G., Aubin, H.-J., … Martinot, J.-L. (2007). Brain Morphometry and Cognitive Performance in Detoxified Alcohol-Dependents with Preserved Psychosocial Functioning. *Neuropsychopharmacology*. https://doi.org/10.1038/sj.npp.1301219

Crunelle, C. L., Kaag, A. M., van Wingen, G., van den Munkhof, H. E., Homberg, J. R., Reneman, L., & van den Brink, W. (2014). Reduced frontal brain volume in non-treatment-seeking cocaine-dependent individuals: Exploring the role of impulsivity, depression, and smoking. *Frontiers in Human Neuroscience*, *8*(JAN). https://doi.org/10.3389/fnhum.2014.00007

Daumann, J., Koester, P., Becker, B., Wagner, D., Imperati, D., Gouzoulis-Mayfrank, E., & Tittgemeyer, M. (2011). Medial prefrontal gray matter volume reductions in users of amphetamine-type stimulants revealed by combined tract-based spatial statistics and voxel-based morphometry. *NeuroImage*, *54*(2). https://doi.org/10.1016/j.neuroimage.2010.08.065

Demirakca, T., Ende, G., Kämmerer, N., Welzel-Marquez, H., Hermann, D., Heinz, A., & Mann, K. (2011). Effects of alcoholism and continued abstinence on brain volumes in both genders. *Alcoholism: Clinical and Experimental Research*, *35*(9). https://doi.org/10.1111/j.1530-0277.2011.01514.x

Filbey, F. M., Aslan, S., Calhoun, V. D., Spence, J. S., Damaraju, E., Caprihan, A., & Segall, J. (2014). Long-term effects of marijuana use on the brain. *Proceedings of the National Academy of Sciences of the United States of America*, *111*(47). https://doi.org/10.1073/pnas.1415297111

Franklin, T.R., Acton, P. D., Maldjian, J. A., Gray, J. D., Croft, J. R., Dackis, C. A., … Childress, A. R. (2002). Decreased gray matter concentration in the insular, orbitofrontal, cingulate, and temporal cortices of cocaine patients. *Biological Psychiatry*, *51*(2). https://doi.org/10.1016/S0006-3223(01)01269-0

Franklin, Teresa R, Wetherill, R. R., Jagannathan, K., Johnson, B., Mumma, J., Hager, N., … Zang, Y.-F. (2014). The Effects of Chronic Cigarette Smoking on Gray Matter Volume: Influence of Sex. *PLoS ONE*, *9*(8). https://doi.org/10.1371/journal.pone.0104102

Fritz, H.-C., Wittfeld, K., Schmidt, C. O., Domin, M., Grabe, H. J., Hegenscheid, K., … Lotze, M. (2014). Current Smoking and Reduced Gray Matter Volume—a Voxel-Based Morphometry Study. *Neuropsychopharmacology*, *39*(10), 2594–2600. https://doi.org/10.1038/npp.2014.112

Galandra, C. *et al.* Salience network structural integrity predicts executive impairment in alcohol use disorders. *Sci. Rep.* **8**, 14481 (2018).

Galandra, C. *et al.* Decreased information processing speed and decision-making performance in alcohol use disorder: combined neurostructural evidence from VBM and TBSS. *Brain Imaging Behav.* 1–11 (2020). doi:10.1007/s11682-019-00248-8

Gallinat, J., Meisenzahl, E., Jacobsen, L. K., Kalus, P., Bierbrauer, J., Kienast, T., … Staedtgen, M. (2006). Smoking and structural brain deficits: A volumetric MR investigation. *European Journal of Neuroscience*, *24*(6). https://doi.org/10.1111/j.1460-9568.2006.05050.x

Gardini, S., & Venneri, A. (2012). Reduced grey matter in the posterior insula as a structural vulnerability or diathesis to addiction. *Brain Research Bulletin*, *87*(2–3). https://doi.org/10.1016/j.brainresbull.2011.11.021

Gilman, J. M., Kuster, J. K., Lee, S., Lee, M. J., Kim, B. W., Makris, N., … Breiter, H. C. (2014). Cannabis use is quantitatively associated with nucleus accumbens and amygdala abnormalities in young adult recreational users. *Journal of Neuroscience*, *34*(16). https://doi.org/10.1523/JNEUROSCI.4745-13.2014

Grodin, E. N., Lin, H., Durkee, C. A., Hommer, D. W., & Momenan, R. (2013). Deficits in cortical, diencephalic and midbrain gray matter in alcoholism measured by VBM: Effects of co-morbid substance abuse ☆. *YNICL*, *2*, 469–476. https://doi.org/10.1016/j.nicl.2013.03.013

Hanlon, C. A. *et al.* Lower subcortical gray matter volume in both younger smokers and established smokers relative to non-smokers. (2014). doi:10.1111/adb.12171

Hanlon, C. A., Owens, M. M., Joseph, J. E., Zhu, X., George, M. S., Brady, K. T., & Hartwell, K. J. (2016). Lower subcortical gray matter volume in both younger smokers and established smokers relative to non-smokers HHS Public Access. *Addict Biol*, *21*(1), 185–195. https://doi.org/10.1111/adb.12171

Jan, R. K., Lin, J. C., Miles, S. W., Kydd, R. R., & Russell, B. R. (2012). Striatal Volume Increases in Active Methamphetamine-Dependent Individuals and Correlation with Cognitive Performance. *Brain Sci*, *2*, 553–572. https://doi.org/10.3390/brainsci2040553

Jang, D.-P., Namkoong, K., Kim, J.-J., Park, S., Kim, I.-Y., Kim, S. I., … Lee, E. (2007). The relationship between brain morphometry and neuropsychological performance in alcohol dependence. *Neuroscience Letters*, *428*, 21–26. https://doi.org/10.1016/j.neulet.2007.09.047

Li, J. *et al.* Whole-brain morphometric studies in alcohol addicts by voxel-based morphometry. *Ann. Transl. Med.* **7**, 635–635 (2019).Liao, Y., Tang, J., Corlett, P. R., Wang, X., Yang, M., Chen, H., … Fletcher, P. C. (2011). Reduced Dorsal Prefrontal Gray Matter After Chronic Ketamine Use. *Biol Psychiatry*. https://doi.org/10.1016/j.biopsych.2010.08.030

Liao, Y. *et al.* Reduced dorsal prefrontal gray matter after chronic ketamine use. *Biol. Psychiatry* **69**, (2011).

Liao, Y., Tang, J., Liu, T., Chen, X., & Hao, W. (2012). Differences between smokers and non-smokers in regional gray matter volumes: A voxel-based morphometry study. *Addiction Biology*, *17*(6), 977–980. https://doi.org/10.1111/j.1369-1600.2010.00250.x

Lin, W.-C., Chou, K.-H., Chen, H.-L., Huang, C.-C., Lu, C.-H., Li, S.-H., … Chen, C.-C. (2012). Structural deficits in the emotion circuit and cerebellum are associated with depression, anxiety and cognitive dysfunction in methadone maintenance patients: A voxel-based morphometric study. *Psychiatry Research: Neuroimaging*. https://doi.org/10.1016/j.pscychresns.2011.05.009

Liu, H., Hao, Y., Kaneko, Y., Ouyang, X., Zhang, Y., Xu, L., … Liu, Z. (2009). Frontal and cingulate gray matter volume reduction in heroin dependence: Optimized voxel-based morphometry: Regular article. *Psychiatry and Clinical Neurosciences*, *63*(4), 563–568. https://doi.org/10.1111/j.1440-1819.2009.01989.x

Lyoo, I. K., Pollack, M. H., Silveri, M. M., Kyung, H. A., Diaz, C. I., Hwang, J., … Renshaw, P. F. (2006). Prefrontal and temporal gray matter density decreases in opiate dependence. *Psychopharmacology*, *184*(2). https://doi.org/10.1007/s00213-005-0198-x

Mackey, S., Stewart, J. L., Connolly, C. G., Tapert, S. F., & Paulus, M. P. (2014). A Voxel-Based Morphometry Study of Young Occasional Users of Amphetamine-Type Stimulants and Cocaine. *Drug Alcohol Depend. February*, *111*(135). https://doi.org/10.1016/j.drugalcdep.2013.11.018

Matochik, J. A., Eldreth, D. A., Cadet, J.-L., & Bolla, K. I. (2005). Altered brain tissue composition in heavy marijuana users. *Drug and Alcohol Dependence*, *77*(1). https://doi.org/10.1016/j.drugalcdep.2004.06.011

Matuskey, D., Bhagwagar, Z., Planeta, B., Pittman, B., Gallezot, J.-D., Chen, J., … Malison, R. T. (2014). Reductions in brain 5-HT1B receptor availability in primarily cocaine-dependent humans. *Biological Psychiatry*, *76*(10). https://doi.org/10.1016/j.biopsych.2013.11.022

Meade, C. S., Bell, R. P., Towe, S. L. & Hall, S. A. Cocaine-related alterations in fronto-parietal gray matter volume correlate with trait and behavioral impulsivity. *Drug Alcohol Depend.* **206**, 107757 (2020).

Mechtcheriakov, S., Brenneis, C., Egger, K., Koppelstaetter, F., Schocke, M., & Marksteiner, J. (2007). A widespread distinct pattern of cerebral atrophy in patients with alcohol addiction revealed by voxel-based morphometry. *Journal of Neurology, Neurosurgery and Psychiatry*, *78*(6). https://doi.org/10.1136/jnnp.2006.095869

Morales, A., Lee, B., Hellemann, G., Neill, J. O. ’, & London, E. D. (2012). Gray-Matter Volume in Methamphetamine Dependence: Cigarette Smoking and Changes with Abstinence from Methamphetamine*. *Drug Alcohol Depend*, *125*(3), 230–238. https://doi.org/10.1016/j.drugalcdep.2012.02.017

Moreno-Alcázar, A. *et al.* Larger Gray Matter Volume in the Basal Ganglia of Heavy Cannabis Users Detected by Voxel-Based Morphometry and Subcortical Volumetric Analysis. *Front. Psychiatry* **9**, 175 (2018).

Moreno-López, L., Catena, A., Fernández-Serrano, M. J., Delgado-Rico, E., Stamatakis, E. A., Pérez-García, M., & Verdejo-García, A. (2012). Trait impulsivity and prefrontal gray matter reductions in cocaine dependent individuals. *Drug and Alcohol Dependence*, *125*, 208–214. https://doi.org/10.1016/j.drugalcdep.2012.02.012

Mwansisya, T. E., Zhang, H., Wang, Z., Wu, G., Hu, A., Wang, P., … Liu, Z. (2016). Major depressive disorder and heroin-dependent patients share decreased frontal gray matter volumes: A voxel-based morphometry study. *International Journal of Emergency Mental Health*, *18*(1).

Noyan, C. O., Kose, S., Nurmedov, S., Metin, B., Darcin, A. E., Dilbaz, N., & Noyan CO.; Kose S.; Nurmedov S.; Metin B.; Darcin AE.; (2016). Volumetric brain abnormalities in polysubstance use disorder patients. *Neuropsychiatric Disease and Treatment*, *12*. https://doi.org/10.2147/NDT.S107733

Nurmedov, S., Metin, B., Ekmen, S., Noyan, O., Yilmaz, O., Darcin, A., … Hospital, N. N. (2015). Thalamic and Cerebellar Gray Matter Volume Reduction in Synthetic Cannabinoids Users brain of synthetic cannabinoids users, and point the need for further investigation of morphological effects of synthetic cannabinoids in the brain. *Eur Addict Res*, *21*, 315–320. https://doi.org/10.1159/000430437

Nurmedov, S., Noyan, O., Metin, B., Ekmen, S., Avcil, C., & Kose, S. (2016). Extensive gray matter volume reduction and correlations with neuropsychological performance in alcohol use disorder patients, *26*(4), 355. https://doi.org/10.5455/bcp.20160223123949

Peng, P., Wang, Z., Jiang, T., Chu, S., Wang, S., & Xiao, D. (2015). Brain-volume changes in young and middle-aged smokers: a DARTEL-based voxel-based morphometry study. *Clinical Respiratory Journal*, *11*(5), 621–631. https://doi.org/10.1111/crj.12393

Peng, P. *et al.* Brain Structure Alterations in Respect to Tobacco Consumption and Nicotine Dependence: A Comparative Voxel-Based Morphometry Study. *Front. Neuroanat.* **12**, 43 (2018).

Potvin, S., Mancini-Marïe, A., Fahim, C., Mensour, B., Lévesque, J., Karama, S., … Stip, E. (2007). Increased striatal gray matter densities in patients with schizophrenia and substance use disorder: A voxel-based morphometry study. *Psychiatry Research: Neuroimaging*. https://doi.org/10.1016/j.pscychresns.2006.11.009

Qiu, Y.-W., Lv, X.-F., Jiang, G.-H., Su, H.-H., Yu, T., Tian, J.-Z., … Zhuo, F.-Z. (2014). Reduced ventral medial prefrontal cortex (vmPFC) volume and impaired vmPFC-default mode network integration in codeine-containing cough syrups users. *Drug and Alcohol Dependence*, *134*(1). https://doi.org/10.1016/j.drugalcdep.2013.10.023

Qiu, Ying-Wei, Jiang, G.-H., Su, H.-H., Lv, X.-F., Tian, J.-Z., Li, L.-M., & Zhuo, F.-Z. (2013). The impulsivity behavior is correlated with prefrontal cortex gray matter volume reduction in heroin-dependent individuals. *Neuroscience Letters*, *538*(538), 43–48. https://doi.org/10.1016/j.neulet.2013.01.019

Segobin, S. H., Chételat, G., Le Berre, A. P., Lannuzel, C., Boudehent, C., Vabret, F., … Pitel, A. L. (2014). Relationship between brain volumetric changes and interim drinking at six months in alcohol-dependent patients. *Alcoholism: Clinical and Experimental Research*, *38*(3), 739–748. https://doi.org/10.1111/acer.12300

Sim, M. E., Lyoo, I. K., Streeter, C. C., Covell, J., Sarid-Segal, O., Ciraulo, D. A., … Renshaw, P. F. (2007). Cerebellar gray matter volume correlates with duration of cocaine use in cocaine-dependent subjects. *Neuropsychopharmacology*, *32*(10). https://doi.org/10.1038/sj.npp.1301346

Stoeckel, L. E., Chai, X. J., Zhang, J., Whitfield-Gabrieli, S., & Evins, A. E. (2016). Lower gray matter density and functional connectivity in the anterior insula in smokers compared with never smokers. *Addiction Biology*, *21*(4). https://doi.org/10.1111/adb.12262

van Eijk, J., Demirakca, T., Frischknecht, U., Hermann, D., Mann, K., & Ende, G. (2013). Rapid Partial Regeneration of Brain Volume During the First 14 Days of Abstinence from Alcohol. *Alcoholism: Clinical and Experimental Research*, *37*(1). https://doi.org/10.1111/j.1530-0277.2012.01853.x

van Holst, R. J., de Ruiter, M. B., van den Brink, W., Veltman, D. J., & Goudriaan, A. E. (2012). A voxel-based morphometry study comparing problem gamblers, alcohol abusers, and healthy controls. *Drug and Alcohol Dependence*, *124*(1–2). https://doi.org/10.1016/j.drugalcdep.2011.12.025

Wang, J. *et al.* Alterations in Brain Structure and Functional Connectivity in Alcohol Dependent Patients and Possible Association with Impulsivity. *PLoS One* **11**, e0161956 (2016).

Wetherill, R. R., Jagannathan, K., Hager, N., Childress, A. R., Rao, H., & Franklin, T. R. (2015). Cannabis, cigarettes, and their co-occurring use: Disentangling differences in gray matter volume. *International Journal of Neuropsychopharmacology*, *18*(10). https://doi.org/10.1093/ijnp/pyv061

Yip, S. W., Worhunsky, P. D., Xu, J., Morie, K. P., Constable, R. T., Malison, R. T., … Potenza, M. N. (2017). Gray-matter relationships to diagnostic and transdiagnostic features of drug and behavioral addictions. *Addiction Biology*. https://doi.org/10.1111/adb.12492

Zhang, X., Salmeron, B. J., Ross, T. J., Geng, X., Yang, Y., & Stein, E. A. (2011). Factors underlying prefrontal and insula structural alterations in smokers. *NeuroImage*, *54*(1). https://doi.org/10.1016/j.neuroimage.2010.08.008
